# Identification of lignin oligomers in Kraft lignin using ultra-high-performance liquid chromatography/high-resolution multiple-stage tandem mass spectrometry (UHPLC/HRMS^n^)

**DOI:** 10.1007/s00216-018-1400-4

**Published:** 2018-10-10

**Authors:** Jens Prothmann, Peter Spégel, Margareta Sandahl, Charlotta Turner

**Affiliations:** Department of Chemistry, Centre for Analysis and Synthesis, Lund University, P.O. Box 124, 22100 Lund, Sweden

**Keywords:** Classification model, Design of experiment, MS ionisation efficiency, Neutral loss scan, Phenolic compounds, Lignin

## Abstract

Kraft lignin is the main source of technically produced lignin. For the development of valuable products based on Kraft lignin, its molecular structure is important. However, the chemical composition of Kraft lignin is still not well known. So far, the analysis of Kraft lignin by mass spectrometry (MS) has been mainly focused on monomeric compounds. Previous MS studies on lignin oligomers (LOs) considered only synthesised LO standards and/or lignins produced by processes other than the Kraft process. Furthermore, published MS methods suffer from using high resolution only in the MS^1^ stage in multiple-stage tandem MS methods. A high resolution in all MS^n^ stages would provide more detailed information about LO fragmentation pathways. Since lignin samples are complex mixtures of a large number of similar phenolic compounds, the selection of tentative LOs in the MS data is challenging. In this study, we present a method for non-targeted analysis of LOs in Kraft lignin using ultra-high-performance liquid chromatography/high-resolution multiple-stage tandem mass spectrometry (UHPLC/HRMS^n^). A pre-selection strategy for LOs has been established based on a data-dependent neutral loss MS^3^ method in combination with a principal component analysis-quadratic discriminant analysis classification model (PCA-QDA). The method was optimised using a design of experiments (DOE) approach. The developed approach improved the pre-selection of tentative LOs in complex mixtures. From 587 detected peaks, 36 peaks were identified as LOs.

**Electronic supplementary material:**

The online version of this article (10.1007/s00216-018-1400-4) contains supplementary material, which is available to authorized users.

**Graphical abstract:**
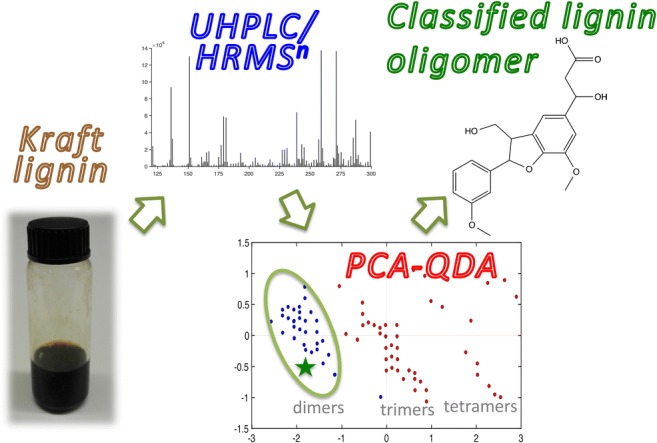
ᅟ

## Introduction

**Fig. 1:**
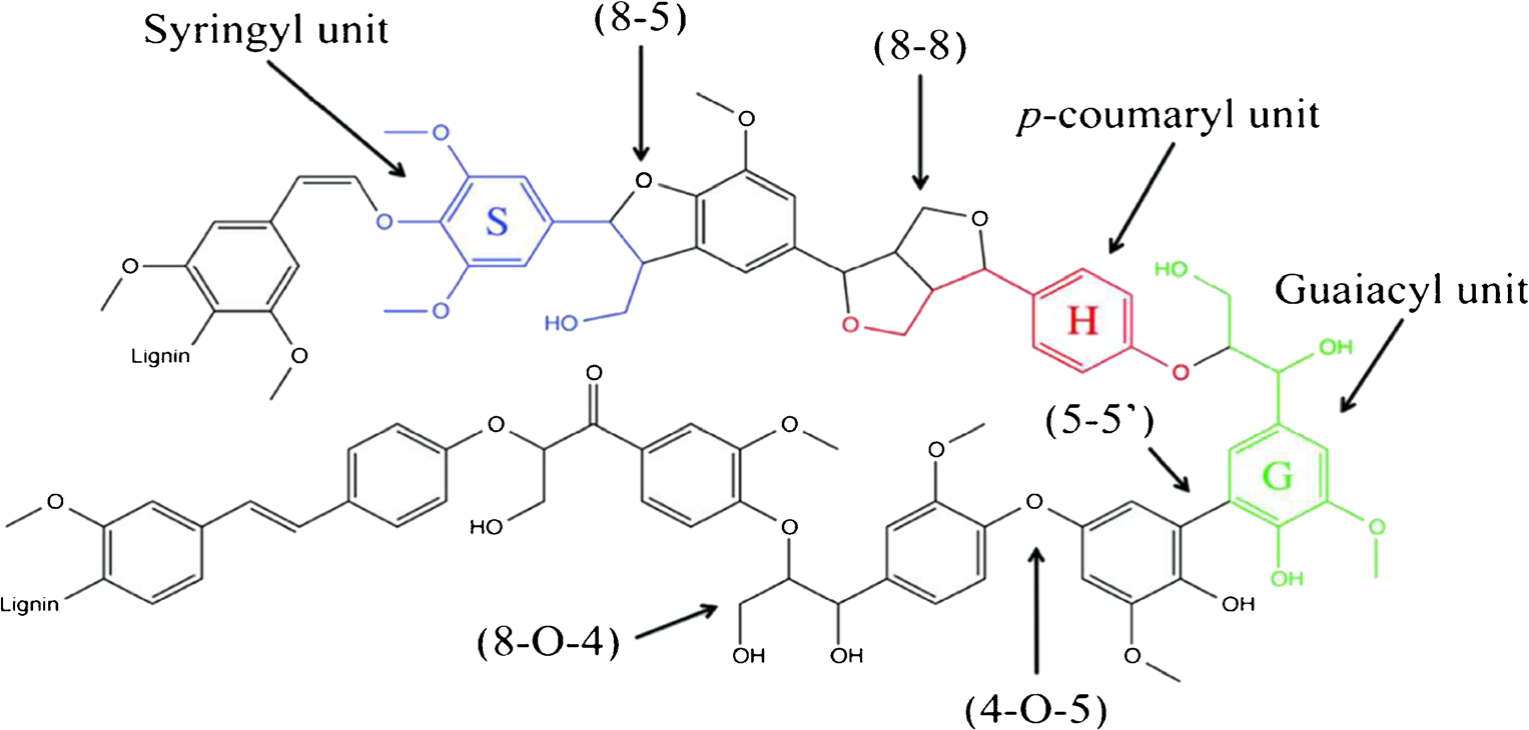
Tentative lignin structure with labeled subunits and linkages

The aromatic biopolymer lignin is a promising biorenewable raw material, which has the potential to reduce our dependency on crude oil. The aromatic nature of lignin offers many opportunities to use it as a source for high value aromatic chemicals and for the production of biofuels, functional polymers or various industrial materials [1, 2]. The lignin biopolymer has a complex molecular structure. It is formed via oxidative radicalisation of the three main monomeric phenolic subunits sinapyl alcohol (S-unit), coniferyl alcohol (G-unit) and p-coumaryl alcohol (H-unit) that are connected through ether and carbon-carbon covalent bonds (Fig. 1) [2, 3]. The most abundant lignin linkage is the 8-aryl-ether linkage (8-O-4), followed by the resinol linkage (8-8), phenylcoumaran linkage (8-5), 5-5′ linkage and the 4-O-5 linkage (Fig. 1) [4]. The main technically produced lignin is Kraft lignin [1]. Because of its high availability, as a byproduct of the pulp and paper industry, utilisation of Kraft lignin has become a research focus during the last years [5]. In the Kraft pulping process, lignin is separated from the cellulose and hemicellulose by alkaline treatment of wood chips. The results are mainly in cleavage of aryl ether linkages, but many other transformations of the chemical structure may also take place [4, 6]. Different depolymerisation and biological conversion methods for Kraft lignin have been investigated to produce new valuable molecules [1, 7]. For an easier classification of different types of lignin samples, Banoub et al. introduced the terms virgin released lignins (VRLs), for lignin obtained by chemical hydrolysis and/or enzymatic hydrolysis, and processed modified lignins (PMLs) obtained by techniques like the Kraft lignin process that cause more chemical transformations of the lignin biopolymer [8].

For the valorisation of Kraft lignin, detailed knowledge about its chemical composition is important. The identification of lignin-derived compounds in Kraft lignin using MS has mainly focused on monomeric compounds using pyrolysis-gas chromatography (Py-GC)/MS [9, 10]. Moreover, often only depolymerisation products of Kraft lignin were investigated, instead of the raw Kraft lignin. Nuclear magnetic resonance spectroscopy (NMR) allows for determination of functional groups and interunit linkages, and has therefore been extensively used to investigate the chemical composition of Kraft lignin [11, 12]. However, with techniques like Py-GC/MS and NMR, it is difficult to identify and characterise single lignin oligomers (LOs) in complex Kraft lignin samples. In recent years, several methods based on atmospheric pressure ionisation (API) in combination with tandem mass spectrometry (MS/MS) or multiple-stage tandem mass spectrometry (MS^n^) have been established for the investigation of LOs in different types of lignin samples [8, 13].

A main advantage of MS/MS or MS^n^ over for example Py-GC/MS or NMR is the possibility of investigating the chemical structure of LOs based on their MS fragmentation pathways. In combination with liquid chromatography (LC) and mass analysers with high mass resolution, several identification methods for LOs have been established.

Direct infusion (DI) and LC-MS/MS and MS^n^ methods have been developed using VRLs from several lignin sources [14–20]. Different API sources, such as atmospheric pressure chemical ionisation (APCI) [14], atmospheric pressure photo ionisation (APPI) [16] and electrospray ionisation (ESI) [15, 17–20], have been applied. Also, several different mass analysers, such as the triple quadrupole (QQQ) [17], ion trap (IT) [14], quadrupole/time-of-flight (QToF) [16] and linear-ion-trap/Fourier-transform-ion-cyclotron-resonance (LIT-FT-ICR) [15, 18–20], have been used. While DI-MS^n^ has been performed with MS^n^ stages higher than MS^3^ [15], LC/MS^n^ for LOs have just been performed until MS^3^ [18–20]. In all previous MS/MS or MS^n^ methods, high-resolution MS was only used in the MS^1^ stage or until the MS^2^ stage. However, a high resolution in all MS^n^ stages, which is achievable using a LIT-Orbitrap hybrid MS [21, 22], would yield more detailed information about the fragmentation pathways of LOs. With high resolution in every MS^n^ stage, chemical formulas and ring double bond (RDB) equivalents of all fragments can be determined. The RDB equivalent provides information about the degree of unsaturation of an organic molecule, for example a double bond or a ring structure has a RDB equivalent of 1, while a triple bond has a RDB equivalent of 2 [23]. Hence, the RDB equivalent is a helpful tool for proposing chemical structures based on MS fragmentation pathways. Moreover, the identification confidence of tentative LOs can be improved by introducing pre-selection tools into the analysis strategy. So far, only Jarrell et al. have applied a classification strategy for lignin-related monomers, dimers and lignin-carbohydrate complexes based on their C:O ratio, m/z ranges and RDB equivalent ranges [20]. Jarrell et al. used a cut off value of 250 Da to discriminate between monomers (< 250 Da) and dimers (250 up to 400 Da) [20]. The sharp cut off of 250 Da between monomers and dimers might be reasonable since no dimers with a mass lower as 250 Da are known. However, discrimination between trimers, tetramers and higher order oligomers, which are more complex, based on a sharp mass cut-off is unfeasible. Instead, a multivariate classification approach, with no sharp cut offs, may allow for discrimination between these. The approach may be even more powerful when combined with a neutral loss screening to reduce the number of suspected LOs. Characteristic neutral losses for specific LO-linkages are known from studies by Morreel et al. [14]. Recently, Dator et al. developed a HR-data-dependent neutral loss-MS^3^ approach for carbonyl compounds in salvia [24]. This approach can be adapted for LOs and used as a pre-selection tool.

Currently, no LC/HRMS^n^ method for identification of LOs in PML Kraft lignin has been established. Presumably, this is due to the higher complexity of Kraft lignin compared to VRLs. In addition, no systematic optimisation of a LC/MS method for analysis of LOs, independent of the lignin source, has been reported yet.

In this study, we present a non-targeted UHPLC/HRMS^n^ approach for identification of LOs in Kraft lignin. The identification confidence for LOs is improved by introducing two pre-selection tools: an HR-data-dependent neutral loss MS^3^ in combination with a principal component analysis-quadratic discriminant analysis (PCA-QDA) classification model for LOs. High resolution in all MS^n^ stages was ensured using a LIT-Orbitrap MS system. UHPLC column screening, UHPLC gradient optimisation and design of experiments (DOE)-based optimisation of the MS ionisation source setting were performed using identified LOs in the sample. Finally, structures of identified LOs were suggested based on UHPLC/HRMS^n^ experiments.

## Materials and methods

### Chemicals

Guaiacylglycerol-beta-guaiacyl ether, ammonium formate and uracil were purchased from Sigma-Aldrich (St. Louis, MO, USA). Acetone (HPLC grade) and ammonia (2 M solution in methanol) were obtained from Thermo Fisher Scientific (Waltham, MA, USA). Acetonitrile (HPLC/MS grade) was purchased from VWR Chemicals (Radnor, PA, USA). Purified water was obtained from a Milli-Q Water Purification System with a UV unit.

### Kraft lignin sample and sample preparation

Pine softwood Kraft lignin (Indulin AT from Charleston Heights, SC, USA) was kindly provided by Christian Hulteberg and Omar Y. Abdelaziz. A stock solution of Kraft lignin (200 mg/mL) was prepared by dissolving 0.5 g Kraft lignin in 2.5 mL acetone/water (70/30; *v*/*v*). The stock solution was diluted with acetonitrile/water (50/50; *v*/*v*) to a final concentration of 33.3 mg/mL. Afterwards, the sample was centrifuged for 10 min at 14,000 rpm and 20 °C. The supernatant was collected for further analysis.

### Equipment

All experiments were performed on a Thermo Scientific Accela UHPLC system with an Accela 600 Pump, Accela Autosampler and Accela PDA Detector coupled to a Thermo Fisher Scientific LTQ Orbitrap Velos Pro mass spectrometer (Thermo Scientific, Waltham, MA, USA). All samples were centrifuged using a 5424R Eppendorf centrifuge (Eppendorf, Hamburg, Germany). All ACQUITY UPLC columns (BEH C18 (2.1 mm × 100 mm, 1.7 μm, 130 Å), BEH Phenyl (2.1 mm × 100 mm, 1.7 μm, 130 Å) and CSH Phenyl-Hexyl (2.1 mm × 100 mm, 1.7 μm, 130 Å)) and all ACQUITY UPLC VanGuard pre-columns (BEH C18 (2.1 mm × 5 mm, 1.7 μm, 130 Å), BEH Phenyl (2.1 mm × 5 mm, 1.7 μm, 130 Å) and CSH Phenyl-Hexyl (2.1 mm × 5 mm, 1.7 μm, 130 Å)) were purchased from Waters (Milford, MA, USA).

### Software

The UHPLC/MS system was operated and data acquired using Xcalibur 2.2 (Thermo Fisher Scientific). Xcalibur 2.2 and the open-source software MZmine 2 were used for data evaluation. Experimental designs were created and evaluated in Modde™ 10.1.0 software (Sartorious-Stedim, Umeå, Sweden). Classification models were created in MATLAB (MathWorks, Natick, MA, USA) using the Classification toolbox for MATLAB from the Milano Chemometrics and QSAR Research Group (University of Milan, Milan, Italy).

### Overview of a novel strategy for non-targeted analysis of lignin oligomers

**Fig. 2:**
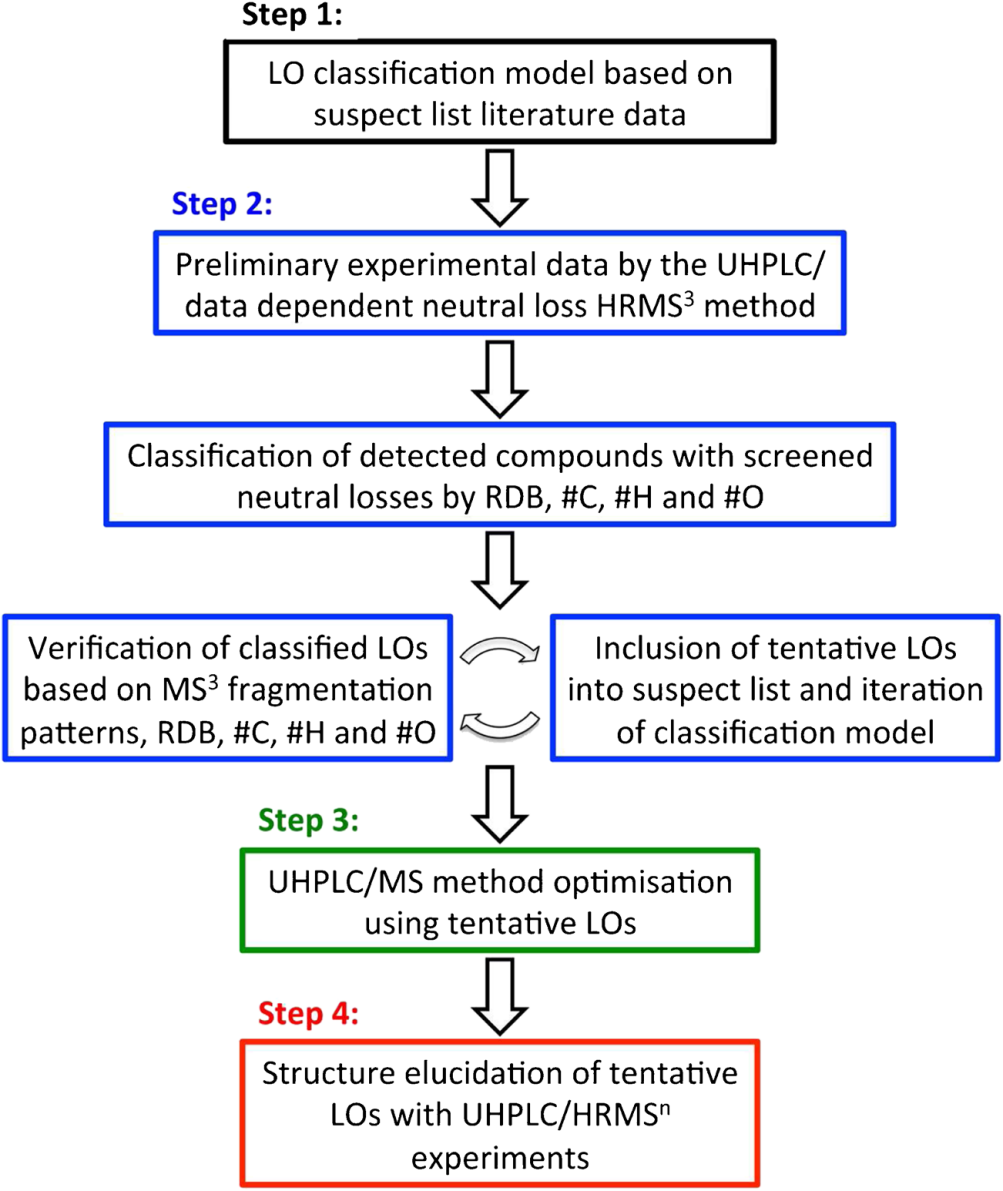
Non-targeted analysis strategy. LO lignin oligomer; RDB ring double bound equivalent, #C number of carbon atoms, #H number of hydrogen atoms, #O number of oxygen atoms

A schematic overview of the developed non-targeted analysis strategy is shown in Fig. 2. In this paragraph, an overview of the strategy is given with references to the following paragraphs for more detailed information. Initially (Step 1, Fig. 2), a suspect list based on previously identified oligomers from literature data was created. Next, a PCA-QDA classification model for lignin dimers and trimers was made based on this data. Then (Step 2, Fig. 2), a Kraft lignin sample was analysed using an UHPLC/HR data-dependent neutral loss MS^3^ method. Compounds with ≥ 1 characteristic neutral losses for LOs were classified using the PCA-QDA classification model. Determined chemical formulas, RDB values based on exact mass measurements and MS^3^ fragmentation data were used for verification. Classifications were rejected if the chemical formula included sulphur, if the RDB values were outside a reasonable range for the type of oligomer (dimers, RDB ≥ 8; trimers, RDB ≥ 12; tetramers, RDB ≥ 16) or if measured and theoretical masses of detected fragments were greater than ± 2 mDa. All tentatively verified LOs were included in the suspect list to improve the classification method. Finally, the updated suspect list was used to create a refined PCA-QDA classification model. The refined model was then used to classify all previously unclassified compounds showing characteristic LO neutral losses. This procedure was performed until no more compounds were classified.

All tentative LOs in the Kraft lignin sample were used as responses in the optimisation of the LC/MS method. In this way, the lack of available reference standards of LOs is compensated for. The LC method optimisation (Step 3, Fig. 2) started with UHPLC column screening followed by a gradient optimisation using the column showing the best chromatographic resolution and analysis time. Then, a multivariate approach was used for the optimisation of the electrospray ionisation source settings. Finally (Step 4, Fig. 2), a structure elucidation of all tentative LOs was done using multiple-stage tandem mass spectrometry.

### Step 1: suspect list and classification model

To create a LO classification model, a suspect list based on literature [14–18, 20] was prepared. The suspect list included 90 LOs identified in different VRL samples (see Electronic Supplementary Material (ESM) Table S1). Only complete proposed LOs and no lignin-carbohydrate complexes were included into the suspect list. The suspect list included the exact mass, the chemical formula, the number of carbon atoms (#C), the number of hydrogen atoms (#H), the number of oxygen atoms (#O), the RDB, the type of LO, a compound label and a literature reference for each LO. The RDB equivalents, #C, #H and #O, were used to perform principal component analysis (PCA). The dataset was pre-processed using autoscaling (mean centering, scaling to unit variance). Based on the PCA, a classification model using quadratic discriminant analysis (QDA) was created for lignin-dimers and lignin-trimers, respectively. For higher molecular weight LOs, no classification model could be created due to the lack of literature data. For the classification, two classes were defined. For example, for the lignin-dimer classification model, class one was defined as “dimer” and class two as “no dimer”. The validation of the model was done using Venetian blinds cross validation with ten cross validation groups.

### Step 2: preliminary experimental data by UHPLC/HR data-dependent neutral loss MS^3^ method

The settings of the preliminary UHPLC/HR data-dependent neutral loss MS^3^ method were based on common settings used for LO identification in literature [14, 15, 19, 20] and on recommended settings by the instrument manufacturer. Five microlitres was injected and the syringe, injection needle, both inside and outside, and injection transfer tubes were flushed after each injection with 4 mL of a flush solution (acetonitrile/water (50/50; *v*/*v*)). Separation was performed on a BEH C18 column fitted with a BEH C18 pre-column. Gradient elution was conducted using water containing 10 mM ammonium formate (solvent A) and acetonitrile/water (95/5; *v*/*v*) containing 10 mM ammonium formate (solvent B). A linear gradient (Gradient 1) starting with 30% B at 0 min to 70% B in 67 min was applied. The flow rate was 250 μL/min and the column temperature was 50 °C. After each run, the column was washed for 30 min with 100% B and 30 min with the starting conditions.

The photo diode array (PDA) detector collected spectral data from 200 to 600 nm in 1 nm steps, using a sample rate of 20 Hz and a filter bandwidth of 9 nm. UV spectra at three different wavelengths (214 nm, 254 nm and 320 nm) were acquired using a sample rate of 20 Hz and a filter bandwidth of 9 nm.

Electrospray ionisation was performed in negative mode using a spray voltage of 3.0 kV, a source heater temperature of 275 °C, a sheath gas flow rate of 60 (arbitrary units), an auxiliary gas flow rate of 30 (arbitrary units), a sweep gas flow rate of 0 (arbitrary units), a capillary temperature of 275 °C, an S-lens RF level of 54.2% and a source fragmentation voltage of 0 V. The ion optics were optimised with direct infusion of a 1 mg/mL solution of the lignin-dimer model compound guaiacylglycerol-beta-guaiacyl ether in acetonitrile/water (50/50; *v*/*v*) using the automatic ion optic optimisation function.

The mass spectrometer was used in data-dependent neutral loss MS^3^ mode. Each scan level was acquired using a resolution of 30,000. The m/z range was set to m/z 120 to 1200. Collision induced dissociation (CID) was performed using a default charge state of 2, an isolation width of m/z 2.0, a normalised dissociation energy of 35.0%, an activation q of 0.250 (arbitrary units) and an activation time of 10 ms. Six different neutral losses were screened, each in a separate run. In every scan event, the top five ions were checked for the applied neutral loss. The screened neutral losses included methyl radical (CH_3_, exact mass 15.0235 Da), water (H_2_O, exact mass 18.0106 Da), formaldehyde (CH_2_O, exact mass 30.0106 Da), carbon dioxide (CO_2_, exact mass 43.9898 Da), formic acid (CH_2_O_2_, exact mass 46.0055 Da) and formaldehyde plus water (CH_2_O + H_2_O, exact mass 48.0211 Da).

The sample was also analysed in full scan mode using the same settings but with a resolution of 100,000, to yield more exact masses, allowing for more accurate determination of chemical formulas and RDB equivalents. The MS system was calibrated every second day using an external calibration standard. Chemical formulas, including C, H, O and S, and the RDB equivalents were determined using Xcalibur. Only tentative chemical formulas with a difference lower than ± 2 mDa between the measured and theoretical mass were considered.

### Step 3: method optimisation using tentative LOs

#### UHPLC column screening

Besides the BEH C18 column, two other columns of different selectivity but of same dimensions were screened for the separation of LOs: BEH Phenyl column and CSH Phenyl-Hexyl column. In front of each column, a corresponding pre-column (BEH Phenyl pre-column and CSH Phenyl-Hexyl pre-column) was placed for protection of the analytical columns. Every column was run under the conditions of the previously described LC/MS method.

The 26 tentative LO dimers, 8 tentative LO trimers and 2 tentative LO tetramers (36 LOs in total) identified using the classification model in combination with neutral loss MS^3^ were used to compare column selectivities. The LTQ Orbitrap was operated in full scan mode with a MS resolution of 100,000, with all other parameters set as in the previously described LC/MS method.

#### UHPLC gradient optimisation

The BEH Phenyl column showed the best selectivity for the selected LOs and was therefore chosen for gradient optimisation. Except for the change of the gradient and the column, all chromatographic, PDA and ESI-MS parameters were kept as in the previously described LC/MS method. Besides Gradient 1, two other gradients were tested to improve the separation of the LOs. Gradient 2 started with 30% B, was then hold up to 5 min, then ramped to 50% B until 25 min, then ramped up to 100% B until 30 min and then hold at 100% until 40 min. Gradient 3 started with 30% B, then hold until 15 min, then ramped up to 40% B until 25 min, then ramped up to 100% B until 35 min and then hold at 100% B until 40 min.

#### Optimisation of the electrospray ionisation efficiency

A full factorial design (2^3^ + 3) was used to screen for variables significantly influencing electrospray ionisation efficiency (ESM Table S2). The electrospray ionisation efficiency was optimised for the 36 tentative LOs identified using the classification model in combination with the preliminary data-dependent neutral loss MS^3^ method. As responses, the peak intensity of each tentative LO in the corresponding extracted ion chromatograms (XIC) was used. Three quantitative variables were investigated: the ESI capillary voltage, the ESI sheath gas flow rate and the ESI auxiliary gas flow rate. The ESI capillary voltage was investigated between 2.0 and 3.0 kV, the ESI sheath gas flow rate from 50 to 70 AU and the ESI auxiliary gas flow rate from 20 to 40 AU. Experiments were performed in a randomised order and partial least squares regression (PLS) was used to evaluate the design. Insignificant variable interactions were stepwise removed to optimise the model. For all experiments, the BEH Phenyl column, a flow rate of 250 μL/min, a column temperature of 50 °C, an injection volume of 5 μL and Gradient 3 were used. The ESI-MS settings were set for all experiments to negative ionisation mode, an ESI source heater temperature of 275 °C, an ESI capillary temperature of 275 °C and a scan range of m/z 120–1200. All experiments were performed in full scan mode using a MS resolution of 100,000.

A second full factorial design (2^2^ + 3) was performed to optimise the ESI sheath gas flow rate and the ESI auxiliary gas flow rate using peak intensities as responses (ESM Table S3). The ESI sheath gas flow rate was varied between 70 and 80 AU and the ESI auxiliary gas flow rate from 10 to 20 AU. All experiments were performed in a randomised order and the same LC/MS settings as in the first DOE. The capillary voltage was set to 3.0 kV. For the evaluation of the design, PLS was applied.

### Step 4: structure elucidation of tentative LOs using UHPLC/HRMS^n^

The final LC/MS method that was used for the structure elucidation of the tentative LOs included the BEH Phenyl column and Gradient 3 at a flow rate of 250 μL/min, a column temperature of 50 °C and an injection volume of 5 μL. For all experiments, ESI was applied in negative ionisation mode with a source heater temperature of 275 °C, an ESI capillary temperature of 275 °C, an ESI capillary voltage of 3.0 kV, a sheath gas flow rate of 80 AU, an auxiliary gas flow rate of 20 AU and a scan range of m/z 120–1200. To avoid a low duty cycle in the mass spectrometric detection, the MS resolution for each MS stage was set to 15,000.

## Results and discussions

### First classification model based on literature data

**Fig. 3:**
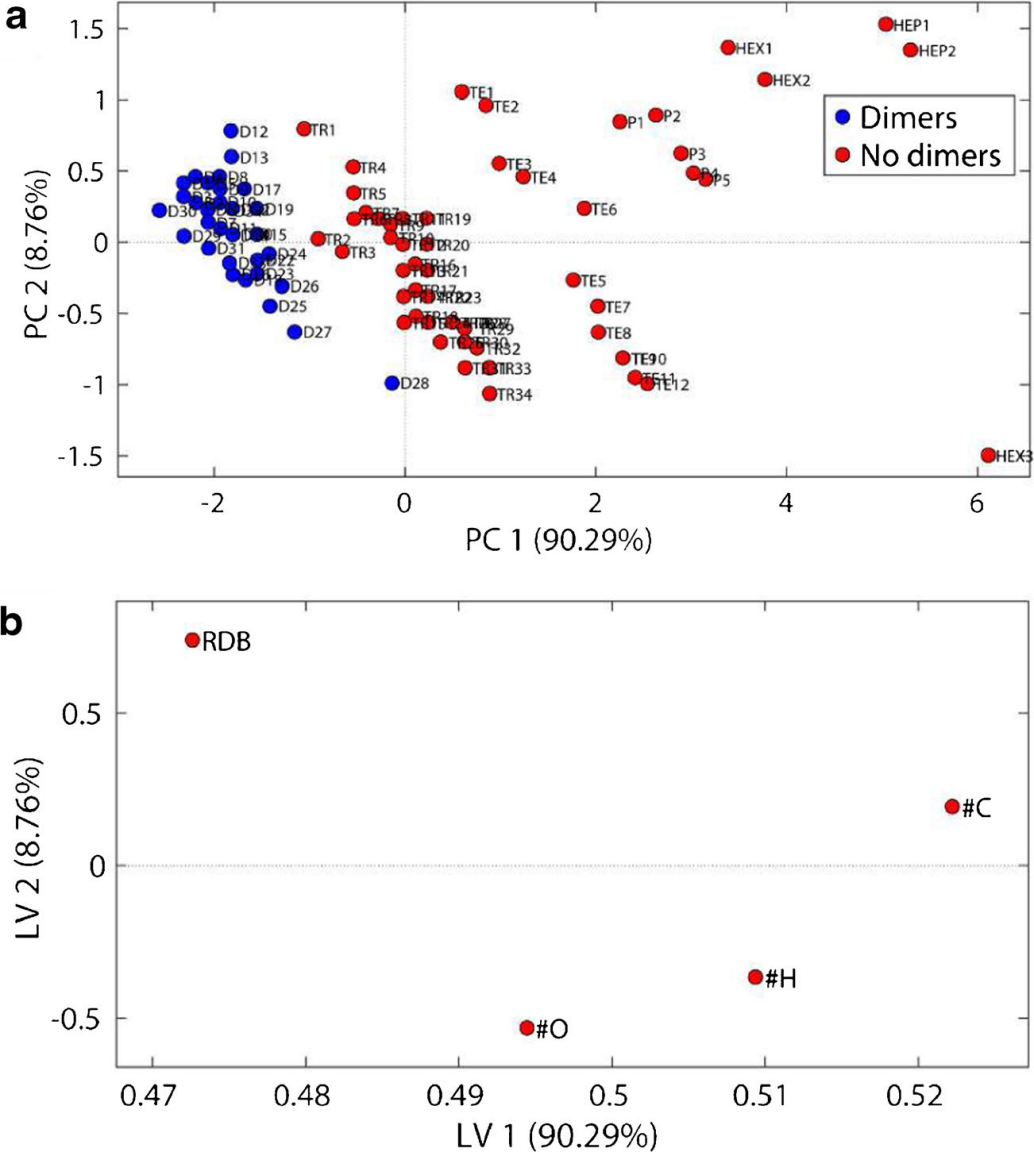
Scores plot (**a**) and loading plot (**b**) of the PCA model used for dimer classification. The PCA model is calculated on the literature-based suspect list. D dimers, TR trimers, TE tetramers, P pentamers, HEX hexamers, HEP heptamers, RDB ring double bound equivalent, #C number of carbon atoms, #H number of hydrogen atoms, #O number of oxygen atoms

An initial PCA-QDA classification model for dimers and trimers was created based on a suspect list containing 90 LOs found in literature (Fig. 3). The dimer classification model required three principal components and yielded an error rate of 0.01, a cross validation error rate of 0.01 and an accuracy of 0.99. Out of the 34 dimers in the suspect list, 33 were predicted as dimers and one (D28, Fig. 3a) was predicted as a no dimer. Several clusters of lignin oligomers can be observed in the scores plot (Fig. 3a). The loading plot shows that the RDB equivalent and #C strongly influences latent variable (LV) 1, whereas RDB and #O influences on LV2 (Fig. 3b). This shows that the data separation on PC 1 is mainly dominated by the difference of RDB equivalents and #C and on PC 2 by the RDB equivalents and #O. For example, the influence of the #C and the #O can be seen in the scores plot (Fig. 3a), where higher order LOs, pentamers or hexamers, have higher values on PC 1 due to a higher #C and #O compared to lower order LOs, like dimers or trimers. The trimer classification model required three principal components and yielded an error rate of 0.01, a cross validation error rate of 0.03 and an accuracy of 0.99. Out of the 34 trimers in the suspect list, 33 were predicted as a trimer and one (TR1, Fig. 3a) was predicted as a no trimer. The score plot of the trimer classification model is shown in ESM Fig. S1.

### Preliminary experimental data by UHPLC/HR data-dependent neutral loss MS^3^

**Fig. 4:**
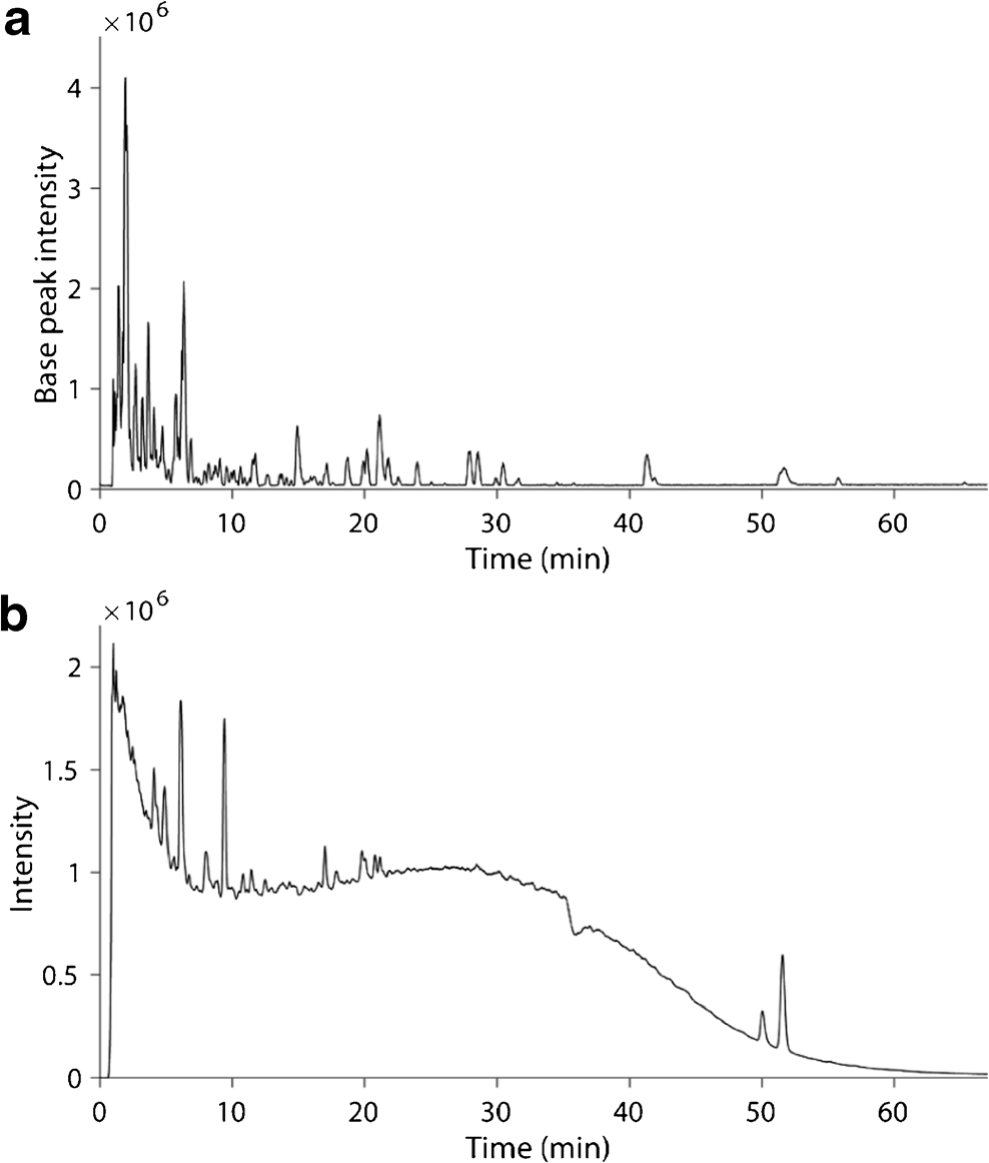
Base peak ion chromatogram (**a**) and UV-chromatogram at 254 nm (**b**) generated from Kraft lignin using the preliminary LC/MS method

Most compounds that could be ionised in the Kraft lignin sample eluted during the first 25 min (Fig. 4a). Between 25 and 67 min, only a few compounds could be detected. However, it is likely that a large number of compounds do not ionise, as suggested by the UV-chromatogram (Fig. 4b), which shows that UV active compounds elute until almost 60 min, with a large majority eluting already within 35 min. The UV-spectrum clearly illustrates the complexity of the Kraft lignin sample. From the MS data, 587 individual peaks could be resolved (ESM Table S4). Out of these, 99 peaks were associated with ≥ 1 characteristic neutral loss. The most common neutral loss was loss of a methyl radical (62 peaks), followed by loss of water (36), carbon dioxide (35), formaldehyde (27), formic acid (18) and the combination of formaldehyde and water (12). Four neutral losses were observed for 4 peaks, three in 19 peaks, two in 41 peaks and one in 35 peaks.

### Classification and verification of tentative lignin oligomers

**Fig. 5:**
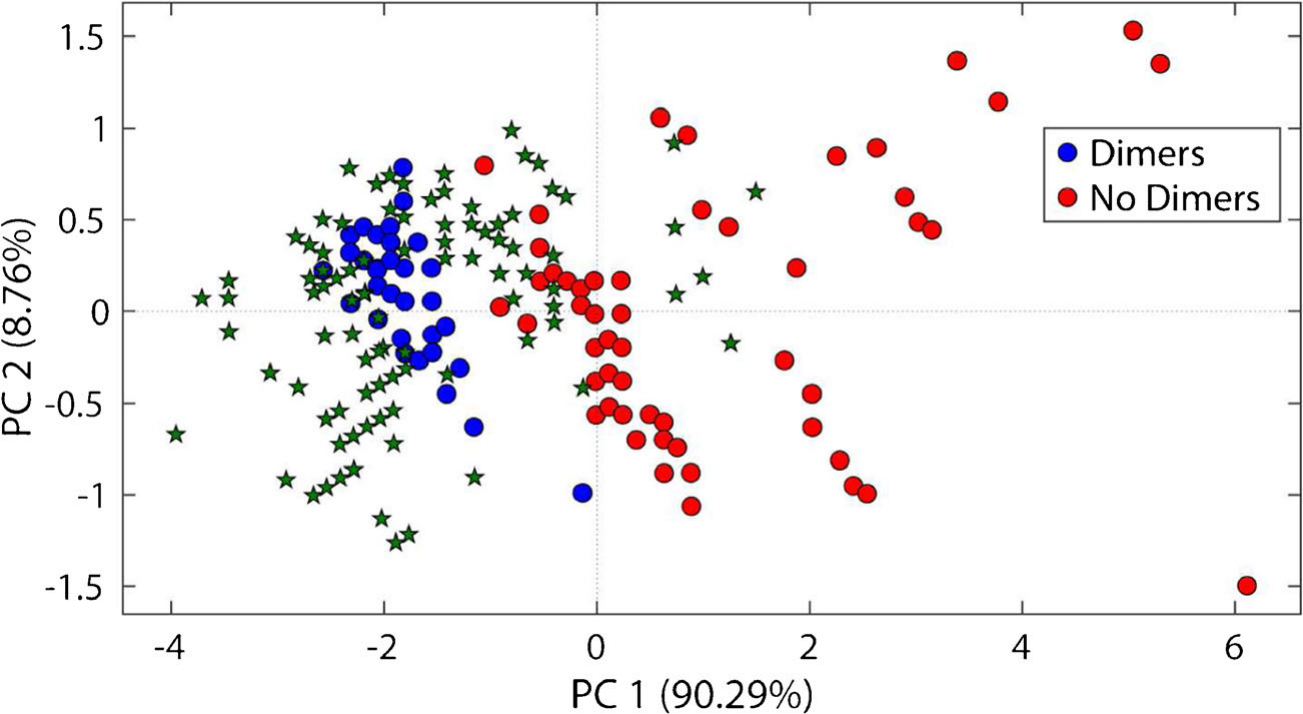
Compounds with ≥ 1 LO characteristic neutral loss (99 green stars) detected in Kraft lignin using the preliminary LC/MS method and projected in the PCA model used for dimer classification

From the 99 detected compounds with ≥ 1 characteristic neutral loss, 34 compounds were classified as dimer or trimer (Table 1). The projected position of the 99 detected compounds with ≥ 1 characteristic neutral loss in the PCA can be seen in Fig. 5. The classification model was repeated two times with the expanded suspect list until no more compounds were classified. Two compounds showed a difference of more than 2 mDa between measured and theoretical mass and were consequently excluded from the list. The accurate mass (± 2 mDa) determined chemical formula and RDB equivalents of four of the compounds matched with masses from the suspect list (LOs 10, 19, 32, 33).

**Table 1:** List of tentative lignin oligomers detected using the preliminary LC/MS method and classified using the PCA-QDA model. RDB, ring double bond equivalent; RT_PM_, retention time in preliminary LC/MS method

Compound label	Measured m/z	Chemical formula	RDB	RT_PM_ (min)	Neutral losses (Da)	Classified type of oligomer
1	243.0664	C_14_H_11_O_4_	9.5	2.86	15, 18, 48	Dimer
2	245.0819	C_14_H_14_O_4_	8.5	3.19	15	Dimer
3	257.0819	C_15_H_13_O_4_	9.5	4.83	15	Dimer
4	259.0971	C_15_H_15_O_4_	8.5	3.61	15, 30	Dimer
5	269.0816	C_16_H_13_O_4_	10.5	5.47	15	Dimer
6	271.0975	C_16_H_15_O_4_	9.5	4.41	15	Dimer
7	273.0768	C_15_H_13_O_5_	9.5	3.17	15, 44, 48	Dimer
8	273.1128	C_16_H_15_O_4_	8.5	5.84	30	Dimer
9	287.0926	C_16_H_15_O_5_	9.5	3.50	15, 44	Dimer
10	299.0918	C_17_H_15_O_5_	10.5	6.20	15, 30	Dimer^1^
11	301.0717	C_16_H_13_O_6_	10.5	1.64	15, 44, 48	Dimer
12	313.1079	C_18_H_17_O_5_	10.5	6.77	15, 18, 30	Dimer
13	315.1236	C_18_H_19_O_5_	9.5	2.59	15, 44, 30	Dimer
14	321.1131	C_20_H_17_O_4_	12.5	8.15	15	Dimer
15	335.0922	C_20_H_15_O_5_	13.5	9.48	15, 44	Dimer
16	349.1077	C_21_H_17_O_5_	13.5	9.68	15, 44	Dimer
17	351.1235	C_21_H_19_O_5_	12.5	9.42	15	Dimer
18	353.1391	C_21_H_21_O_5_	11.5	6.65	15	Dimer
19	357.1339	C_20_H_21_O_6_	10.5	4.33	15, 44	Dimer^1^
20	361.1652	C_20_H_25_O_6_	8.5	1.99	15, 48	Dimer
21	373.1288	C_20_H_21_O_7_	10.5	2.67	18, 44, 46	Dimer
22	377.1387	C_23_H_21_O_5_	13.5	12.58	15, 30	Dimer/trimer^2^
23	393.1332	C_23_H_21_O_6_	13.5	13.59	15, 18, 30, 48	Dimer/trimer^2^
24	395.1132	C_22_H_19_O_7_	13.5	1.15	44	Dimer
25	395.1494	C_23_H_23_O_6_	12.5	7.42	15	Dimer/trimer^2^
26	419.1495	C_25_H_23_O_6_	14.5	14.98	15, 30	Trimer
27	421.1289	C_24_H_21_O_7_	14.5	6.06	15, 30, 48	Dimer/trimer^2^
28	451.1756	C_26_H_27_O_7_	13.5	11.15	15, 46	Trimer
29	463.1756	C_27_H_27_O_7_	14.5	13.93	15, 30	Trimer
30	479.1711	C_27_H_27_O_8_	14.5	5.05	15, 30, 46	Trimer
31	483.2010	C_27_H_31_O_8_	12.5	4.36	46	Trimer
32	491.1704	C_28_H_27_O_8_	15.5	8.90	15, 44	Trimer^1^
33	507.1649	C_28_H_27_O_9_	15.5	3.69	15, 30	Trimer^1^
34	509.2166	C_29_H_33_O_8_	13.5	5.64	15, 30, 46	Trimer
35	627.2230	C_36_H_35_O_10_	19.5	13.44	15, 30, 48	Tetramer^3^
36	631.2539	C_36_H_39_O_10_	17.5	8.09	15, 30	Tetramer^3^

^1^Hit on suspect list by accurate mass, determined chemical formula and RDB equivalent

^2^Classified as dimer, but determined chemical formula, RDB and MS^3^ fragmentation are more reasonable for a trimer

^3^Not classified by the classification model, but rather classified by determined chemical formula, RDB and MS^3^ fragmentation

Figure 5 shows that most of the 99 detected compounds with ≥ 1 characteristic neutral loss are projected close to the dimer and trimer cluster. A significant number of compounds are projected at lower PC 1 and PC 2 values. In this area, compounds with low RDB values and low numbers of carbon, hydrogen and oxygen atoms are projected. These compounds are likely to be lignin monomers, showing similar neutral losses compared to lignin oligomers [25]. Six of the compounds are projected close to the tetramer cluster, and therefore might be tetramers.

Classified compounds were verified using the determined chemical formula, the RDB equivalent and the MS^3^ fragmentation pattern. The observed MS^2^ and MS^3^ fragments for all verified LOs are shown in ESM Tables S5 to S40. Several false positives that showed for example too few oxygen atoms, included sulphur or showed too low RDB equivalents were excluded. Four compounds classified as dimers (LOs 22, 23, 25, 27) are likely to be trimers according to their determined chemical formulas, RDB equivalents and MS^3^ fragmentation pathways. Figure 5 shows that some of the compounds are projected in the PCA close to tetramers from literature. Two out of six compounds projected close to the tetramer cluster (LOs 35, 36) were identified as tentative tetramers based on determined chemical formulas, RDB equivalents and MS^3^ fragmentation pathways.

Several combinations of neutral losses, in addition to the characteristic neutral losses reported in literature, are possible. The loss of formaldehyde (30.0106 Da) observed for example for the LO 20 (loss of 30.0105 Da, see ESM Table S24) differs only by 36.4 mDa compared to the loss of two methyl radicals (30.0470 Da), which is observed for example for LO 6 (loss of 30.0472 Da, see ESM Table S10). Consecutive losses of formaldehyde and two methyl radicals are also possible, as observed for LO 29 (loss of 30.0110 in MS^2^; loss of 30.0469 in MS^3^, see ESM Table S33). Loss of formaldehyde in combination with one water molecule (48.0211 Da) is also typical for LOs, as observed for example for LO 20 (loss of 48.0211 Da, see ESM Table S24). Moreover, also, a combination of two methyl radicals and one water molecule (48.0575 Da) is possible for LOs as for example observed for LO 27 (loss of 48.0579 Da, see ESM Table S31). Yet, another possible neutral loss combination involves the loss one methyl radical and one CHO radical (44.0262 Da), which is observed for example for LO 7 (loss of 44.0264 Da, see ESM Table S11). This neutral loss is very close to the common LO neutral loss of CO_2_ (43.9898 Da). The identified neutral losses with masses close to the characteristic neutral losses reported in literature show clearly the advantage of using high MS resolution in all MS stages.

### UHPLC method optimisation

The performance of the screened UHPLC columns was compared using a resolution level graph (ESM Fig. S2). All columns show similar chromatographic resolution (*R*_S_) for the tentative LOs. A comparison of the retention factors (ESM Fig. S3) shows that the shortest analysis time was achieved with the BEH Phenyl column, and this column was therefore chosen for further method optimisation. Finally, Gradient 3 was chosen based on an improved peak resolution, as compared to the other two tested gradients (ESM Fig. S4).

### Optimisation of electrospray ionisation efficiency

**Fig. 6:**
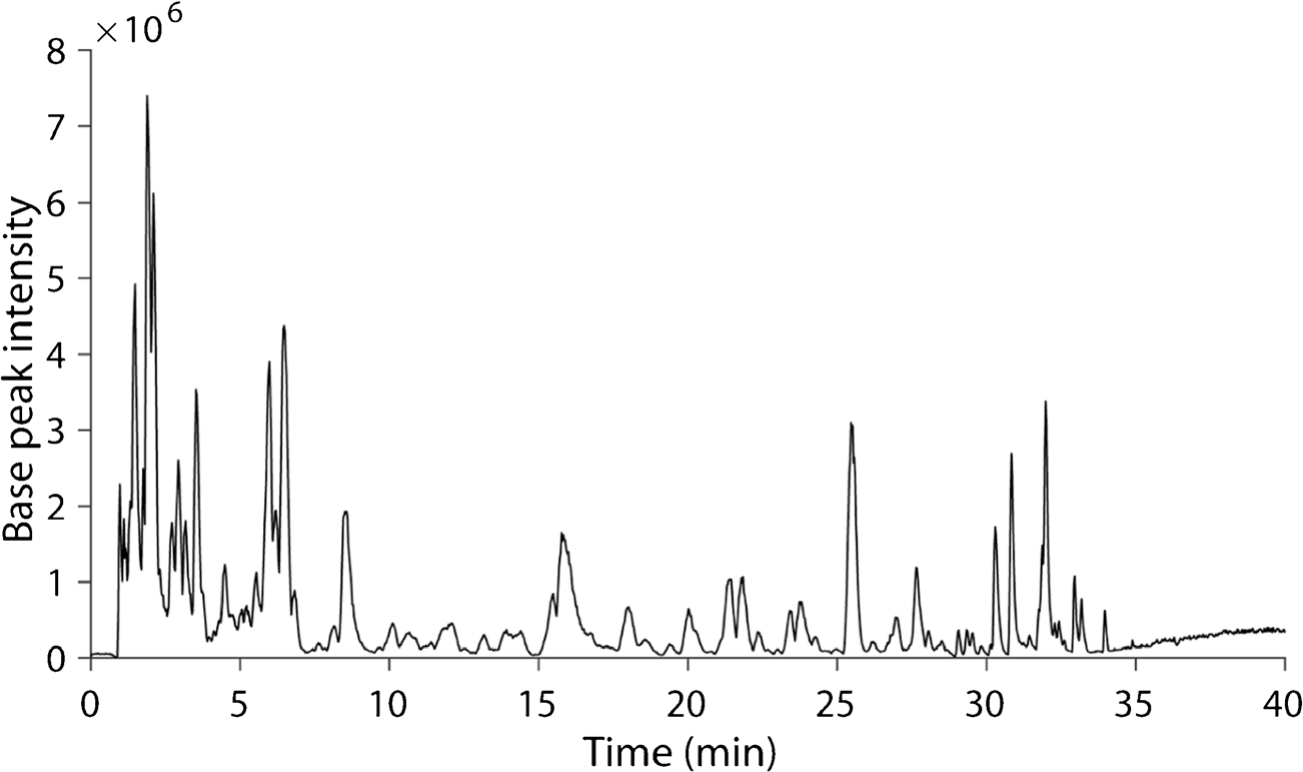
Base peak ion chromatogram of the Kraft lignin sample obtained using the optimised LC/MS method. BEH Phenyl column; Gradient 3; flow rate, 250 μL/min; column temperature, 50 °C; injection volume, 5 μL; ESI in negative ionisation mode; source heater temperature, 275 °C; capillary temperature, 275 °C; capillary voltage, 3.0 kV; sheath gas flow rate, 80 AU; auxiliary gas flow rate, 20 AU; scan range, m/z 120–1200

First, the influence of the capillary voltage, the sheath gas flow rate and the auxiliary gas flow rate on the peak intensities was investigated using a factorial design. The obtained explained variance (*R*^2^), the cross-validated predictability, the model validity and the reproducibility of the obtained models for each LO are shown in ESM Table S41. Models could be fitted for 29 out of the 36 investigated LOs. The obtained coefficient plots are shown in ESM Fig. S5. Models for seven LOs (LOs 1, 2, 4, 7, 8, 11 and 21) showed a significant lack of fit (method validity < 0.25). All 29 acceptable models showed a positive influence of the capillary voltage on the peak intensity, 25 models showed a positive influence on the sheath gas flow rate and 23 a negative influence of the auxiliary gas flow. Furthermore, interactions between capillary voltage and auxiliary gas flow rate and between sheath gas flow rate and auxiliary gas flow rate had a negative influence on the response. Each interaction was observed in 15 models. Based on these results, a second factorial design was performed to further optimise the ionisation efficiency. As the capillary voltage could not be set higher than 3.0 kV due to arcing, only the influence of the sheath gas flow rate and the auxiliary gas flow rate was investigated in more detail. The sheath gas flow rate was investigated in a higher range and the auxiliary gas flow rate was investigated in a lower range, as compared to the initial design. ESM Table S42 shows the obtained explained variance (*R*^2^), the cross-validated predictability, the model validity and the reproducibility of the obtained models for each LO in the second design. The obtained coefficient plots are shown in ESM Fig. S6. Models could be fitted for 31 out of the 36 investigated LOs and models for five LOs showed a significant lack of fit (LOs 15, 25, 27, 30 and 36). A positive influence of the sheath gas flow rate was observed for 31 LOs and a negative influence of the auxiliary gas flow rate for 18 LOs. A significant positive interaction was observed between the sheath gas flow rate and the auxiliary gas flow rate for 27 LOs. The highest peak intensities were hence observed with all factors at their highest level (sheath gas flow rate at 80 AU and auxiliary gas flow rate at 20 AU). Intensities for 25 out of the 36 LOs were significantly improved (*p* < 0.05, heteroscedastic *t* test), with only LO 15 showing a lower peak intensity (*p* < 0.05), as compared to the preliminary LC/MS method (ESM Table S43). A base peak ion chromatogram of the Kraft lignin sample using the optimised LC/MS method is shown in Fig. 6.

### Structure elucidation of lignin oligomers

**Fig. 7:**
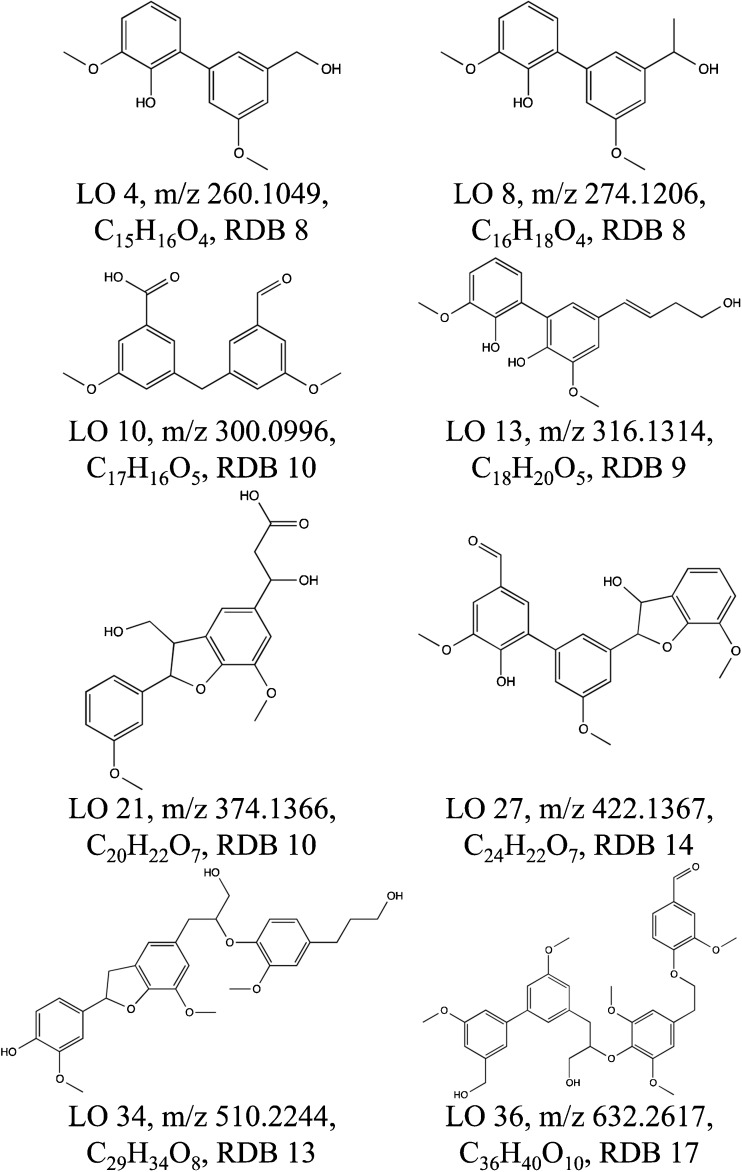
Tentative structures of eight lignin oligomers found in the Kraft lignin sample. RDB ring double bond equivalent

**Fig. 8:**
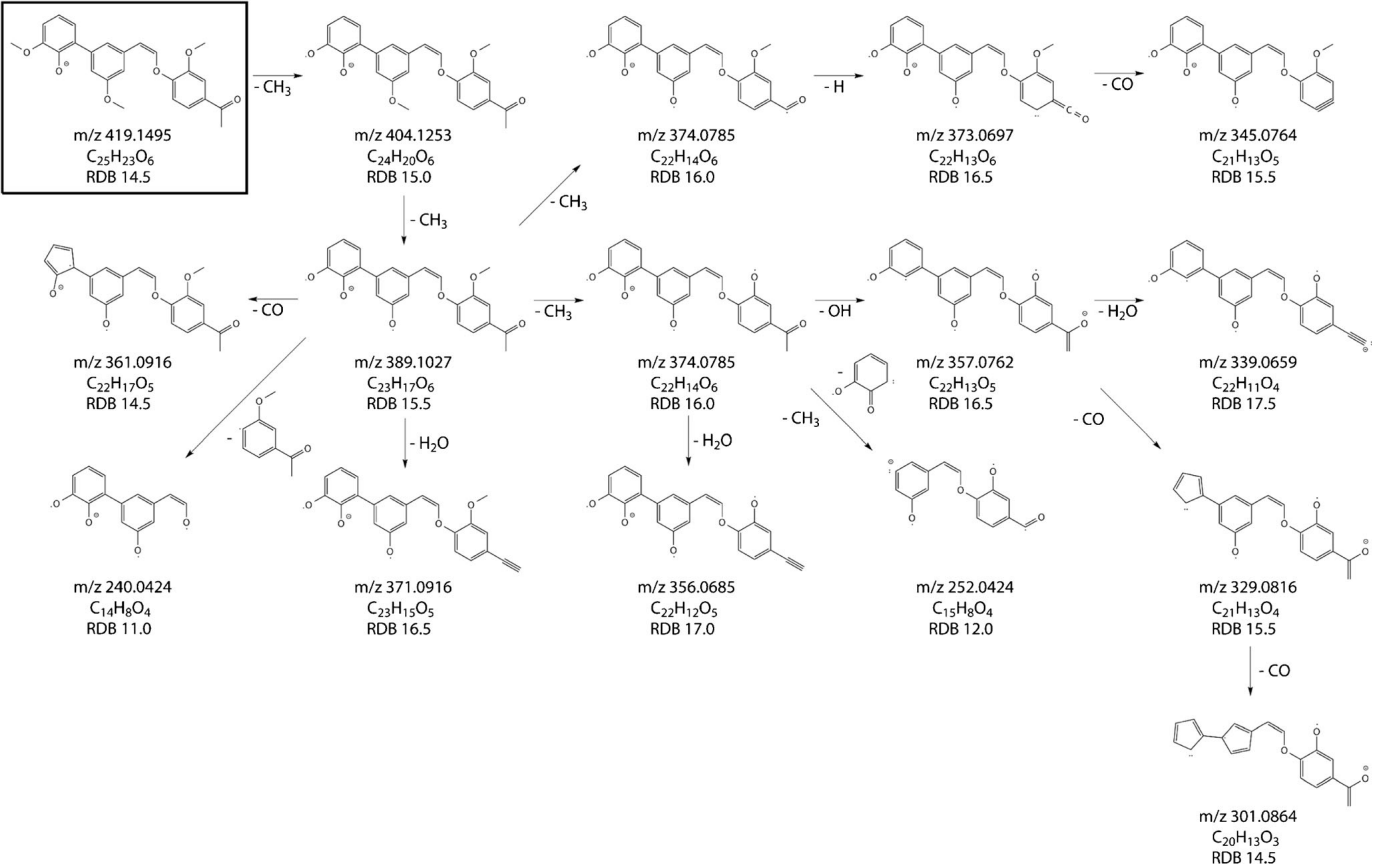
Proposed MS^7^ fragmentation pathway for the tentative lignin oligomer m/z 419.1495 (LO 29). For details about the detected ions, see Table S29. RDB ring double bond equivalent

Based on LC/MS^n^ experiments, tentative structures are proposed for the 36 tentative LOs. A selection of proposed structures is shown in Fig. 7. The other proposed structures can be seen in ESM Fig. S7. The detected MS^n^ fragments for all identified LOs can be seen in ESM Tables S5 to S40. MS^4^ could be acquired for 12 LOs, MS^5^ for 15 LOs, MS^6^ for 4 LOs and MS^7^ for one LO. For 4 LOs, spectra could only be recorded until MS^3^. The proposed fragmentation pathways as outlined for LO 26 (Fig. 8) clearly illustrate the capability of LC/MS^n^ measurements to provide valuable structural information on LOs. The confidence in the proposed chemical structure will increase with the number of MS stages and observed fragments. Importantly, a high resolution in all MS stages provides reliable determination of the chemical formulas of the fragments and the neutral losses, and also ring double bond equivalents for each detected fragment, and hence a high confidence in the determination of LO structures.

## Conclusions

Non-targeted analysis of LOs using UHPLC/HR data-dependent neutral loss MS^3^ experiments in combination with PCA-QDA classification is a powerful tool for identification of tentative LOs in complex lignin samples. With the developed method, 36 tentative LOs were identified out of 587 detected peaks in the Kraft lignin sample. The 36 identified LOs included lignin dimers, trimers and tetramers. The multivariate classification approach does not apply mass cut offs, which is beneficial since mass cut offs will lead to biased classification results. Furthermore, with the combination of neutral loss scans and classification model, a new non-targeted analysis identification confidence level has been introduced, which might be also applicable to other types of compound classes and complex samples. A systematic method optimisation significantly improved the ionisation efficiency for 25 out of 36 LOs allowing LC/MS^n^ experiments up to LC/MS^7^ to be performed. High resolution at all MS^n^ stages improves the structure elucidation of LOs in complex lignin samples, without the need for chemical standards.

## Electronic supplementary material

ESM 1 (PDF 3302 kb)
