# Supplementary material for: Identification of lignin oligomers in Kraft lignin using ultra-high-performance liquid chromatography/high-resolution multiple-stage tandem mass spectrometry (UHPLC/HRMSn)
Source: Anal Bioanal Chem. 2018 Oct 10;410(29):7803–14. doi: 10.1007/s00216-018-1400-4 (PMC6244760; doi:10.1007/s00216-018-1400-4)
Supplement: Supplementary file 1 — (PDF 3302 kb) [file 216_2018_1400_MOESM1_ESM.pdf]

Analytical and Bioanalytical Chemistry

Electronic Supplementary Material

Identification of lignin oligomers in Kraft lignin using ultra-high-performance liquid chromatography/high-resolution multiple stage tandem mass spectrometry (UHPLC/HRMS^n)

Jens Prothmann, Peter Spégel, Margareta Sandahl, Charlotta Turner

Contents:

Table S1. Suspect list including lignin oligomers identified in literature and in the Kraft lignin sample.

Table S2. Experiments performed in the first DOE.

Table S3. Experiments performed in the second DOE.

Figure S1. Scores plot from the trimer classification model.

Table S4. MZmine workflow and settings for peak identification in the Kraft lignin sample.

Tables S5–S40. Detected MS^n fragments for LO 1 to LO 36

Figure S2. Resolution level graph from the column screening.

Figure S3. Retention factor graph from the column screening.

Figure S4. Resolution level graph from the gradient optimisation.

Table S41. Explained variance, cross-validated predictability, model validity and reproducibility of the full factorial design (2^3+3) used to investigate the influence of the capillary voltage, the sheath gas flow rate and the auxiliary gas flow rate on the base peak intensity of the identified lignin oligomers.

Figure S5. Normalized coefficient plots of the identified LOs from the first design of experiment for the optimization of the MS ionization efficiency

Table S42. Explained variance, cross-validated predictability, model validity and reproducibility of the full factorial design (2^2+3) used to examine the impact of the sheath gas flow rate and the auxiliary gas flow rate on the base peak intensity of the identified lignin oligomers.

Figure S6. Normalized coefficient plots of the identified LOs from the second design of experiment for the optimization of the MS ionization efficiency

Table S43. Average base peak intensities and standard deviations of the identified LOs in the Kraft lignin samples using the preliminary LC/MS method and the optimised LC/MS method. Averages calculated from n=3. Data are compared using the heteroscedastic t-test.

Figure S7. Tentative structures of identified lignin oligomers in the Kraft lignin sample.

Table S1 Suspect list including lignin oligomers identified in literature and in the Kraft lignin sample. #C: number of carbon atoms, #H: number of hydrogen atoms, #O: number of oxygen atoms, RDB: ring double bond equivalent, LO: lignin oligomer, KL: Kraft lignin, *identified in first classification model run, **identified in second classification model run, ***identified in third classification run

Exact mass (neutral compound)	$[M-H]^-$	$[M+H]^+$	Chemical formula (neutral compound)	#C	#H	#O	RDB	Type of LO	Compound label	Reference
244.0742	243.0664	245.0820	C14 H12 O4	14	12	4	9.0	Dimer	D35	Found in KL*
246.0897	245.0819	247.0975	C14 H18 O4	14	18	4	8.0	Dimer	D36	Found in KL*
258.0897	257.0819	259.0975	C15 H14 O4	15	14	4	9.0	Dimer	D37	Found in KL*
260.1049	259.0971	261.1127	C15 H16 O4	15	16	4	8.0	Dimer	D38	Found in KL*
270.0894	269.0816	271.0972	C16 H14 O4	16	14	4	10.0	Dimer	D39	Found in KL*
272.1053	271.0975	273.1131	C16 H16 O4	16	16	4	9.0	Dimer	D40	Found in KL*
274.0846	273.0768	275.0924	C15 H14 O5	15	14	5	9.0	Dimer	D41	Found in KL*
274.0850	273.0772	275.0928	C15 H14 O5	15	14	5	9.0	Dimer	D30	Jarrell et al.
274.1206	273.1128	275.1284	C16 H18 O4	16	18	4	8.0	Dimer	D42	Found in KL*
288.1004	287.0926	289.1082	C16 H16 O5	16	16	5	9.0	Dimer	D43	Found in KL*
298.1205	297.1127	299.1283	C18 H18 O4	18	18	4	10.0	Dimer	D1	Kiyota et al.
298.1205	297.1127	299.1283	C18 H18 O5	18	18	5	10.0	Dimer	D2	Kiyota et al.
300.0997	299.0919	301.1075	C17 H16 O5	17	16	5	10.0	Dimer	D3	Banoub et al.
302.0795	301.0717	303.0873	C16 H14 O6	16	14	6	10.0	Dimer	D44	Found in KL*
304.0955	303.0877	305.1033	C16 H16 O6	16	16	6	9.0	Dimer	D29	Jarrell et al.
312.0997	311.0919	313.1075	C18 H16 O5	18	16	5	11.0	Dimer	D4	Banoub et al.
314.1157	313.1079	315.1235	C18 H18 O5	18	18	5	10.0	Dimer	D45	Found in KL*
316.1314	315.1236	317.1392	C18 H20 O5	18	20	5	9.0	Dimer	D46	Found in KL*
322.1209	321.1131	323.1287	C20 H18 O4	20	18	4	12.0	Dimer	D47	Found in KL*
326.1154	325.1076	327.1232	C19 H18 O5	19	18	5	11.0	Dimer	D5	Banoub et al.
328.1311	327.1233	329.1389	C19 H20 O5	19	20	5	10.0	Dimer	D6	Kiyota et al.
330.0746	329.0668	331.0824	C17 H14 O7	17	14	7	11.0	Dimer	D34	Jarrell et al.
330.1103	329.1025	331.1181	C18 H18 O6	18	18	6	10.0	Dimer	D7	Banoub et al.
332.1266	331.1188	333.1344	C18 H20 O6	18	20	6	9.0	Dimer	D31	Jarrell et al.

336.1000	335.0922	337.1078	C20 H16 O5	20	16	5	13.0	Dimer	D48	Found in KL*
336.1268	335.1190	337.1346	C20 H20 O6	20	20	6	11.0	Dimer	D32	Jarrell et al.
340.0947	339.0869	341.1025	C19 H16 O6	19	16	6	12.0	Dimer	D8	Banoub et al.
340.1310	339.1232	341.1388	C20 H20 O5	20	20	5	11.0	Dimer	D9	Banoub et al.
342.1103	341.1025	343.1181	C19 H18 O6	19	18	6	11.0	Dimer	D10	Banoub et al.
344.1260	343.1182	345.1338	C19 H20 O6	19	20	6	10.0	Dimer	D11	Banoub et al.
350.0791	349.0713	351.0869	C20 H14 O6	20	14	6	14.0	Dimer	D12	Banoub et al.
350.1155	349.1077	351.1233	C21 H18 O5	21	18	5	13.0	Dimer	D49	Found in KL*
352.0947	351.0869	353.1025	C20 H16 O6	20	16	6	13.0	Dimer	D13	Banoub et al.
352.1313	351.1235	353.1391	C21 H20 O5	21	20	5	12.0	Dimer	D50	Found in KL*
354.1469	353.1391	355.1547	C21 H22 O5	21	22	5	11.0	Dimer	D51	Found in KL*
358.1416	357.1338	359.1494	C20 H22 O6	20	22	6	10.0	Dimer	D14	Kiyota et al.
358.1416	357.1338	359.1494	C20 H22 O6	20	22	6	10.0	Dimer	D15	Kiyota et al.
362.1730	361.1652	363.1808	C20 H26 O6	20	26	6	8.0	Dimer	D52	Found in KL*
363.1080	362.1002	364.1158	C18 H19 O8	18	19	8	9.5	Dimer	D16	Banoub et al.
368.1260	367.1182	369.1338	C21 H20 O6	21	20	6	12.0	Dimer	D17	Banoub et al.
372.1217	371.1139	373.1295	C20 H20 O7	20	20	7	9.0	Dimer	D33	Jarrell et al.
376.1522	375.1444	377.1600	C20 H24 O7	20	24	7	9.0	Dimer	D18	Kiyota et al.
378.1465	377.1387	379.1543	C23 H22 O5	23	22	5	13.0	Dimer	D54	Found in KL**
384.1209	383.1131	385.1287	C21 H20 O7	21	20	7	12.0	Dimer	D19	Banoub et al.
386.1366	385.1288	387.1444	C21 H22 O7	21	22	7	11.0	Dimer	D20	Banoub et al.
388.1522	387.1444	389.1600	C21 H24 O7	21	24	7	10.0	Dimer	D21	Kiyota et al.
388.1522	387.1444	389.1600	C21 H24 O7	21	24	7	10.0	Dimer	D22	Kiyota et al.
390.1326	389.1248	391.1404	C20 H22 O8	20	22	8	10.0	Dimer	D23	Huis et al.
394.1410	393.1332	395.1488	C23 H22 O6	23	22	6	13.0	Dimer	D55	Found in KL**
396.1210	395.1132	397.1288	C22 H20 O7	22	20	7	13.0	Dimer	D53	Found in KL*
396.1572	395.1494	397.1650	C23 H24 O6	23	24	6	12.0	Dimer	D56	Found in KL**
402.1314	401.1236	403.1392	C21 H22 O8	21	22	8	11.0	Dimer	D24	Banoub et al.
406.1628	405.1550	407.1706	C21 H26 O8	21	26	8	9.0	Dimer	D25	Kiyota et al.

418.1628	417.1550	419.1706	C22 H26 O8	22	26	8	10.0	Dimer	D26	Kiyota et al.
420.1573	419.1495	421.1651	C25 H24 O6	25	24	6	14.0	Trimer	T35	Found in KL*
422.1367	421.1289	423.1445	C24 H22 O7	24	22	7	14.0	Dimer	D57	Found in KL***
432.1209	431.1131	433.1287	C25 H20 O7	25	20	7	16.0	Trimer	TR1	Banoub et al.
436.1733	435.1655	437.1811	C22 H28 O9	22	28	9	9.0	Dimer	D27	Kiyota et al.
452.1834	451.1756	453.1912	C26 H28 O7	26	28	7	13.0	Trimer	T36	Found in KL*
454.1991	453.1913	455.2069	C26 H30 O7	26	30	7	12.0	Trimer	TR2	Banoub et al.
464.1834	463.1756	465.1912	C27 H28 O7	27	28	7	14.0	Trimer	T37	Found in KL*
480.1789	479.1711	481.1867	C27 H28 O8	27	28	8	14.0	Trimer	T38	Found in KL*
483.2019	482.1941	484.2097	C27 H31 O8	27	31	8	12.5	Trimer	TR3	Banoub et al.
484.2088	483.2010	485.2166	C27 H32 O8	27	32	8	12.0	Trimer	T39	Found in KL*
490.1627	489.1549	491.1705	C28 H26 O8	28	26	8	16.0	Trimer	TR4	Banoub et al.
492.1784	491.1706	493.1862	C28 H28 O8	28	28	8	15.0	Trimer	TR5	Banoub et al.
494.1941	493.1863	495.2019	C28 H30 O8	28	30	8	14.0	Trimer	TR6	Kiyota et al.
508.1733	507.1655	509.1811	C28 H28 O9	28	28	9	15.0	Trimer	TR7	Banoub et al.
510.2244	509.2166	511.2322	C29 H34 O8	29	34	8	13.0	Trimer	T40	Found in KL*
522.1890	521.1812	523.1968	C29 H30 O9	29	30	9	15.0	Trimer	TR8	Banoub et al.
536.2046	535.1968	537.2124	C30 H32 O9	30	32	9	15.0	Trimer	TR9	Kiyota et al.
537.2124	536.2046	538.2202	C30 H33 O9	30	33	9	14.5	Trimer	TR10	Banoub et al.
550.1839	549.1761	551.1917	C30 H30 O10	30	30	10	16.0	Trimer	TR11	Banoub et al.
552.1871	551.1793	553.1949	C26 H32 O13	26	32	13	11.0	Dimer	D28	Huis et al.
552.2014	551.1936	553.2092	C30 H32 O10	30	32	10	15.0	Trimer	TR12	Huis et al.
554.2152	553.2074	555.2230	C30 H34 O10	30	34	10	14.0	Trimer	TR13	Kiyota et al.
556.2324	555.2246	557.2402	C30 H36 O10	30	36	10	13.0	Trimer	TR14	Huis et al.
558.2480	557.2402	559.2558	C30 H38 O10	30	38	10	12.0	Trimer	TR15	Huis et al.
568.1943	567.1865	569.2021	C30 H32 O11	30	32	11	15.0	Trimer	TR16	Banoub et al.
570.2125	569.2047	571.2203	C30 H34 O11	30	34	11	14.0	Trimer	TR17	Huis et al.
572.2258	571.2180	573.2336	C30 H36 O11	30	36	11	13.0	Trimer	TR18	Kiyota et al.
578.1788	577.1710	579.1866	C31 H30 O11	31	30	11	17.0	Trimer	TR19	Banoub et al.

580.1945	579.1867	581.2023	C31 H32 O11	31	32	11	16.0	Trimer	TR20	Banoub et al.
582.2112	581.2034	583.2190	C31 H34 O11	31	34	11	15.0	Trimer	TR21	Morreel et al.
584.2258	583.2180	585.2336	C31 H36 O11	31	36	11	14.0	Trimer	TR22	Kiyota et al.
584.2258	583.2180	585.2336	C31 H36 O11	31	36	11	14.0	Trimer	TR23	Kiyota et al.
586.2426	585.2348	587.2504	C31 H38 O11	31	38	11	13.0	Trimer	TR24	Morreel et al.
602.2363	601.2285	603.2441	C31 H38 O12	31	38	12	13.0	Trimer	TR25	Kiyota et al.
606.2254	605.2176	607.2332	C37 H34 O8	37	34	8	21.0	Tetramer	TE1	Banoub et al.
614.2372	613.2294	615.2450	C32 H38 O12	32	38	12	14.0	Trimer	TR26	Morreel et al.
614.2376	613.2298	615.2454	C32 H38 O12	32	38	12	14.0	Trimer	TR27	Morreel et al.
614.2377	613.2299	615.2455	C32 H38 O12	32	38	12	14.0	Trimer	TR28	Morreel et al.
628.2308	627.2230	629.2386	C36 H36 O10	36	36	10	19.0	Tetramer	T13	Found in KL*
628.2531	627.2453	629.2609	C33 H40 O12	33	40	12	14.0	Trimer	TR29	Morreel et al.
630.2325	629.2247	631.2403	C32 H38 O13	32	38	13	14.0	Trimer	TR30	Morreel et al.
632.2469	631.2391	633.2547	C32 H40 O13	32	40	13	13.0	Trimer	TR31	Kiyota et al.
632.2617	631.2539	633.2695	C36 H40 O10	36	40	10	17.0	Tetramer	T14	Found in KL*
636.1996	635.1918	637.2074	C37 H32 O10	37	32	10	22.0	Tetramer	TE2	Banoub et al.
644.2478	643.2400	645.2556	C33 H40 O13	33	40	13	14.0	Trimer	TR32	Morreel et al.
654.2465	653.2387	655.2543	C38 H38 O10	38	38	10	20.0	Tetramer	TE3	Banoub et al.
660.2423	659.2345	661.2501	C33 H40 O14	33	40	14	14.0	Trimer	TR33	Morreel et al.
662.2575	661.2497	663.2653	C33 H42 O14	33	42	14	13.0	Trimer	TR34	Kiyota et al.
684.2207	683.2129	685.2285	C38 H36 O12	38	36	12	21.0	Tetramer	TE4	Banoub et al.
748.2750	747.2672	749.2828	C40 H44 O14	40	44	14	19.0	Tetramer	TE5	Huis et al.
756.2418	755.2340	757.2496	C41 H40 O14	41	40	14	22.0	Tetramer	TE6	Banoub et al.
778.2846	777.2768	779.2924	C41 H46 O15	41	46	15	19.0	Tetramer	TE7	Morreel et al.
780.3004	779.2926	781.3082	C41 H48 O15	41	48	15	18.0	Tetramer	TE8	Morreel et al.
788.2833	787.2755	789.2911	C46 H44 O12	46	44	12	25.0	Pentamer	P1	Banoub et al.
810.3106	809.3028	811.3184	C42 H50 O16	42	50	16	18.0	Tetramer	TE9	Morreel et al.
810.3107	809.3029	811.3185	C42 H50 O16	42	50	16	18.0	Tetramer	TE10	Morreel et al.
826.3065	825.2987	827.3143	C42 H50 O17	42	50	17	18.0	Tetramer	TE11	Morreel et al.

830.2575	829.2497	831.2653	C47 H42 O14	47	42	14	27.0	Pentamer	P2	Banoub et al.
840.3224	839.3146	841.3302	C43 H52 O17	43	52	17	18.0	Tetramer	TE12	Morreel et al.
860.3044	859.2966	861.3122	C49 H48 O14	49	48	14	26.0	Pentamer	P3	Banoub et al.
876.2993	875.2915	877.3071	C49 H48 O15	49	48	15	26.0	Pentamer	P4	Banoub et al.
890.3150	889.3072	891.3228	C50 H50 O15	50	50	15	26.0	Pentamer	P5	Banoub et al.
906.2888	905.2810	907.2966	C53 H46 O14	53	46	14	31.0	Hexamer	HEX1	Banoub et al.
950.3150	949.3072	951.3228	C55 H50 O15	55	50	15	31.0	Hexamer	HEX2	Banoub et al.
1082.3725	1081.3647	1083.3803	C64 H58 O16	64	58	16	36.0	Heptamer	HEP1	Banoub et al.
1098.3674	1097.3596	1099.3752	C64 H58 O17	65	60	17	36.0	Heptamer	HEP2	Banoub et al.
1232.4662	1231.4584	1233.4740	C63 H76 O25	63	76	25	26.0	Hexamer	HEX3	Morreel et al.

Table S2 Experiments performed in the first DOE

Exp No	Capillary Voltage (kV)	Sheath gas flow rate (AU)	Auxiliary gas flow rate (AU)
1	2.0	50	20
2	3.0	50	20
3	2.0	70	20
4	3.0	70	20
5	2.0	50	40
6	3.0	50	40
7	2.0	70	40
8	3.0	70	40
9	2.5	60	30
10	2.5	60	30
11	2.5	60	30

Table S3 Experiments performed in the second DOE

Exp No	Sheath gas flow rate (AU)	Auxiliary gas flow rate (AU)
1	70	10
2	80	10
3	70	20
4	80	20
5	75	15
6	75	15
7	75	15

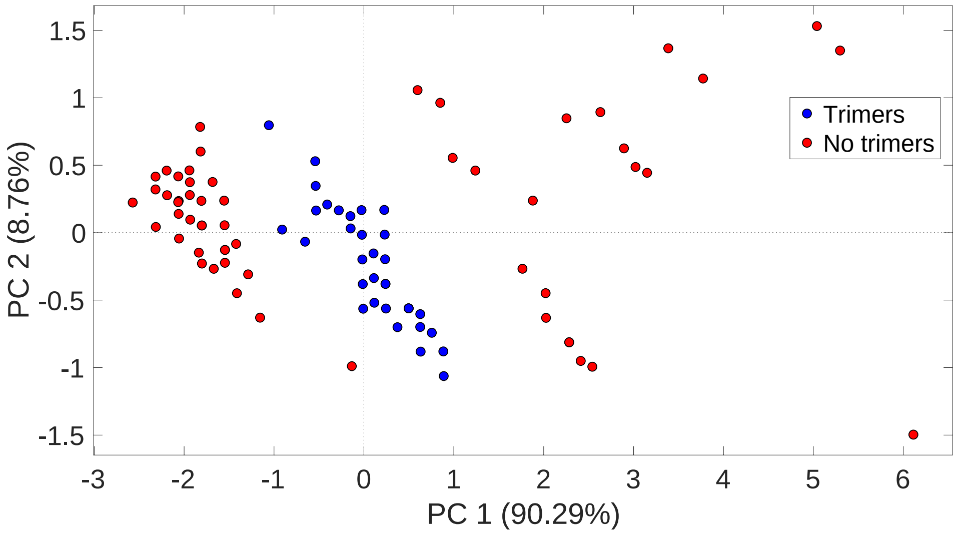

Fig. S1 Scores plot from the trimer classification model

Table S4 MZmine workflow and settings for peak identification in the Kraft lignin sample

1. Peak detection

1.1 Generation of mass list with Mass detection function (settings: polarity: negative; Mass detector: Exact mass; spectrum type: any)

1.2 FTMS shoulder peaks filter (settings: Mass resolution: 100000; peak model function: Gaussian)

1.3 Chromatogram builder (settings: Min time span (min): 0.05; Min height: 5.0E4; m/z tolerance: 0.01 m/z)

1.4 Chromatogram deconvolution (settings: algorithm: local minimum search; chromatographic threshold: 30%; search minimum in retention time range (min): 0.05; Minimum relative height: 5%; Minimum absolute height: 5.0E4; Min ratio of peak top/edge: 2; peak duration range (time) 0.00 – 10.00)

2. Deisotoping using Isotopic peak grouper (settings: m/z tolerance: 0.005 m/z; Maximum charge: 5; representative isotope: most intense)

3. Identification of adducts (settings: retention time tolerance: 0.05 min; m/z tolerance: 0.005 m/z; Max relative adduct peak height: 50%; included adducts: M+NH3; M+H2SO4; M+H2CO3; M+H3PO4; M+HCOO)

4. Alignment using Join aligner (settings: m/z tolerance: 0.005 m/z; weight for m/z: 2; retention time tolerance: 0.05 min; weight for retention time: 2)

Table S5 Detected MS^n fragments for LO 1

MS level (fragment)	Detected m/z	Lost fragment	Mass difference to $[M-H]^-$	Mass difference (mDa) of theoretical fragment mass - observed fragment mass	Determined elemental composition of detected mass	RDB	Mass difference (mDa) of theoretical mass - observed mass
MS1	243.0664	-	-	-	C14 H11 O4	9.5	1.20
MS2 (243.0664)	228.0426	CH3	15.0237	0.27	C13 H8 O4	10.0	0.92
MS3 (228.0426)	211.0400	CH3 + OH	32.0264	0.16	C13 H7 O3	10.5	1.04
	210.0322	CH3 + H2O	33.0341	0.11	C13 H6 O3	11.0	1.08
	200.0479	CH3 + CO	43.0184	0.04	C12 H8 O3	9.0	1.14
	182.0374	CH3 + CH2O2	61.0289	-0.02	C12 H6 O2	10.0	1.22
MS4 (210.0320)	182.0372	CH3 + H2O + CO	61.0286	-0.36	C12 H6 O2	10.0	0.38

Table S6 Detected MS^n fragments for LO 2

MS level (fragment)	Detected m/z	Lost fragment	Mass difference to $[M-H]^-$	Mass difference (mDa) of theoretical fragment mass - observed fragment mass	Determined elemental composition of detected mass	RDB	Mass difference (mDa) of theoretical mass - observed mass
MS1	245.0819	-	-	-	C14 H13 O4	8.5	1.11
MS2 (245.0819)	230.0582	CH3	15.0237	0.24	C13 H10 O4	9.0	0.87
	123.0454	C7H6O2	122.0365	-0.27	C7 H7 O2	4.5	1.37
	121.0298	C7H8O2	124.0521	-0.30	C7 H5 O2	5.5	1.40
	108.0220	C8H9O2	137.0599	-0.35	C6 H4 O2	5.0	1.46
MS3 (230.0582)	215.0348	CH3 + CH3	30.0472	0.21	C12 H7 O4	9.5	0.89
	121.0297	CH3 + C6H5O2	124.0522	-0.19	C7 H5 O2	5.5	1.29
	109.0298	CH3 + C7H5O2	136.0521	-0.28	C6 H5 O2	4.5	1.38
	108.0220	CH3 + C7H6O2	137.0600	-0.29	C6 H4 O2	5.0	1.40

Table S7 Detected MS^n fragments for LO 3

MS level (fragment)	Detected m/z	Lost fragment	Mass difference to $[M-H]^-$	Mass difference (mDa) of theoretical fragment mass - observed fragment mass	Determined elemental composition of detected mass	RDB	Mass difference (mDa) of theoretical mass - observed mass
MS1	257.0819	-	-	-	C15 H13 O4	9.5	0.53
MS2 (257.0819)	242.0581	CH3	15.0238	0.38	C14 H10 O4	10.0	0.70
MS3 (242.0581)	227.0347	CH3 + CH3	30.0472	0.35	C13 H7 O4	10.5	0.28
	225.0554	CH3 + OH	32.0265	0.28	C14 H9 O3	10.5	0.25
	224.0476	CH3 + H2O	33.0343	0.25	C14 H8 O3	11.0	0.28
	214.0634	CH3 + CO	43.0186	0.16	C13 H10 O3	9.0	0.36

Table S8 Detected MS^n fragments for LO 4

MS level (fragment)	Detected m/z	Lost fragment	Mass difference to $[M-H]^-$	Mass difference (mDa) of theoretical fragment mass - observed fragment mass	Determined elemental composition of detected mass	RDB	Mass difference (mDa) of theoretical mass - observed mass
MS1	259.0971	-	-	-	C15 H15 O4	8.5	0.84
MS2 (259.0971)	244.0737	CH3	15.0236	0.17	C14 H12 O4	9.0	0.67
	229.0504	CH3 + CH3	30.0469	-0.04	C13 H9 O4	9.5	0.89
MS3 (244.0971)	229.0503	CH3 + CH3	30.0470	0.03	C13 H9 O4	9.5	0.81
MS4 (229.0503)	212.0476	CH3 + CH3 + OH	47.0571	-0.33	C13 H8 O3	10.0	0.28
	211.0399	CH3 + CH3 + H2O	48.0571	-0.42	C13 H7 O3	10.5	0.37
	201.0556	CH3 + CH3 + CO	58.0414	-0.46	C12 H9 O3	8.5	0.40
	199.0401	CH3 + CH3 + CH2O	60.0569	-0.60	C12 H7 O3	9.5	0.55
	187.0400	CH3 + CH3 + C2H2O	72.0569	-0.57	C11 H7 O3	8.5	0.52
	185.0608	CH3 + CH3 + CO2	74.0362	-0.57	C12 H9 O2	8.5	0.53
	183.0449	CH3 + CH3 + CH2O2	76.0521	-0.37	C12 H7 O2	9.5	0.32
	173.0608	CH3 + CH3 + C2O2	86.0362	-0.60	C11 H9 O2	7.5	0.56
	159.0451	CH3 + CH3 + C3H2O2	100.0519	-0.53	C10 H7 O2	7.5	0.49
MS5 (211.0398)	183.0447	CH3 + CH3 + H2O + CO	76.0521	-0.34	C12 H7 O2	9.5	0.14

Table S9 Detected MS^n fragments for LO 5

MS level (fragment)	Detected m/z	Lost fragment	Mass difference to $[M-H]^-$	Mass difference (mDa) of theoretical fragment mass - observed fragment mass	Determined elemental composition of detected mass	RDB	Mass difference (mDa) of theoretical mass - observed mass
MS1	269.0816	-	-	-	C16 H13 O4	10.5	0.25
MS2 (269.0816)	254.0580	CH3	15.0237	0.18	C15 H10 O4	11.0	0.07
MS3 (254.0580)	237.0554	CH3 + OH	32.0262	-0.01	C15 H9 O3	11.5	0.25
	236.0476	CH3 + H2O	33.0340	0.00	C15 H8 O3	12.0	0.25
	226.0633	CH3 + CO	43.0183	-0.05	C14 H10 O3	10.0	0.30
	225.0555	CH3 + CHO	44.0261	-0.08	C14 H9 O3	10.5	0.33
	209.0607	CH3 + CHO2	60.0209	-0.23	C14 H9 O2	10.5	0.48
	161.0245	CH3 + C6H5O	108.0571	-0.38	C9 H5 O3	7.5	0.62
MS4 (236.0472)	208.0522	CH3 + H2O + CO	61.0291	0.09	C14 H8 O2	11.0	-0.21
MS4 (226.0631)	198.0684	CH3 + CO + CO	71.0127	-0.57	C13 H10 O2	9.0	0.31
	197.0606	CH3 + CO + CHO	72.0205	-0.59	C13 H9 O2	9.5	0.33

Table S10 Detected MS^n fragments for LO 6

MS level (fragment)	Detected m/z	Lost fragment	Mass difference to $[M-H]^-$	Mass difference (mDa) of theoretical fragment mass - observed fragment mass	Determined elemental composition of detected mass	RDB	Mass difference (mDa) of theoretical mass - observed mass
MS1	271.0975	-	-	-	C16 H15 O4	9.5	0.50
MS2 (271.0975)	256.0737	CH3	15.0238	0.36	C15 H12 O4	10.0	0.14
MS3 (256.0737)	255.0660	CH3 + H	16.0315	0.21	C15 H11 O4	10.5	0.29
	241.0503	CH3 + CH3	30.0472	0.26	C14 H9 O4	10.5	0.24
	240.0425	CH3 + CH4	31.0550	0.27	C14 H8 O4	11.0	0.24
	239.0711	CH3 + OH	32.0264	0.20	C15 H11 O3	10.5	0.30
	238.0633	CH3 + H20	33.0342	0.17	C15 H10 O3	11.0	0.33
	299.0504	CH3 + C2H3	42.0471	0.14	C13 H9 O4	9.5	0.37
	147.0453	CH3 + C6H5O2	124.0522	-0.18	C9 H7 O2	6.5	0.69
	146.0375	CH3 + C6H6O2	125.0600	-0.21	C9 H6 O2	7.0	0.71
	134.0375	CH3 + C7H6O2	137.0600	-0.25	C8 H6 O2	6.0	0.76
	109.0298	CH3 + C9H7O2	162.0677	-0.37	C6 H5 O2	4.5	0.87
	108.0220	CH3 + C9H8O2	163.0677	-0.38	C6 H4 O2	5.0	0.87
MS4 (241.0503)	224.0478	CH3 + CH3 + OH	47.0489	-0.79	C14 H8 O3	11.0	0.44
	223.0394	CH3 + CH3 + H2O	48.0573	-0.19	C14 H7 O3	11.5	-0.17
	213.0550	CH3 + CH3 + CO	58.0417	-0.17	C13 H9 O3	9.5	-0.19
	212.0473	CH3 + CH3 + CHO	59.0439	-0.36	C13 H8 O3	10.0	0.01
	195.0458	CH3 + CH3 + CH2O2	76.0508	-1.59	C13 H7 O2	10.5	1.24
MS4 (239.0712)	211.0606	CH3 + OH + H2O	50.0365	-0.25	C15 H9 O2	11.5	0.33
	145.0292	CH3 + OH + C6H6O	126.0677	-0.33	C9 H5 O2	7.5	0.41
	93.0346	CH3 + OH + C9H6O2	178.0625	-0.44	C6 H5 O	4.5	0.52

Table S11 Detected MS^n fragments for LO 7

MS level (fragment)	Detected m/z	Lost fragment	Mass difference to $[M-H]^-$	Mass difference (mDa) of theoretical fragment mass - observed fragment mass	Determined elemental composition of detected mass	RDB	Mass difference (mDa) of theoretical mass - observed mass
MS1	273.0768	-	-	-	C15 H13 O5	9.5	1.03
MS2 (273.0768)	258.0529	CH3	15.0239	0.44	C14 H10 O5	10.0	0.62
MS3 (258.0529)	257.0453	CH3 + H	16.0315	0.16	C14 H9 O5	10.5	0.32
	243.0296	CH3 + CH3	30.0471	0.20	C13 H7 O5	10.5	0.28
	241.0504	CH3 + OH	32.0263	0.13	C14 H9 O4	10.5	0.37
	240.0426	CH3 + H2O	33.0342	0.12	C14 H8 O4	11.0	0.36
	239.0349	CH3 + H2O + H	34.0419	0.04	C14 H7 O4	11.5	0.45
	229.0504	CH3 + CHO	44.0264	0.14	C13 H9 O4	9.5	0.35
	212.0478	CH3 + CH2O2	61.0290	0.01	C13 H8 O3	10.0	0.48
	211.0400	CH3 + CH3O2	62.0368	-0.01	C13 H7 O3	10.5	0.49
MS4 (243.0344)	215.0344	CH3 + CH3 + CO	58.0415	-0.34	C12 H7 O4	9.5	-0.04
	199.0396	CH3 + CH3 + CO2	74.0363	-0.43	C12 H7 O3	9.5	0.06

Table S12 Detected MS^n fragments for LO 8

MS level (fragment)	Detected m/z	Lost fragment	Mass difference to $[M-H]^-$	Mass difference (mDa) of theoretical fragment mass - observed fragment mass	Determined elemental composition of detected mass	RDB	Mass difference (mDa) of theoretical mass - observed mass
MS1	273.1128	-	-	-	C16 H17 O4	8.5	0.99
MS2 (273.1128)	258.0894	CH3	15.0237	0.24	C15 H14 O4	9.0	0.76
	243.0661	CH3 + CH3	30.0471	0.12	C14 H11 O4	9.5	0.88
MS3 (243.0661)	228.0426	CH3 + CH3 + CH3	45.0705	0.11	C13 H8 O4	10.0	0.89
	226.0634	CH3 + CH3 + OH	47.0497	0.04	C14 H10 O3	10.0	0.95
	225.0555	CH3 + CH3 + H2O	48.0576	0.12	C14 H9 O3	10.5	0.88
	215.0713	CH3 + CH3 + CO	58.0419	0.01	C13 H11 O3	8.5	0.99
MS3 (258.0894)	243.0659	CH3 + CH3	30.0462	-0.70	C14 H11 O4	9.5	0.13
MS4 (243.0657)	228.0426	CH3 + CH3 + CH3	45.0702	0.25	C13 H8 O4	10.0	0.36
	225.0554	CH3 + CH3 + CH3 + H2O	48.0574	-0.16	C14 H9 O3	10.5	0.27
MS5 (228.0428)	211.0401	CH3 + CH3 + CH3 + OH	62.0728	0.16	C13 H7 O3	10.5	0.56
	210.0318	CH3 + CH3 + CH3 + H2O	63.0810	0.16	C13 H6 O3	11.0	0.16

Table S13 Detected MS^n fragments for LO 9

MS level (fragment)	Detected m/z	Lost fragment	Mass difference to $[M-H]^-$	Mass difference (mDa) of theoretical fragment mass - observed fragment mass	Determined elemental composition of detected mass	RDB	Mass difference (mDa) of theoretical mass - observed mass
MS1	287.0926	-	-	-	C16 H15 O5	9.5	0.61
MS2 (287.0926)	272.0685	CH3	15.0240	0.55	C15 H12 O5	10.0	0.07
	136.0168	C9H11O2	151.0758	-0.13	C7 H4 O3	6.0	0.74
	108.0220	C10H11O3	179.0706	-0.26	C6 H4 O2	5.0	0.87
MS3 (272.0685)	257.0454	CH3 + CH3	30.0472	0.23	C14 H9 O5	10.5	0.38
	150.0324	CH3 + C7H6O2	137.0602	-0.08	C8 H6 O3	6.0	0.70
	136.0168	CH3 + C8H8O2	151.0758	-0.12	C7 H4 O3	6.0	0.73
	108.0220	CH3 + C9H8O3	179.0706	-0.25	C6 H4 O2	5.0	0.86
MS4 (257.0454)	239.0340	CH3 + CH3 + H2O	48.0570	-0.48	C14 H7 O4	11.5	-0.40
	229.0496	CH3 + CH3 + CO	58.0415	-0.37	C13 H9 O4	9.5	-0.52
	215.0340	CH3 + CH3 + C2H2O	72.0571	-0.41	C12 H7 O4	9.5	-0.48
	213.0547	CH3 + CH3 + CO2	74.0364	-0.37	C13 H9 O3	9.5	-0.51
	201.0547	CH3 + CH3 + C2O2	86.0364	-0.29	C12 H9 O3	8.5	-0.50
MS5 (229.0506)	211.0401	CH3 + CH3 + CO + H2O	76.0520	-0.47	C13 H7 O3	10.5	0.63
	201.0557	CH3 + CH3 + CO + CO	86.0364	-0.34	C12 H9 O3	8.5	0.49
	185.0609	CH3 + CH3 + CO + CO2	102.0313	-0.44	C12 H9 O2	8.5	0.60
	157.0656	CH3 + CH3 + CO + C2O3	130.0265	-0.14	C11 H9 O1	7.5	0.29

Table S14 Detected MS^n fragments for LO 10

MS level (fragment)	Detected m/z	Lost fragment	Mass difference to $[M-H]^-$	Mass difference (mDa) of theoretical fragment mass - observed fragment mass	Determined elemental composition of detected mass	RDB	Mass difference (mDa) of theoretical mass - observed mass
MS1	299.0918	-	-	-	C17 H15 O5	10.5	-0.17
MS2 (299.0918)	284.0685	CH3	15.0236	0.15	C16 H12 O5	11.0	-0.31
	269.0450	CH3 + CH3	30.0467	-0.26	C15 H9 O5	11.5	0.26
MS3 (284.0685)	269.0453	CH3 + CH3	30.0471	0.11	C15 H9 O5	11.5	-0.02
MS3 (269.0450)	251.0348	CH3 + CH3 + H2O	48.0573	-0.15	C15 H7 O4	12.5	0.33
	241.0504	CH3 + CH3 + CO	58.0417	-0.21	C14 H9 O4	10.5	0.34
	225.0555	CH3 + CH3 + CO2	74.0366	-0.190	C14 H9 O3	10.5	0.31
	197.0607	CH3 + CH3 + C2O3	102.0314	-0.29	C13 H9 O2	9.5	0.42
MS4 (269.0453)	251.0338	CH3 + CH3 + H2O	48.0569	-0.67	C15 H7 O4	12.5	-0.62
	241.0495	CH3 + CH3 + CO	58.0412	-0.72	C14 H9 O4	10.5	-0.57
	225.0547	CH3 + CH3 +CO2	74.0360	-0.82	C14 H9 O3	10.5	-0.47
	197.0599	CH3 + CH3 + C2O3	102.0307	-0.94	C13 H9 O2	9.5	-0.33
MS5 (241.0495)	224.0467	CH3 + CH3 + CO + OH	75.0439	-0.67	C14 H8 O3	11.0	-0.60
	223.0390	CH3 + CH3 + CO + H2O	76.0517	-0.73	C14 H7 O3	11.5	-0.55
	213.0547	CH3 + CH3 + CO + CO	86.0360	-0.78	C13 H9 O3	9.5	-0.50
	197.0600	CH3 + CH3 + CO + CO2	102.0365	-1.00	C13 H9 O2	9.5	-0.27
	169.0651	CH3 + CH3 + CO + C2O3	130.0256	-1.02	C12 H9 O	8.5	-0.26
MS6 (213.0547)	195.0442	CH3 + CH3 + CO + CO + H2O	104.0465	-0.88	C13 H7 O2	10.5	-0.40
	185.0599	CH3 + CH3 + CO + CO + CO	114.0308	-0.88	C12 H9 O2	8.5	-0.39

Table S15 Detected MS^n fragments for LO 11

MS level (fragment)	Detected m/z	Lost fragment	Mass difference to $[M-H]^-$	Mass difference (mDa) of theoretical fragment mass - observed fragment mass	Determined elemental composition of detected mass	RDB	Mass difference (mDa) of theoretical mass - observed mass
MS1	301.0717	-	-	-	C16 H13 O6	10.5	0.48
MS2 (301.0717)	286.0477	CH3	15.0240	0.49	C15 H10 O6	11.0	-0.01
MS3 (286.0477)	285.0400	CH3 + H	16.0317	0.41	C15 H9 O6	11.5	0.07
	271.0243	CH3 + CH3	30.0474	0.41	C14 H7 O6	11.5	0.06
	269.0451	CH3 + OH	32.0266	0.37	C15 H9 O5	11.5	0.10
	268.0373	CH3 + H2O	33.0344	0.35	C15 H8 O5	12.0	0.12
	267.0295	CH3 + H2O + H	34.0422	0.32	C15 H7 O5	12.5	0.16
	258.0530	CH3 + CO	43.0187	0.26	C14 H10 O5	10.0	0.22
	257.0452	CH3 + CHO	44.0265	0.28	C14 H9 O5	10.5	0.20
	256.0375	CH3 + CH2O	45.0342	0.14	C14 H8 O5	11.0	0.34
	240.0425	CH3 + CH2O2	61.0292	0.27	C14 H8 O4	11.0	0.21
	239.0504	CH3 + CH3O2	62.0369	0.16	C14 H7 O4	11.5	0.31
	229.0504	CH3 + C2HO2	72.0212	0.11	C13 H9 O4	9.5	0.37
MS4 (271.0241)	243.0290	CH3 + CH3 + CO	58.0419	0.07	C13 H7 O5	10.5	-0.39
	227.0341	CH3 + CH3 + CO2	74.0368	-0.19	C13 H7 O4	10.5	-0.31
MS4 (268.0375)	240.0426	CH3 + H2O + CO	61.0284	-0.55	C14 H8 O4	11.0	0.36
	239.0348	CH3 + H2O + CHO	62.0362	-0.57	C14 H7 O4	11.5	0.38
	212.0476	CH3 + H2O + C2O2	89.0234	-0.43	C13 H8 O3	10.0	0.24
MS5 (243.0296)	215.0349	CH3 + CH3 + CO + CO	86.0361	-0.65	C12 H7 O4	9.5	1.00
MS5 (240.0427)	212.0478	CH3 + H2O + CO + CO	89.0236	-0.31	C13 H8 O3	10.0	0.42

Table S16 Detected MS^n fragments for LO 12

MS level (fragment)	Detected m/z	Lost fragment	Mass difference to $[M-H]^-$	Mass difference (mDa) of theoretical fragment mass - observed fragment mass	Determined elemental composition of detected mass	RDB	Mass difference (mDa) of theoretical mass - observed mass
MS1	313.1079	-	-	-	C18 H17 O5	10.5	0.34
MS2 (313.1079)	298.0840	CH3	15.0240	0.49	C17 H14 O5	11.0	-0.14
	283.0608	CH3 + CH3	30.0472	0.23	C16 H11 O5	11.5	0.11
MS3 (298.0840)	283.0608	CH3 + CH3	30.0474	0.23	C16 H11 O5	11.5	0.11
MS3 (283.0608)	265.0504	CH3 + CH3 + H2O	48.0575	-0.02	C16 H9 O4	12.5	0.34
	255.0660	CH3 + CH3 + CO	58.0419	0.01	C15 H11 O4	10.5	0.31
	241.0504	CH3 + CH3 + C2H2O	72.0575	-0.01	C14 H9 O4	10.5	0.32
	240.0427	CH3 + CH3 + C2H3O	73.0652	-0.11	C14 H8 O4	11.0	0.42
	239.0714	CH3 + CH3 + CO2	74.0365	-0.24	C15 H11 O3	10.5	0.55
	224.0479	CH3 + CH3 + C2H3O2	89.0601	-0.19	C14 H8 O3	11.0	0.51
MS4 (283.0608)	265.0497	CH3 + CH3 + H2O	48.0574	-0.12	C16 H9 O4	12.5	-0.34
	255.0655	CH3 + CH3 + CO	58.0147	-0.20	C15 H11 O4	10.5	-0.25
	241.0498	CH3 + CH3 + C2H2O	72.0573	-0.28	C14 H9 O4	10.5	-0.28
	240.0420	CH3 + CH3 + C2H3O	73.0652	-0.17	C14 H8 O4	11.0	-0.28
	239.0706	CH3 + CH3 + CO2	74.0365	-0.24	C15 H11 O3	10.5	-0.22
	237.0549	CH3 + CH3 + CH2O2	76.0522	-0.23	C15 H9 O3	11.5	-0.22
	224.0472	CH3 + CH3 + C2H3O2	89.0599	-0.33	C14 H8 O3	11.0	-0.11
MS5 (241.0502)	224.0475	CH3 + CH3 + C2H2O + OH	89.0595	-0.77	C14 H8 O3	11.0	0.21
	223.0397	CH3 + CH3 + C2H2O + H2O	90.0673	-0.79	C14 H7 O3	11.5	0.21
	213.0554	CH3 + CH3 + C2H2O + CO	100.0516	-0.81	C13 H9 O3	9.5	0.23
	197.0605	CH3 + CH3 + C2H2O + CO2	116.0516	-0.79	C13 H9 O2	9.5	0.22

Table S17 Detected MS^n fragments for LO 13

MS level (fragment)	Detected m/z	Lost fragment	Mass difference to $[M-H]^-$	Mass difference (mDa) of theoretical fragment mass - observed fragment mass	Determined elemental composition of detected mass	RDB	Mass difference (mDa) of theoretical mass - observed mass
MS1	315.1236	-	-	-	C18 H19 O5	9.5	0.32
MS2 (315.1236)	300.0997	CH3	15.0238	0.36	C17 H16 O5	10.0	-0.04
MS3 (300.0997)	299.0922	CH3 + H	16.0313	0.04	C17 H15 O5	10.5	0.27
	283.0972	CH3 + OH	32.0264	0.19	C17 H15 O4	11.5	0.11
	282.0894	CH3 + H2O	33.0341	0.11	C17 H14 O4	11.0	0.21
	270.0894	CH3 + CH2O	45.0342	0.17	C16 H14 O4	10.0	0.15
	269.0816	CH3 + CH3O	46.0420	0.10	C16 H13 O4	10.5	0.22
	257.0817	CH3 + C2H3O	58.0419	0.04	C15 H13 O4	9.5	0.29
	256.0739	CH3 + C2H4O	59.0497	-0.01	C15 H12 O4	10.0	0.32
	255.0660	CH3 + C2H5O	60.0576	0.06	C15 H11 O4	10.5	0.26
	251.0712	CH3 + CH5O2	64.0524	-0.06	C16 H11 O3	11.5	0.38
	243.0661	CH3 + C3H5O	72.0575	-0.02	C14 H11 O4	9.5	0.34
	241.0504	CH3 + C3H7O	74.0732	0.04	C14 H9 O4	10.5	0.29
	240.0425	CH3 + C3H8O	75.0810	0.05	C14 H8 O4	11.0	0.27
	191.0714	CH3 + C6H5O2	124.0522	-0.26	C11 H11 O3	6.5	0.58
	178.0636	CH3 + C7H6O2	137.0600	-0.30	C10 H10 O3	6.0	0.62
	148.0532	CH3 + C8H8O3	167.0704	-0.42	C9 H8 O2	6.0	0.73
MS4 (282.0894)	254.0954	CH3 + H2O + CO	61.0271	-1.87	C16 H14 O3	10.0	1.15
	252.0780	CH3 + H2O + CH2O	63.0445	-0.06	C16 H12 O3	11.0	-0.65
	251.0702	CH3 + H2O + CH3O	64.0524	-0.08	C16 H11 O3	11.5	-0.64
	238.0626	CH3 + H2O +	77.0599	-0.33	C15 H10 O3	11.0	-0.38

		C2H4O					
MS4 (269.0816)	254.0583	CH3 + CH3O + CH3	61.0517	-0.78	C15 H10 O4	11.0	0.36
	251.0712	CH3 + CH3O + H2O	64.0517	-0.75	C16 H11 O3	11.5	0.33
	241.0869	CH3 + CH3O + CO	74.0359	-0.86	C15 H13 O3	9.5	0.44
	240.0428	CH3 + CH3O + C2H5	75.0801	-0.91	C14 H8 O4	11.0	0.50
	159.0449	CH3 + CH3O + C6H6O2	156.0780	-0.67	C10 H7 O2	7.5	0.26
	147.0449	CH3 + CH3O + C7H6O2	168.0780	-0.68	C9 H7 O2	6.5	0.28
	135.0449	CH3 + CH3O + C8H6O2	180.0779	-0.73	C8 H7 O2	5.5	0.32
	121.0293	CH3 + CH3O + C9H8O2	194.0936	-0.74	C7 H5 O2	5.5	0.33
MS5 (251.0731)	223.0764	CH3 + CH3O + H2O + CO	92.0468	-0.56	C15 H11 O2	10.5	0.45

Table S18 Detected MS^n fragments for LO 14

MS level (fragment)	Detected m/z	Lost fragment	Mass difference to $[M-H]^-$	Mass difference (mDa) of theoretical fragment mass - observed fragment mass	Determined elemental composition of detected mass	RDB	Mass difference (mDa) of theoretical mass - observed mass
MS1	321.1131	-	-	-	C20 H17 O4	12.5	0.39
MS2 (321.1131)	306.0891	CH3	15.0240	0.52	C19 H14 O4	13.0	-0.13
MS3 (306.0891)	305.0816	CH3 + H	16.0315	0.16	C19 H13 O4	13.5	0.22
	291.0660	CH3 + CH3	30.0470	0.08	C18 H11 O4	13.5	0.31
	278.0947	CH3 + CO	43.0184	0.01	C18 H14 O3	12.0	0.38
	277.0870	CH3 + CHO	44.0260	-0.95	C18 H13 O3	12.5	0.57
	262.0997	CH3 + CO2	59.0134	0.07	C18 H14 O2	12.0	0.32
MS4 (291.0656)	263.0705	CH3 + CH3 + CO	58.0416	-0.31	C17 H11 O3	12.5	-0.35

Table S19 Detected MS^n fragments for LO 15

MS level (fragment)	Detected m/z	Lost fragment	Mass difference to $[M-H]^-$	Mass difference (mDa) of theoretical fragment mass - observed fragment mass	Determined elemental composition of detected mass	RDB	Mass difference (mDa) of theoretical mass - observed mass
MS1	335.0922	-	-	-	C20 H15 O5	13.5	0.21
MS2 (335.0922)	320.0683	CH3	15.0239	0.39	C19 H12 O5	14.0	-0.17
MS3 (320.0683)	292.0740	CH3 + CO	43.0182	-0.23	C18 H12 O4	13.0	0.45
	291.0659	CH3 + CHO	44.0262	0.01	C18 H11 O4	13.5	0.22

Table S20 Detected MS^n fragments for LO 16

MS level (fragment)	Detected m/z	Lost fragment	Mass difference to $[M-H]^-$	Mass difference (mDa) of theoretical fragment mass - observed fragment mass	Determined elemental composition of detected mass	RDB	Mass difference (mDa) of theoretical mass - observed mass
MS1	349.1077	-	-	-	C21 H17 O5	13.5	0.10
MS2 (349.1077)	334.0838	CH3	15.0239	0.46	C20 H14 O5	14.0	-0.35
MS3 (334.0838)	319.0607	CH3 + CH3	30.0470	0.02	C19 H11 O5	14.5	0.08
	316.0735	CH3 + H2O	33.0342	0.17	C20 H12 O4	15.0	-0.07
	306.0894	CH3 + CO	43.0182	-0.14	C19 H14 O4	13.0	0.24
	305.0815	CH3 + CHO	44.0262	0.10	C19 H13 O4	13.5	0.13
MS4 (319.0608)	291.0660	CH3 + CH3 + CO	58.0415	-0.34	C18 H11 O4	13.5	0.25
MS5 (291.0659)	263.0711	CH3 + CH3 + CO + CO	86.0363	-0.53	C17 H11 O3	12.5	0.32

Table S21 Detected MS^n fragments for LO 17

MS level (fragment)	Detected m/z	Lost fragment	Mass difference to $[M-H]^-$	Mass difference (mDa) of theoretical fragment mass - observed fragment mass	Determined elemental composition of detected mass	RDB	Mass difference (mDa) of theoretical mass - observed mass
MS1	351.1235	-	-	-	C21 H19 O5	12.5	0.29
MS2 (351.1235)	336.0995	CH3	15.0240	0.58	C20 H16 O5	13.0	-0.28
MS3 (336.0995)	321.0759	CH3 + CH3	30.0476	0.69	C19 H13 O5	13.5	-0.32
	319.0970	CH3 + OH	32.0265	0.28	C20 H15 O4	13.5	0.02
	318.0894	CH3 + H2O	33.0342	0.14	C20 H14 O4	14.0	0.15
MS4 (321.0757)	306.0526	CH3 + CH3 + CH3	45.0703	-0.15	C18 H10 O5	14.0	-0.27
	304.0732	CH3 + CH3 + OH	47.0496	-0.07	C19 H12 O4	14.0	-0.35
	293.0809	CH3 + CH3 + CO	58.0419	0.07	C18 H13 O4	12.5	-0.48
	277.0862	CH3 + CH3 + CO2	74.0366	-0.16	C18 H13 O3	12.5	-0.26
MS5 (293.0813)	275.0869	CH3 + CH3 + CO + H2O	76.0512	-1.22	C18 H11 O3	13.5	0.44
	265.0869	CH3 + CH3 + CO + CO	86.0356	-1.17	C17 H13 O3	11.5	0.38
	249.0922	CH3 + CH3 + CO + CO2	102.0303	-1.39	C17 H13 O2	11.5	0.61
	237.0921	CH3 + CH3 + CO + CO + CO	114.0304	-1.31	C16 H13 O2	10.5	0.54
MS6 (265.0869)	237.0920	CH3 + CH3 + CO + CO + CO	114.0307	-0.96	C16 H13 O2	10.5	0.49

Table S22 Detected MS^n fragments for LO 18

MS level (fragment)	Detected m/z	Lost fragment	Mass difference to $[M-H]^-$	Mass difference (mDa) of theoretical fragment mass - observed fragment mass	Determined elemental composition of detected mass	RDB	Mass difference (mDa) of theoretical mass - observed mass
MS1	353.1391	-	-	-	C21 H21 O5	11.5	0.17
MS2 (353.1391)	338.1153	CH3	15.0238	0.33	C20 H18 O5	12.0	-0.16
	190.0633	C10H11O2	163.0757	-0.18	C11 H10 O3	7.0	0.35
MS3 (190.0633)	175.0395	C10H11O2 + CH3	178.0989	-0.46	C10 H7 O3	7.5	0.02

Table S23 Detected MS^n fragments for LO 19

MS level (fragment)	Detected m/z	Lost fragment	Mass difference to $[M-H]^-$	Mass difference (mDa) of theoretical fragment mass - observed fragment mass	Determined elemental composition of detected mass	RDB	Mass difference (mDa) of theoretical mass - observed mass
MS1	357.1339	-	-	-	C20 H21 O6	10.5	0.04
MS2 (357.1339)	342.1101	CH3	15.0237	0.24	C19 H18 O6	11.0	-0.20
	339.1232	H2O	18.0107	0.12	C20 H19 O5	11.5	-0.08
	313.1329	CO2	43.9899	0.10	C19 H21 O4	9.5	-0.06
	298.1205	C2H3O2	59.0133	0.01	C18 H18 O4	10.0	0.03
	295.1336	CH2O3	62.0002	-0.18	C19 H19 O3	10.5	0.22
	281.1179	C2H4O3	76.0159	-0.11	C18 H17 O3	10.5	0.15
	211.0817	C8H8O2	136.0521	-0.29	C12 H13 O4	6.5	0.33
	209.0818	C9H8O2	148.0521	-0.34	C11 H13 O4	5.5	0.38
	191.0713	C9H10O3	166.0626	-0.41	C11 H11 O3	6.5	0.45
	161.0608	C10H12O4	196.0730	-0.55	C10 H9 O2	6.5	0.59
	147.0452	C11H14O4	210.0886	-0.60	C9 H7 O2	6.5	0.64
	121.0297	C13H16O4	236.1041	-0.75	C7 H5 O2	5.5	0.79
MS3 (313.1329)	298.1205	CO2 + CH3	59.0140	-0.27	C18 H18 O4	10.0	0.03
	283.0971	CO2 + CH3 + CH3	74.0365	-0.31	C17 H15 O4	10.5	0.08
	161.0608	CO2 + C9H12O2	196.0728	-0.75	C10 H9 O2	6.5	0.51
MS4 (298.1210)	283.0973	CO2 + CH3 + CH3	74.0363	-0.46	C17 H15 O4	10.5	0.26
	161.0605	CO2 + CH3 + C8H9O2	196.0731	-0.42	C10 H9 O2	6.5	0.22
	147.0449	CO2 + CH3 + C9H11O2	210.0887	-0.54	C9 H7 O2	6.5	0.34

Table S24 Detected MS^n fragments for LO 20

MS level (fragment)	Detected m/z	Lost fragment	Mass difference to $[M-H]^-$	Mass difference (mDa) of theoretical fragment mass - observed fragment mass	Determined elemental composition of detected mass	RDB	Mass difference (mDa) of theoretical mass - observed mass
MS1	361.1652	-	-	-	C20 H25 O6	8.5	0.08
MS2 (361.1652)	346.1416	CH3	15.0236	0.12	C19 H22 O6	9.0	-0.04
	343.1547	H2O	18.0105	-0.75	C20 H23 O5	9.5	0.14
	331.1547	CH2O	30.0105	-0.04	C19 H23 O5	8.5	0.11
	313.1441	CH4O2	48.0211	-0.01	C19 H21 O4	9.5	0.09
	298.1207	C2H7O2	63.0445	-0.11	C18 H18 O4	10.0	0.19
	179.0713	C10H14O3	182.0939	-0.43	C10 H11 O3	5.5	0.51
	165.0557	C11H16O3	196.1095	-0.49	C9 H9 O3	5.5	0.57
	122.0376	C13H19O4	239.1276	-0.70	C7 H6 O2	5.0	0.78
MS3 (346.1416)	327.1234	CH3 + H3O	34.0417	-0.12	C19 H19 O5	10.5	0.19
	315.1234	CH3 + CH3O	46.018	-0.09	C18 H19 O5	9.5	0.16
	223.0973	CH3 + C7H7O2	138.0679	-0.21	C12 H15 O4	5.5	0.29
	179.0714	CH3 + C9H11O3	182.0938	-0.46	C10 H11 O3	5.5	0.55
	165.0558	CH3 + C10H13O3	196.1094	-0.50	C9 H9 O3	5.5	0.59
	147.0453	CH3 + C10H15O4	214.1199	-0.58	C9 H7 O2	6.5	0.66
	136.0531	CH3 + C11H14O4	225.1121	-0.62	C8 H8 O2	5.0	0.70
	122.0375	CH3 + C12H16O4	239.1277	-0.65	C7 H6 O2	5.0	0.73
	109.0297	CH3 + C13H17O4	252.1354	-0.72	C6 H5 O2	4.5	0.80
MS3 (313.1441)	298.1206	CH4O2 + CH3	63.0439	-0.69	C18 H18 O4	10.0	0.10
	175.0764	CH4O2 + C8H10O2	186.0881	-1.09	C11 H11 O2	6.5	0.50
	162.0686	CH4O2 + C9H11O2	199.0959	-1.14	C10 H10 O2	6.0	0.55
MS4 (223.0965)	208.0731	CH3 + C7H7O2 + CH3	153.0907	-0.83	C11 H12 O4	6.0	-0.44
MS4 (165.0556)	147.0451	CH3 + C10H13O3 + H2O	214.1197	-0.84	C9 H7 O2	6.5	0.46

Table S25 Detected MS^n fragments for LO 21

MS level (fragment)	Detected m/z	Lost fragment	Mass difference to $[M-H]^-$	Mass difference (mDa) of theoretical fragment mass - observed fragment mass	Determined elemental composition of detected mass	RDB	Mass difference (mDa) of theoretical mass - observed mass
MS1	373.1288	-	-	-	C20 H21 O7	10.5	0.08
MS2 (373.1288)	358.1052	CH3	15.0236	0.12	C19 H18 O7	11.0	-0.03
	355.1179	H2O	18.0109	0.33	C20 H19 O6	11.5	-0.24
	340.0946	CH5O	33.0342	0.17	C19 H16 O6	12.0	-0.09
	327.1231	CH2O2	46.0056	0.23	C19 H19 O5	10.5	-0.14
	311.1284	CH2O3	62.0004	0.01	C19 H19 O4	10.5	0.09
	249.0765	C7H8O2	124.0523	-0.09	C13 H13 O5	7.5	0.18
	235.0609	C8H10O2	138.0679	-0.15	C12 H11 O5	7.5	0.23
	223.0609	C9H10O2	150.0679	-0.15	C11 H11 O5	6.5	0.23
	221.0453	C9H12O2	152.0835	-0.23	C11 H9 O5	7.5	0.32
	176.0479	C10H13O4	197.0810	0.42	C10 H8 O3	7.0	0.51
	136.0531	C12H13O5	237.0757	-0.62	C8 H8 O2	5.0	0.70
MS3 (355.1179)	340.0944	H2O + CH3	33.0344	0.35	C19 H16 O6	12.0	-0.27
	337.1074	H2O + H2O	36.0214	0.29	C20 H17 O5	12.5	-0.21
	327.1233	H2O + CO	46.0056	0.07	C19 H19 O5	10.5	0.01
	311.1283	H2O + CO2	62.0005	0.10	C19 H19 O4	10.5	-0.01
	204.0426	H2O + C9H11O2	169.0862	-0.26	C11 H8 O4	8.0	0.34
MS3 (327.1231)	312.0995	CH2O2 + CH3	61.0287	-0.26	C18 H16 O5	11.0	-0.25
	147.0452	CH2O2 + C10H12O3	226.0831	-1.07	C9 H7 O2	6.5	0.55
MS4 (312.0995)	297.0629	CH2O2 + CH3 + CH3	76.0504	-1.98	C17 H13 O5	11.5	1.34
	147.0447	CH2O2 + CH3 + C9H9O3	226.0828	-1.31	C9 H7 O2	6.5	0.67
	122.0369	CH2O2 + CH3 + C11H10O3	251.0906	-1.33	C7 H6 O2	5.0	0.68

Table S26 Detected MS^n fragments for LO 22

MS level (fragment)	Detected m/z	Lost fragment	Mass difference to $[M-H]^-$	Mass difference (mDa) of theoretical fragment mass - observed fragment mass	Determined elemental composition of detected mass	RDB	Mass difference (mDa) of theoretical mass - observed mass
MS1	377.1387	-	-	-	C23 H21 O5	13.5	0.35
MS2 (377.1387)	362.1150	CH3	15.0237	0.24	C22 H18 O5	14.0	0.12
MS3 (362.1150)	347.0917	CH3 + CH3	30.0470	0.02	C21 H15 O5	14.5	0.34
MS4 (347.0917)	332.0672	CH3 + CH3 + CH3	45.0702	-0.24	C20 H12 O5	15.0	-1.83
	329.0801	CH3 + CH3 + H2O	48.0702	-0.21	C21 H13 O4	15.5	-1.30
	319.0957	CH3 + CH3 + CO	58.0517	-0.18	C20 H15 O4	13.5	1.31
	303.1010	CH3 + CH3 + CO2	74.0364	-0.37	C20 H15 O3	13.5	-1.14
	291.1011	CH3 + CH3 + CO + CO	86.0363	-0.53	C19 H15 O3	12.5	-0.98
	238.0622	CH3 + CH3 + C6H5O2	139.0752	-0.68	C15 H10 O3	11.0	-0.83
MS5 (319.0973)	304.0973	CH3 + CH3 + CO + CH3	73.0644	-0.95	C19 H12 O4	14.0	0.29
	291.1024	CH3 + CH3 + CO + CO	86.0359	-0.89	C19 H15 O3	12.5	0.24
	213.0555	CH3 + CH3 + CO + C7H6O	164.0828	-0.97	C13 H9 O3	9.5	0.31
	185.0606	CH3 + CH3 + CO + C8H6O2	192.0777	-0.96	C12 H9 O2	8.5	0.31
MS5 (291.1028)	185.0607	CH3 + CH3 + CO + CO + C7H6O	192.0778	-0.87	C12 H9 O2	8.5	0.43

Table S27 Detected MS^n fragments for LO 23

MS level (fragment)	Detected m/z	Lost fragment	Mass difference to $[M-H]^-$	Mass difference (mDa) of theoretical fragment mass - observed fragment mass	Determined elemental composition of detected mass	RDB	Mass difference (mDa) of theoretical mass - observed mass
MS1	393.1332	-	-	-	C23 H21 O6	13.5	0.38
MS2 (393.1332)	378.1096	CH3	15.0240	0.52	C22 H18 O6	14.0	-0.14
	363.0864	CH3 + CH3	30.0471	0.17	C21 H15 O6	14.5	0.22
MS3 (378.1096)	363.0864	CH3 + CH3	30.0472	0.12	C21 H15 O6	14.5	0.13
MS3 (363.0864)	348.0630	CH3 + CH3 + CH3	45.0706	0.23	C20 H12 O6	15.0	0.18
	345.0761	CH3 + CH3 + H2O	48.0576	0.04	C21 H13 O5	15.5	0.36
	335.0917	CH3 + CH3 + CO	58.0420	0.10	C20 H15 O5	13.5	0.31
	240.0424	CH3 + CH3 + C7H7O2	153.0913	-0.27	C14 H8 O4	11.0	0.66
MS4 (363.0864)	348.0623	CH3 + CH3 + CH3	45.0699	-0.54	C20 H12 O6	15.0	-1.10
	345.0753	CH3 + CH3 + H2O	48.0569	-0.64	C21 H13 O5	15.5	-1.01
	335.0911	CH3 + CH3 + CO	58.0410	-0.82	C20 H15 O5	13.5	-0.82
	240.0416	CH3 + CH3 + C7H7O2	153.0905	-1.03	C14 H8 O4	11.0	-0.63
MS5 (348.0633)	330.0534	CH3 + CH3 + CH3 + H2O	63.0798	-1.16	C20 H10 O5	16.0	0.56
	320.0690	CH3 + CH3 + CH3 + CO	73.0642	-1.16	C19 H12 O5	14.0	0.56
MS5 (330.0531)	302.0580	CH3 + CH3 + CH3 + H2O + CO	91.0753	-0.59	C19 H10 O4	15.0	0.10

Table S28 Detected MS^n fragments for LO 24

MS level (fragment)	Detected m/z	Lost fragment	Mass difference to $[M-H]^-$	Mass difference (mDa) of theoretical fragment mass - observed fragment mass	Determined elemental composition of detected mass	RDB	Mass difference (mDa) of theoretical mass - observed mass
MS1	395.1132	-	-	-	C22 H19 O7	13.5	0.11
MS2 (395.1132)	351.1227	CO2	43.9905	0.71	C21 H19 O5	12.5	-0.60
MS3 (351.1227)	336.0993	CO2 + CH3	59.0139	0.55	C20 H16 O5	13.0	-0.44
	335.0916	CO2 + CH3 + H	60.0216	0.48	C20 H15 O5	13.5	-0.37
	334.0842	CO2 + CH3 + 2 x H	61.0290	0.07	C20 H14 O5	14.0	0.05
	321.1127	CO2 + CH2O	74.0005	0.07	C20 H17 O4	12.5	0.05
MS4 (336.0993)	335.0909	CO2 + CH3 + H	60.0210	-0.69	C20 H15 O5	13.5	-1.04
	321.0752	CO2 + CH3 + CH3	74.0367	-0.13	C19 H13 O5	13.5	-1.04
	320.0672	CO2 + CH3 + CH3 + H	75.0447	0.11	C19 H12 O5	14.0	-1.30
MS5 (320.0672)	292.0742	CO2 + CH3 + CH3 + H + CO	103.0385	-0.98	C18 H12 O4	13.0	0.66

Table S29 Detected MS^n fragments for LO 25

MS level (fragment)	Detected m/z	Lost fragment	Mass difference to $[M-H]^-$	Mass difference (mDa) of theoretical fragment mass - observed fragment mass	Determined elemental composition of detected mass	RDB	Mass difference (mDa) of theoretical mass - observed mass
MS1	395.1494	-	-	-	C23 H23 O6	12.5	-0.02
MS2 (395.1494)	380.1255	CH3	15.0239	0.43	C22 H20 O6	13.0	-0.44
	271.0969	C7H8O2	124.0525	0.09	C16 H15 O4	9.5	-0.11
MS3 (380.1255)	365.1020	CH3 + CH3	30.0475	0.48	C21 H17 O6	13.5	-0.52
MS3 (271.0976)	256.0739	C7H8O2 + CH3	139.0750	-0.93	C15 H12 O4	10.0	0.38
	239.0712	C7H8O2 + CH4O	156.0778	-0.88	C15 H11 O3	10.5	0.35
MS4 (256.0739)	241.0503	C7H8O2 + CH3 + CH3	154.0991	-0.30	C14 H9 O4	10.5	0.23
	239.0712	C7H8O2 + CH3 + OH	156.0782	-0.41	C15 H11 O3	10.5	0.33
	238.0633	C7H8O2 + CH3 + H2O	157.0861	-0.41	C15 H10 O3	11.0	0.33
	227.0711	C7H8O2 + CH3 + CHO	168.0783	-0.32	C14 H11 O3	9.5	0.24

Table S30 Detected MS^n fragments for LO 26

MS level (fragment)	Detected m/z	Lost fragment	Mass difference to $[M-H]^-$	Mass difference (mDa) of theoretical fragment mass - observed fragment mass	Determined elemental composition of detected mass	RDB	Mass difference (mDa) of theoretical mass - observed mass
MS1	419.1495	-	-	-	C25 H23 O6	14.5	0.63
MS2 (419.1495)	404.1253	CH3	15.0242	0.73	C24 H20 O6	15.0	-0.10
	389.1027	CH3 + CH3	30.0468	-0.11	C23 H17 O6	15.5	0.73
MS3 (404.1253)	389.1018	CH3 + CH3	30.0478	0.81	C23 H17 O6	15.5	-0.19
MS3 (389.1027)	374.0785	CH3 + CH3 + CH3	45.0703	-0.95	C22 H14 O6	16.0	0.00
	371.0916	CH3 + CH3 + H2O	48.0579	-0.30	C23 H15 O5	16.5	0.21
	361.0916	CH3 + CH3 + CO	58.0415	-0.37	C22 H17 O5	14.5	0.28
	240.0424	CH3 + CH3 + C9H9O2	179.1065	-0.73	C14 H8 O4	11.0	0.65
MS4 (389.1021)	374.0790	CH3 + CH3 + CH3	45.0692	-1.25	C22 H14 O6	16.0	-0.06
MS5 (374.0790)	373.0699	CH3 + CH3 + CH3 + H	46.0777	-0.58	C22 H13 O6	16.5	-1.51
	357.0762	CH3 + CH3 + CH3 + OH	62.0719	-1.24	C22 H13 O5	16.5	-0.07
	356.0685	CH3 + CH3 + CH3 + H2O	63.0796	-1.37	C22 H12 O5	17.0	0.07
	345.0764	CH3 + CH3 + CH3 + CHO	74.0717	-1.42	C21 H13 O5	15.5	0.12
	252.0425	CH3 + CH3 + CH3 + C7H6O2	167.1057	-1.53	C15 H8 O4	12.0	0.22
MS6 (357.0761)	339.0659	CH3 + CH3 + CH3 + OH + H2O	80.0826	-1.15	C22 H11 O4	17.5	0.16
	329.0816	CH3 + CH3 + CH3 + OH + CO	90.0668	-1.25	C21 H13 O4	15.5	0.25
MS7 (329.0810)	301.0864	CH3 + CH3 + CH3 + OH + CO + CO	118.0612	-1.74	C20 H13 O3	14.5	-0.11

Table S31 Detected MS^n fragments for LO 27

MS level (fragment)	Detected m/z	Lost fragment	Mass difference to $[M-H]^-$	Mass difference (mDa) of theoretical fragment mass - observed fragment mass	Determined elemental composition of detected mass	RDB	Mass difference (mDa) of theoretical mass - observed mass
MS1	421.1289	-	-	-	C24 H21 O7	14.5	0.73
MS2 (421.1289)	406.1047	CH3	15.0242	0.70	C23 H18 O7	15.0	0.04
	391.0812	CH3 + CH3	30.0471	0.11	C22 H15 O7	15.5	0.62
	389.1030	CH3 + OH	32.0259	-0.28	C23 H17 O6	15.5	1.01
	378.1105	CH3 + CO	43.0184	0.04	C22 H18 O6	14.0	0.68
MS3 (406.1047)	391.0812	CH3 + CH3	30.0477	0.72	C22 H15 O7	15.5	0.01
	388.0952	CH3 + H2O	33.0337	-0.38	C23 H16 O6	16.0	1.11
	378.1099	CH3 + CO	43.0190	0.62	C22 H18 O6	14.0	0.10
	363.0868	CH3 + CH3 + CO	58.0421	0.25	C21 H15 O6	14.5	0.48
MS3 (391.0812)	376.0581	CH3 + CH3 + CH3	45.0709	0.47	C21 H12 O7	16.0	0.39
	375.0502	CH3 + CH3 + CH4	46.0788	0.55	C21 H11 O7	16.5	0.30
	373.0709	CH3 + CH3 + H2O	48.0581	0.60	C22 H13 O6	16.5	0.26
	363.0867	CH3 + CH3 + CO	58.0424	0.50	C21 H15 O6	14.5	0.36
	239.0347	CH3 + CH3 + C8H8O3	182.0944	0.09	C14 H7 O4	11.5	0.77
	211.0399	CH3 + CH3 + C9H8O4	210.0892	-0.04	C13 H7 O3	10.5	0.89
MS4 (391.0814)	376.0581	CH3 + CH3 + CH3	45.0699	-0.58	C21 H12 O7	16.0	-0.23
	375.0503	CH3 + CH3 + CH4	46.0776	-0.64	C21 H11 O7	16.5	-0.16
	373.0711	CH3 + CH3 + H2O	48.0568	-0.73	C22 H13 O6	16.5	-0.07
	363.0866	CH3 + CH3 + CO	58.0413	-0.55	C21 H15 O6	14.5	-0.25
	239.0346	CH3 + CH3 + C8H8O3	182.0934	-0.94	C14 H7 O4	11.5	0.15
	211.0398	CH3 + CH3 + C9H8O4	210.0882	-1.04	C13 H7 O3	10.5	0.24

MS5 (363.0863)	348.0628	CH3 + CH3 + CO + CH3	73.0645	-0.80	C20 H12 O6	15.0	-0.58
MS6 (348.0626)	330.0519	CH3 + CH3 + CO + CH3 + H2O	91.0750	-0.90	C20 H10 O5	16.0	-0.91
	320.0680	CH3 + CH3 + CO + CH3 + CO	101.0589	-1.35	C19 H12 O5	14.0	-0.45

Table S32 Detected MS^n fragments for LO 28

MS level (fragment)	Detected m/z	Lost fragment	Mass difference to [M-H]$^-$	Mass difference (mDa) of theoretical fragment mass - observed fragment mass	Determined elemental composition of detected mass	RDB	Mass difference (mDa) of theoretical mass - observed mass
MS1	451.1756	-	-	-	C26 H27 O7	13.5	-0.11
MS2 (451.1756)	436.1515	CH3	15.0241	0.64	C25 H24 O7	14.0	-0.74
	421.1285	C2H6	30.0471	0.14	C24 H21 O7	14.5	-0.25
	419.1496	CH4O	32.0260	-0.25	C25 H23 O6	14.5	0.14
	418.1419	CH5O	33.0336	-0.41	C25 H22 O6	15.0	0.30
	405.1334	C2H6O	46.0421	0.28	C24 H21 O6	14.5	-0.39
MS3 (436.1515)	421.1279	CH3 + CH3	30.0477	0.75	C24 H21 O7	14.5	-0.86
	420.1202	CH3 + CH4	31.0554	0.59	C24 H20 O7	15.0	-0.69
	405.1330	CH3 + CH3O	46.0426	0.71	C24 H21 O6	14.5	-0.81
	403.1176	CH3 + CH5O	48.0580	0.50	C24 H19 O6	15.5	-0.61
	391.1176	CH3 + C2H5O	60.0580	0.44	C23 H19 O6	14.5	-0.55
	244.0735	CH3 + C11H12O3	207.1020	-0.08	C14 H12 O4	9.0	-0.03
	192.0789	CH3 + C14H12O4	259.0966	-0.40	C11 H12 O3	6.0	0.29

MS3 (405.1330)	390.1104	C2H6O + CH3	61.0650	-0.25	C23 H18 O6	15.0	0.04
	387.1233	C2H6O + H2O	64.0521	-0.37	C24 H19 O5	15.5	0.07
	375.0866	C2H6O + CH3 + CH3	76.0888	-0.04	C22 H15 O6	15.5	-0.25
MS4 (421.1279)	406.1048	CH3 + CH3 + CH3	45.0699	-0.58	C23 H18 O7	15.0	-0.45
	403.1177	CH3 + CH3 + H2O	48.0570	-0.54	C24 H19 O6	15.5	-0.48
	391.1177	CH3 + CH3 + CH2O	60.0569	-0.57	C23 H19 O6	14.5	-0.45
	362.0786	CH3 + CH3 + C3H7O	89.0961	-0.57	C21 H14 O6	15.0	-0.46
	241.0500	CH3 + CH3 + C10H12O3	210.1246	-0.10	C14 H9 O4	10.5	-0.03
MS4 (405.1330)	390.1095	C2H6O + CH3	61.0649	-0.40	C23 H18 O6	15.0	-0.84
	387.1225	C2H6O + H2O	64.0520	-0.46	C24 H19 O5	15.5	-0.78
MS5 (390.1093)	375.0858	C2H6O + CH3 + CH3	76.0882	-0.59	C22 H15 O6	15.5	-1.05
	230.0576	C2H6O + CH3 + C10H8O2	221.1165	-1.30	C13 H10 O4	9.0	-0.34
MS6 (375.0858)	357.0758	CH3 + CH2O + H + CH3 + CH3 + H2O	94.0983	-1.11	C22 H13 O5	16.5	-0.52
	227.0344	CH3 + CH2O + H + CH3 + CH3 + C9H8O2	224.1396	-1.64	C13 H7 O4	10.5	0.01

Table S33 Detected MS^n fragments for LO 29

MS level (fragment)	Detected m/z	Lost fragment	Mass difference to $[M-H]^-$	Mass difference (mDa) of theoretical fragment mass - observed fragment mass	Determined elemental composition of detected mass	RDB	Mass difference (mDa) of theoretical mass - observed mass
MS1	463.1756	-	-	-	C27 H27 O7	14.5	-0.08
MS2 (463.1756)	448.1513	CH3	15.0243	0.85	C26 H24 O7	15.0	-0.92
	433.1646	CH2O	30.0110	0.43	C26 H25 O6	14.5	-0.50
	297.1125	C9H10O3	166.0631	0.09	C18 H17 O4	10.5	-0.16
	270.0891	C11H13O3	193.0865	0.08	C16 H14 O4	10.0	-0.16
	269.0816	C11H14O3	194.0943	0.01	C16 H13 O4	10.5	-0.08
	255.0659	C12H16O3	208.1097	-0.04	C15 H11 O4	10.5	-0.06
	178.0634	C17H17O4	285.1122	-0.48	C10 H10 O3	6.0	0.41
	164.0478	C18H19O4	299.1278	-0.54	C9 H8 O3	6.0	0.47
MS3 (448.1213)	433.1287	CH3 + CH3	30.0469	-0.07	C25 H21 O7	15.5	-0.01
	270.0895	CH3 + C10H10O3	193.0861	-0.35	C16 H14 O4	10.0	0.27
	269.0816	CH3 + C10H11O3	194.0940	-0.27	C16 H13 O4	10.5	0.19
	255.0659	CH3 + C11H13O3	208.1097	-0.21	C15 H11 O4	10.5	0.14
	178.0635	CH3 + C16H14O4	285.1121	-0.56	C10 H10 O3	6.0	0.49
MS3 (433.1646)	418.1415	CH2O + CH3	45.0340	-0.04	C25 H22 O6	15.0	-0.13
	403.1183	CH2O + CH3 + CH3	60.0572	-0.36	C24 H19 O6	15.5	0.187
	297.1126	CH2O + C8H8O2	166.0629	-0.09	C18 H17 O4	10.5	-0.07
	282.0893	CH2O + C9H11O2	181.0862	-0.26	C17 H14 O4	11.0	0.08
MS4 (255.0659)	240.0424	CH3 + C11H13O3 + CH3	223.1319	-1.52	C14 H8 O4	11.0	0.16
	237.0554	CH3 + C11H13O3 + H2O	226.1189	-1.63	C15 H9 O3	11.5	0.27
	227.0709	CH3 + C11H13O3 + CO	236.1034	-1.42	C14 H11 O3	9.5	0.06

Table S34 Detected MS^n fragments for LO 30

MS level (fragment)	Detected m/z	Lost fragment	Mass difference to $[M-H]^-$	Mass difference (mDa) of theoretical fragment mass - observed fragment mass	Determined elemental composition of detected mass	RDB	Mass difference (mDa) of theoretical mass - observed mass
MS1	479.1711	-	-	-	C27 H27 O8	14.5	0.55
MS2 (479.1711)	464.1463	CH3	15.0248	1.37	C26 H24 O8	15.0	-0.82
	463.1388	CH4	16.0323	1.05	C26 H23 O8	15.5	-0.50
	461.1598	H2O	18.0113	0.76	C27 H25 O7	15.5	-0.21
	449.1233	C2H6	30.0479	0.93	C25 H21 O8	15.5	-0.38
	447.1441	CH4O	32.0249	0.83	C26 H23 O7	15.5	-0.28
	446.1362	CH5O	33.0349	0.87	C26 H22 O7	16.0	-0.32
	436.1520	C2H3O	43.0191	0.74	C25 H24 O7	14.0	-0.19
	433.1284	C2H6O	46.0428	0.90	C25 H21 O7	15.5	-0.50
	272.0685	C12H15O3	207.1026	0.48	C15 H12 O5	10.0	0.07
MS3 (464.1463)	449.1231	CH3 + CH3	30.0480	1.05	C25 H21 O8	15.5	-0.50
	448.1153	CH3 + CH4	31.0558	1.08	C25 H20 O8	16.0	-0.52
	436.1518	CH3 + CO	43.0194	0.99	C25 H24 O7	14.0	-0.44
	433.1282	CH3 + CH3O	46.0429	1.05	C25 H21 O7	15.5	-0.50
	406.1417	CH3 + C2H2O2	73.0294	0.47	C24 H22 O6	14.0	0.08
	272.0685	CH3 + C11H12O3	207.1027	0.57	C15 H12 O5	10.0	-0.02
	244.0737	CH3 + C12H12O4	235.0974	0.40	C14 H12 O4	9.0	0.16
MS3 (449.1233)	434.0997	CH3 + CH3 + CH3	45.0716	1.14	C24 H18 O8	16.0	-0.50
	431.1123	CH3 + CH3 + H2O	48.0590	1.45	C25 H19 O7	16.5	-0.81
	419.1127	CH3 + CH3 + CH2O	60.0585	1.02	C24 H19 O7	15.5	-0.38
	390.0733	CH3 + CH3 + C3H7O	89.0980	1.32	C22 H14 O7	16.0	-0.68
	269.0449	CH3 + CH3 + C10H12O3	210.1263	0.75	C15 H9 O5	11.5	-0.11
MS3 (433.1284)	418.1046	C2H6O + CH3	61.0659	0.52	C24 H18 O7	16.0	-0.61

	415.1177	C2H6O + H2O	64.0528	0.40	C25 H19 O6	16.5	-0.48
MS4 (449.1231)	434.0989	CH3 + CH3 + CH3	45.0699	-0.58	C24 H18 O8	16.0	-1.26
	431.1115	CH3 + CH3 + H2O	48.0573	-0.21	C25 H19 O7	16.5	-1.63
	419.1118	CH3 + CH3 + CH2O	60.0570	-0.54	C24 H19 O7	15.5	-1.29
	390.0723	CH3 + CH3 + C3H7O	89.0965	-0.17	C22 H14 O7	16.0	-1.65
	269.0435	CH3 + CH3 + C10H12O3	210.1252	-0.38	C15 H9 O5	11.5	-1.45
MS4 (433.1274)	418.1044	CH3 + CH3O + CH3	61.0649	-0.46	C24 H18 O7	16.0	-0.85
	415.1176	CH3 + CH3O + H2O	64.0517	-0.70	C25 H19 O6	16.5	-0.61
MS5 (418.1049)	390.1098	CH3 + CH3O + CH3 + CO	89.0594	-0.89	C23 H18 O6	15.0	-0.51

Table S35 Detected MS^n fragments for LO 31

MS level (fragment)	Detected m/z	Lost fragment	Mass difference to $[M-H]^-$	Mass difference (mDa) of theoretical fragment mass - observed fragment mass	Determined elemental composition of detected mass	RDB	Mass difference (mDa) of theoretical mass - observed mass
MS1	483.2010	-	-	-	C27 H31 O8	12.5	-0.66
MS2 (483.2012)	468.1778	CH3	15.0234	-0.04	C26 H28 O8	13.0	-0.62
	451.1752	CH3 + CO	32.0261	-0.16	C26 H27 O7	13.5	-0.51
	437.1600	C2H6O	46.0413	-0.61	C25 H25 O7	13.5	-0.06
	301.1076	C10H14O3	182.0936	-0.69	C17 H17 O5	9.5	0.04
	287.0919	C11H16O3	196.1093	-0.66	C16 H15 O5	9.5	0.00
	244.0737	C13H19O4	239.1275	-0.84	C14 H12 O4	9.0	0.19
MS3 (437.1600)	407.1497	C2H6O + CH2O	76.0515	-0.95	C24 H23 O6	13.5	0.29
	300.0998	C2H6O + C8H9O2	183.1014	-0.68	C17 H16 O5	10.0	0.02

	286.0841	C2H6O + C9H11O2	197.1171	-0.64	C16 H14 O5	10.0	-0.01
	283.0971	C2H6O + C8H10O3	200.1041	-0.73	C17 H15 O4	10.5	0.08
	269.0813	C2H6O + C9H12O3	214.1199	-0.61	C16 H13 O4	10.5	-0.05
	244.0736	C2H6O + C11H13O3	239.1276	-0.71	C14 H12 O4	9.0	0.05
	230.0581	C2H6O + C12H15O3	253.1431	-0.84	C13 H10 O4	9.0	0.18
MS3 (468.1775)	437.1599	CH3 + CH3O	46.0408	-1.10	C25 H25 O7	13.5	-0.18
	331.1180	CH3 + C8H9O2	152.0827	-1.06	C18 H19 O6	9.5	-0.21
	301.1076	CH3 + C9H11O3	182.0930	-1.30	C17 H17 O5	9.5	0.04
	287.0919	CH3 + C10H13O3	196.1087	-1.27	C16 H15 O5	9.5	0.00
	273.0764	CH3 + C11H15O3	210.1242	-1.41	C15 H13 O5	9.5	0.15
	257.0814	CH3 + C11H15O4	226.1192	-1.28	C15 H13 O4	9.5	0.01
	244.0737	CH3 + C12H16O4	239.1270	-1.38	C14 H12 O4	9.0	0.11
	230.0580	CH3 + C13H18O4	253.1426	-1.39	C13 H10 O4	9.0	0.12
MS4 (287.0919)	272.0688	CH3 + C10H13O3 + CH3	211.1319	-1.55	C15 H12 O5	10.0	0.29
MS5 (272.0689)	243.0663	CH3 + C10H13O3 + CH3 + CHO	240.1354	-0.72	C14 H11 O4	9.5	0.52

Table S36 Detected MS^n fragments for LO 32

MS level (fragment)	Detected m/z	Lost fragment	Mass difference to $[M-H]^-$	Mass difference (mDa) of theoretical fragment mass - observed fragment mass	Determined elemental composition of detected mass	RDB	Mass difference (mDa) of theoretical mass - observed mass
MS1	491.1704	-	-	-	C28 H27 O8	15.5	0.37
MS2 (491.1697)	476.1461	CH3	15.0244	0.88	C27 H24 O8	16.0	-1.06
	447.1800	CO2	43.9904	0.62	C27 H27 O6	14.5	-0.80
	432.1567	C2H3O2	59.0137	0.37	C26 H24 O6	15.0	-0.55
MS3 (476.1461)	461.1229	CH3 + CH3	30.0475	0.60	C26 H21 O8	16.5	-078
	432.1568	CH3 + CO2	59.0136	0.34	C26 H24 O6	15.0	-0.52
	417.1336	CH3 + C2H3O2	74.0368	0.05	C25 H21 O6	15.5	-0.23
	296.1049	CH3 + C9H8O4	195.0655	-0.24	C18 H16 O4	11.0	0.03
	271.0970	CH3 + C11H9O4	220.0734	-0.20	C16 H15 O4	9.5	0.02
MS3 (447.1800)	432.1566	CO2 + CH3	59.0132	-0.06	C26 H24 O6	15.0	-0.15
	296.1048	CO2 + C9H11O2	195.0650	-0.70	C18 H16 O4	11.0	0.49
MS4 (461.1229)	446.0981	CH3 + CH3 + CH3	45.0706	0.20	C25 H18 O8	17.0	-2.06
	443.1126	CH3 + CH3 + H20	48.0561	-1.43	C26 H19 O7	17.5	-0.44
	417.1324	CH3 + CH3 + CO2	74.0363	-0.43	C25 H21 O6	15.5	-1.42
	293.0812	CH3 + CH3 + C8H8O4	198.0875	-1.72	C18 H13 O4	12.5	-0.14
	255.0647	CH3 + CH3 + C11H10O4	236.1040	-0.84	C15 H11 O4	10.5	-1.02
MS4 (432.1574)	417.1341	CH3 + CO2 + CH3	74.0356	-1.14	C25 H21 O6	15.5	0.28
	310.1206	CH3 + CO2 + C7H6O2	181.0492	-0.92	C19 H18 O4	11.0	0.06

	296.1050	CH3 + CO2 + C8H8O2	195.0645	-1.03	C18 H16 O4	11.0	0.18
	295.0972	CH3 + CO2 + C8H9O2	196.0725	-1.05	C18 H15 O4	11.5	0.20
	281.0816	CH3 + CO2 + C9H11O2	210.0881	-1.08	C17 H13 O4	11.5	0.22
	269.0817	CH3 + CO2 + C10H11O2	222.0881	-1.14	C16 H13 O4	10.5	0.29
MS5 (417.1337)	402.1114	CH3 + CH3 + CO2 + CH3	89.0582	-2.05	C24 H18 O6	16.0	1.00
	293.0814	CH3 + CH3 + CO2 + C7H8O2	198.0882	-1.039	C18 H13 O4	12.5	0.006

Table S37 Detected MS^n fragments for LO 33

MS level (fragment)	Detected m/z	Lost fragment	Mass difference to $[M-H]^-$	Mass difference (mDa) of theoretical fragment mass - observed fragment mass	Determined elemental composition of detected mass	RDB	Mass difference (mDa) of theoretical mass - observed mass
MS1	507.1649	-	-	-	C28 H27 O9	15.5	1.03
MS2 (507.1649)	492.1410	CH3	15.0250	1.52	C27 H24 O9	16.0	-0.49
	491.1337	CH4	16.0323	0.99	C27 H23 O9	16.5	0.07
	477.1182	C2H6	30.0478	1.30	C26 H21 O9	16.5	-0.33
	475.1389	CH4O	32.0271	0.86	C27 H23 O8	16.5	-0.37
	464.1469	C2H3O	43.0191	0.71	C26 H24 O8	15.0	-0.24
MS3 (492.1410)	491.1337	CH3 + H	16.0323	0.99	C27 H23 O9	16.5	-0.51
	476.1098	CH3 + CH4	31.0562	1.38	C26 H20 O9	17.0	-0.35
	475.1385	CH3 + OH	32.0275	1.32	C27 H23 O8	16.5	-0.28
	474.1306	CH3 + H2O	33.0353	1.30	C27 H22 O8	17.0	-0.27
	473.1230	CH3 + H2O + H	34.0430	1.14	C27 H21 O8	17.5	-0.10
	464.1465	CH3 + CO	43.0195	1.08	C26 H24 O8	15.0	-0.06
	463.1385	CH3 + CHO	44.0275	1.29	C26 H23 O8	15.5	-0.25
	461.1235	CH3 + CH3O	46.0425	0.65	C26 H21 O8	16.5	0.38
	448.1153	CH3 + C2H4O	59.0507	0.97	C25 H20 O8	16.0	0.06
	431.1129	CH3 + C2H5O2	76.0531	0.64	C25 H19 O7	16.5	0.39
	420.1204	CH3 + C3H4O2	87.0456	1.02	C24 H20 O7	15.0	0.01
	327.0866	CH3 + C9H9O3	180.0793	0.71	C18 H15 O6	11.5	0.33
	313.0710	CH3 + C10H11O3	194.0950	0.68	C17 H13 O6	11.5	0.35
	301.0710	CH3 + C11H15O3	206.0950	0.68	C16 H13 O6	10.5	0.35
	287.0554	CH3 + C12H13O3	220.1106	0.66	C15 H11 O6	10.5	0.38
MS3 (477.1182)	462.0950	CH3 + CH3 + CH3	45.0705	0.11	C25 H18 O9	17.0	0.44

	459.1075	CH3 + CH3 + H2O	48.0580	0.50	C26 H19 O8	17.5	0.04
	449.1231	CH3 + CH3 + CO	58.0424	0.53	C25 H21 O8	15.5	0.02
	447.1076	CH3 + CH3 + CH2O	60.0579	0.38	C25 H19 O8	16.5	0.16
	433.1288	CH3 + CH3 + CO2	74.0367	-0.10	C25 H21 O7	14.5	-0.24
	242.0215	CH3 + CH3 + C13H15O4	265.1440	-0.03	C13 H6 O5	11.0	-0.29
MS4 (474.1299)	459.1072	CH3 + H2O + CH3	48.0575	-0.02	C26 H19 O8	17.5	-0.81
	446.1350	CH3 + H2O + CO	61.0297	0.74	C26 H22 O7	16.0	-1.57
	445.1282	CH3 + H2O + CHO	62.0365	-0.31	C26 H21 O7	16.5	-0.53
	418.1411	CH3 + H2O + C2O2	89.0236	-0.25	C25 H22 O6	15.0	-0.59
MS4 (464.1466)	446.1417	CH3 + CO + H2O	61.0283	-0.63	C26 H22 O7	16.0	-0.11
	299.0922	CH3 + CO + C9H9O3	208.0726	-0.96	C17 H15 O5	10.5	0.21
MS5 (418.1417)	269.0814	CH3 + H2O + C2O2 + C9H9O2	238.0834	-0.75	C16 H13 O4	10.5	0.01
MS5 (446.1356)	431.1125	CH3 + CO + H2O + CH3	76.0515	-0.98	C25 H19 O7	16.5	-0.56

Table S38 Detected MS^n fragments for LO 34

MS level (fragment)	Detected m/z	Lost fragment	Mass difference to $[M-H]^-$	Mass difference (mDa) of theoretical fragment mass - observed fragment mass	Determined elemental composition of detected mass	RDB	Mass difference (mDa) of theoretical mass - observed mass
MS1	509.2166	-	-	-	C29 H33 O8	13.5	-0.25
MS2 (509.2166)	494.1931	CH3	15.0242	0.76	C28 H30 O8	14.0	-1.02
MS3 (494.1931)	479.1699	CH3 + CH3	30.0474	0.47	C27 H27 O8	14.5	-0.73
	463.1750	CH3 + CH3O	46.0423	0.47	C27 H27 O7	14.5	-0.72
	449.1595	CH3 + C2H5O	60.0578	0.29	C26 H25 O7	14.5	-0.55
	385.1653	CH3 + C6H5O2	124.0520	0.46	C22 H25 O6	10.5	0.20
	327.1233	CH3 + C9H11O3	182.0940	-0.27	C19 H19 O5	10.5	0.01
	313.1076	CH3 + C10H13O3	196.1097	-0.26	C18 H17 O5	10.5	0.00
	295.0971	CH3 + C10H15O4	214.1202	-0.33	C18 H15 O4	11.5	0.08
	283.0971	CH3 + C11H15O4	226.1202	-0.23	C17 H15 O4	10.5	0.05
	281.0815	CH3 + C11H17O4	228.1358	-0.33	C17 H13 O4	11.5	0.07
	269.0815	CH3 + C12H17O4	240.1358	-0.36	C16 H13 O4	10.5	0.10
	255.0658	CH3 + C13H19O4	254.1514	-0.37	C15 H11 O4	10.5	0.11
MS4 (479.1699)	464.1462	CH3 + CH3 + CH3	45.0415	-0.18	C26 H24 O8	15.0	-0.88
	461.1585	CH3 + CH3 + H2O	48.0580	0.44	C27 H25 O7	15.5	-1.49
	449.1588	CH3 + CH3 + CH2O	60.0577	0.17	C26 H25 O7	14.5	-1.22
	311.0911	CH3 + CH3 + C9H12O3	198.1254	-0.16	C18 H15 O5	11.5	-0.89
	254.0575	CH3 + CH3 + C12H17O4	255.1590	-0.63	C15 H10 O4	11.0	-0.41

Table S39 Detected MS^n fragments for LO 35

MS level (fragment)	Detected m/z	Lost fragment	Mass difference to $[M\text{-}H]^-$	Mass difference (mDa) of theoretical fragment mass - observed fragment mass	Determined elemental composition of detected mass	RDB	Mass difference (mDa) of theoretical mass - observed mass
MS1	627.2230	-	-	-	C36 H35 O10	19.5	0.00
MS2 (627.2230)	612.1972	CH3	15.0245	1.07	C35 H32 O10	20.0	-1.80
	597.2105	CH2O	30.0115	0.98	C35 H33 O9	19.5	-1.70
	579.2004	CH4O2	48.0214	0.23	C35 H31 O8	20.5	-1.51
	567.2004	C2H4O2	60.0213	0.17	C34 H31 O8	19.5	-1.42
MS3 (612.1976)	597.1744	CH3 + CH3	30.0486	1.63	C34 H29 O10	20.5	-1.63
	594.1876	CH3 + H2O	33.0354	1.36	C35 H30 O9	21.0	-1.36
	460.1511	CH3 + C8H8O3	167.0719	1.11	C27 H24 O7	16.0	-1.11
	432.1201	CH3 + C10H12O3	195.1029	0.82	C25 H20 O7	16.0	-0.81
	406.1045	CH3 + C12H14O3	221.1185	0.76	C23 H18 O7	15.0	-0.76
	392.1253	CH3 + C12H12O4	235.0977	0.69	C23 H20 O6	14.0	-0.68
MS3 (579.2004)	564.1771	CH2O + H2O + CH3	63.0446	0.02	C34 H28 O8	21.0	-1.30
	547.1749	CH2O + H2O + CH4O	80.0469	0.46	C34 H27 O7	21.5	-0.81
MS4 (597.1755)	582.1500	CH3 + CH3 + CH3	45.0700	-0.25	C33 H26 O10	21.0	-0.99
	579.1650	CH3 + CH3 + H2O	48.0566	-0.85	C34 H27 O9	21.5	-0.53
	567.1653	CH3 + CH3 + CH2O	60.0563	-1.25	C33 H27 O9	20.5	-0.17
	549.1538	CH3 + CH3 + CH4O2	78.0678	-0.27	C33 H25 O8	21.5	-1.13
	537.1548	CH3 + CH3 + C2H4O2	90.668	-1.25	C32 H25 O8	20.5	-0.15
	375.0870	CH3 + CH3 + C12H14O4	252.1346	-1.58	C22 H15 O6	15.5	0.18
MS5 (567.1653)	372.0630	CH3 + CH3 + CH2O + C11H15O3	255.1587	-0.97	C22 H12 O6	17.0	-0.43

Table S40 Detected MS^n fragments for LO 36

MS level (fragment)	Detected m/z	Lost fragment	Mass difference to $[M-H]^-$	Mass difference (mDa) of theoretical fragment mass - observed fragment mass	Determined elemental composition of detected mass	RDB	Mass difference (mDa) of theoretical mass - observed mass
MS1	631.2539	-	-	-	C36 H39 O10	17.5	-0.41
MS2 (631.2539)	616.2289	CH3	15.0250	1.55	C35 H36 O10	18.0	-1.42
	599.2263	CH4O	32.0276	1.38	C35 H35 O9	18.5	-1.24
	585.2118	C2H6O	46.0427	0.25	C34 H33 O9	18.5	-0.12
	499.1597	C10H14O3	182.0942	-0.05	C26 H25 O7	14.5	0.19
	435.1439	C11H16O3	196.1100	0.08	C25 H23 O7	14.5	-0.98
MS3 (616.2289)	601.2055	CH3 + CH3	30.0484	1.45	C34 H33 O10	18.5	-1.31
	585.2112	CH3 + CH3O	46.0427	0.80	C34 H33 O9	18.5	-0.67
	571.1962	CH3 + CH3 + H2O	60.0577	0.23	C33 H31 O9	18.5	-0.64
	449.1593	CH3 + C9H11O3	182.0946	0.28	C26 H25 O7	14.5	-0.15
	435.1434	CH3 + C10H13O3	196.1105	0.56	C25 H23 O7	14.5	-0.43
	417.1332	CH3 + C10H15O4	214.1207	0.16	C25 H21 O6	15.5	-0.03
	405.1335	CH3 + C11H15O4	226.1204	-0.12	C24 H21 O6	14.5	0.25
MS3 (601.2055)	586.1821	CH3 + CH3 + CH3	45.0717	1.24	C33 H30 O10	19.0	-1.28
MS4 (601.2050)	586.1825	CH3 + CH3 + CH3	45.0699	-0.48	C33 H30 O10	19.0	-1.40
	583.1954	CH3 + CH3 + H2O	48.0571	-0.45	C34 H31 O9	19.5	-1.44

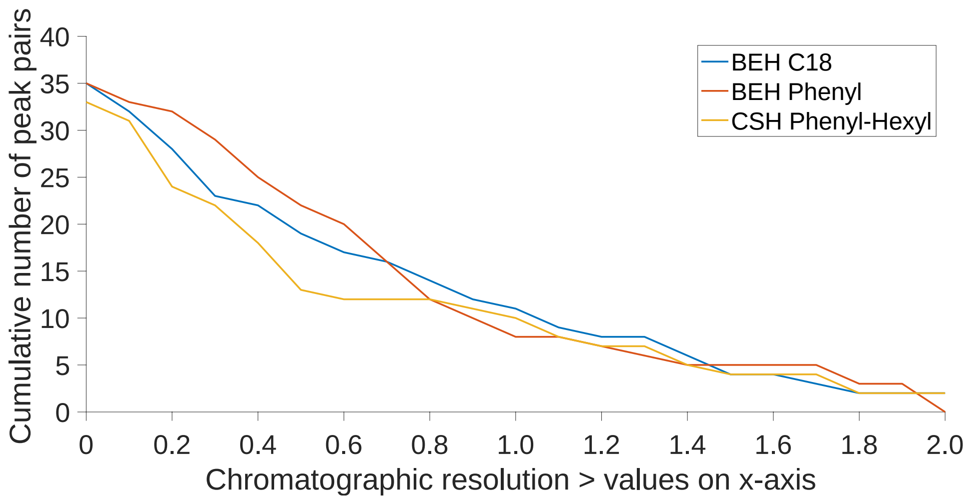

Fig. S2 Resolution level graph from the column screening

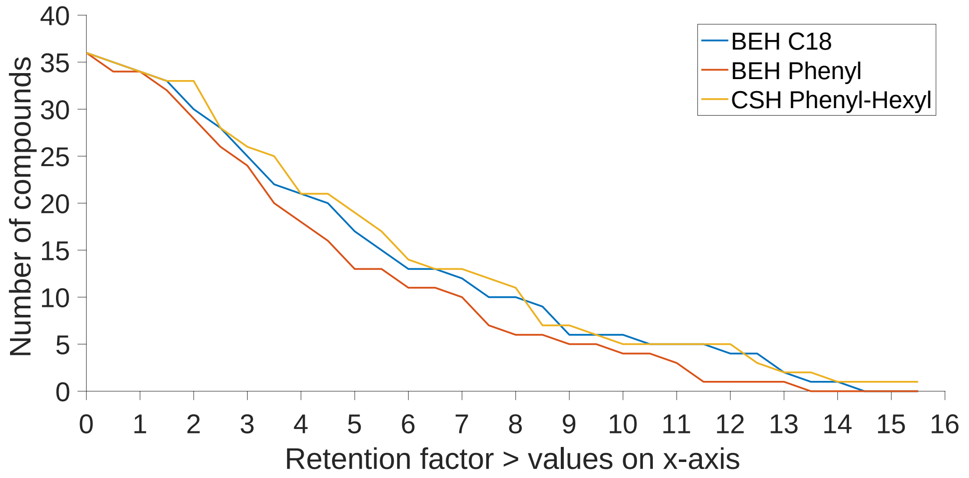

Fig. S3 Retention factor graph from the column screening

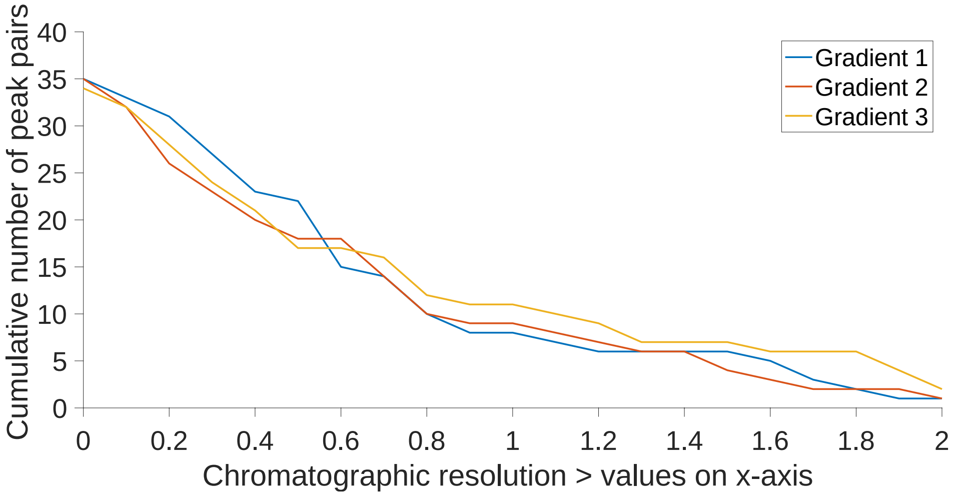

Fig. S4 Resolution level graph from the gradient optimisation

Table S41 Explained variance, cross-validated predictability, model validity and reproducibility of the full factorial design (2^3+3) used to investigate the influence of the capillary voltage, the sheath gas flow rate and the auxiliary gas flow rate on the base peak intensity of the identified lignin oligomers

Compound label	Measured m/z	Explained variance (R^2)	Cross-validated predictability (Q^2)	Model validity	Reproducibility
1	243.0664	0.87	0.60	0.23	0.98
2	245.0819	0.90	0.62	-0.20	1.00
3	257.0819	0.96	0.64	0.45	0.98
4	259.0971	0.91	0.65	-0.20	1.00
5	269.0816	0.95	0.63	0.51	0.98
6	271.0975	0.96	0.66	0.44	0.99
7	273.0768	0.90	0.64	0.07	0.99
8	273.1128	0.88	0.62	0.14	0.99
9	287.0926	0.96	0.72	0.45	0.99
10	299.0918	0.97	0.66	0.72	0.96
11	301.0717	0.75	0.27	-0.20	0.99
12	313.1079	0.90	0.65	0.49	0.97
13	315.1236	0.92	0.67	0.46	0.98
14	321.1131	0.84	0.50	0.66	0.89
15	335.0922	0.95	0.67	0.87	0.88
16	349.1077	0.90	0.54	0.64	0.92
17	351.1235	0.83	0.29	0.37	0.95
18	353.1391	0.95	0.60	0.32	0.99
19	357.1339	0.97	0.63	0.33	0.99
20	361.1652	0.95	0.73	0.42	0.99
21	373.1288	0.58	0.11	-0.20	1.00
22	377.1387	0.91	0.55	0.76	0.87
23	393.1332	0.86	0.60	0.66	0.90
24	395.1132	0.92	0.75	0.53	0.97
25	395.1494	0.94	0.59	0.56	0.96
26	419.1495	0.89	0.63	0.86	0.80
27	421.1289	0.99	0.72	0.69	0.99
28	451.1756	0.89	0.64	0.75	0.88
29	463.1756	0.94	0.61	0.84	0.88
30	479.1711	0.99	0.70	0.70	0.99
31	483.2010	0.99	0.65	0.77	0.98
32	491.1704	0.89	0.64	0.82	0.85
33	507.1649	0.98	0.67	0.92	0.95
34	509.2166	0.98	0.63	0.60	0.99
35	627.2230	0.90	0.66	0.84	0.84
36	631.2539	0.92	0.68	0.87	0.84

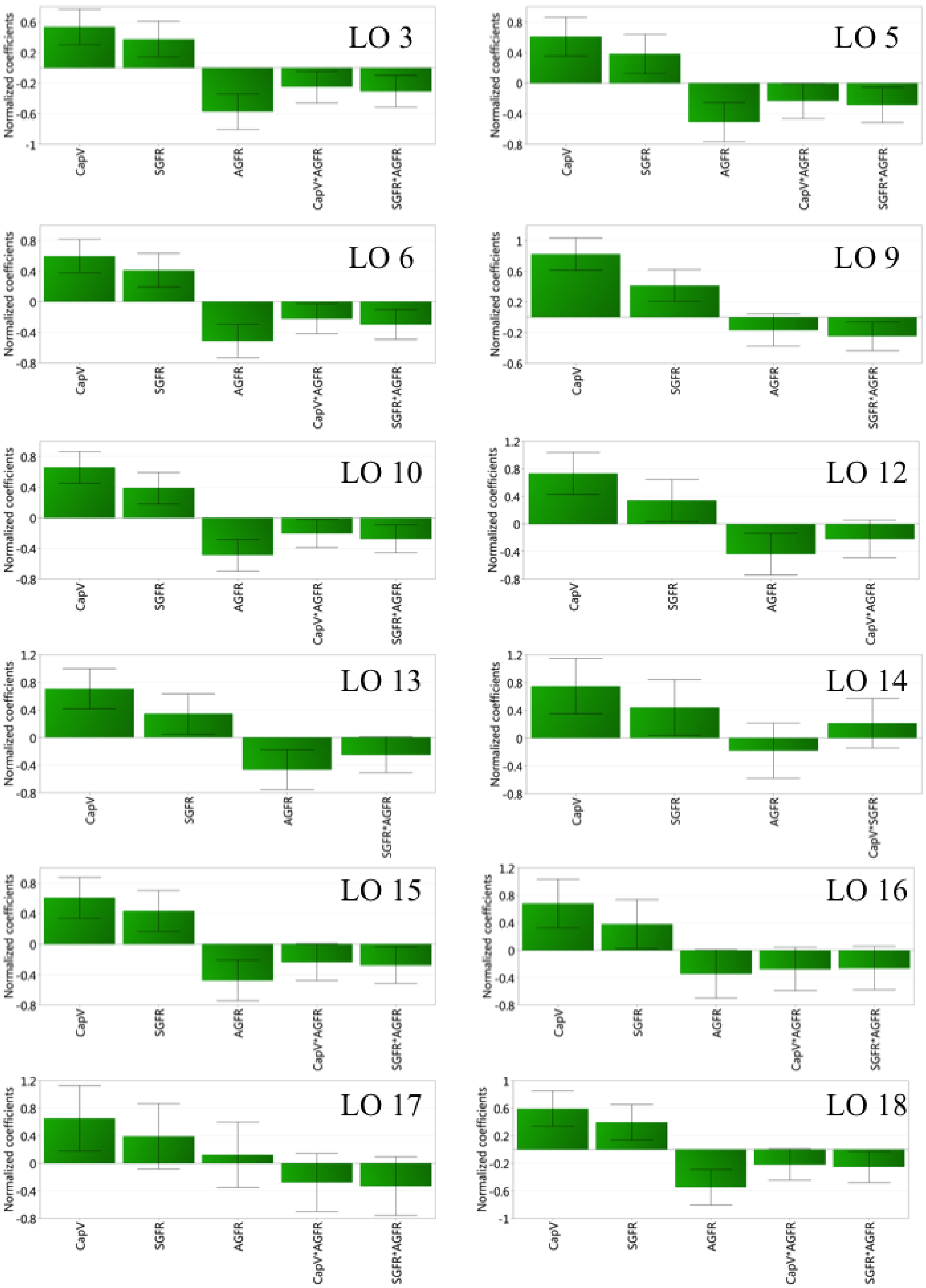

Fig. S5 Normalized coefficient plots of the identified LOs from the first design of experiment for the optimization of the MS ionization efficiency (Table S2). CapV: capillary voltage, SGFR: sheath gas flow rate, AGFR: auxiliary gas flow rate (Continuation on the next page)

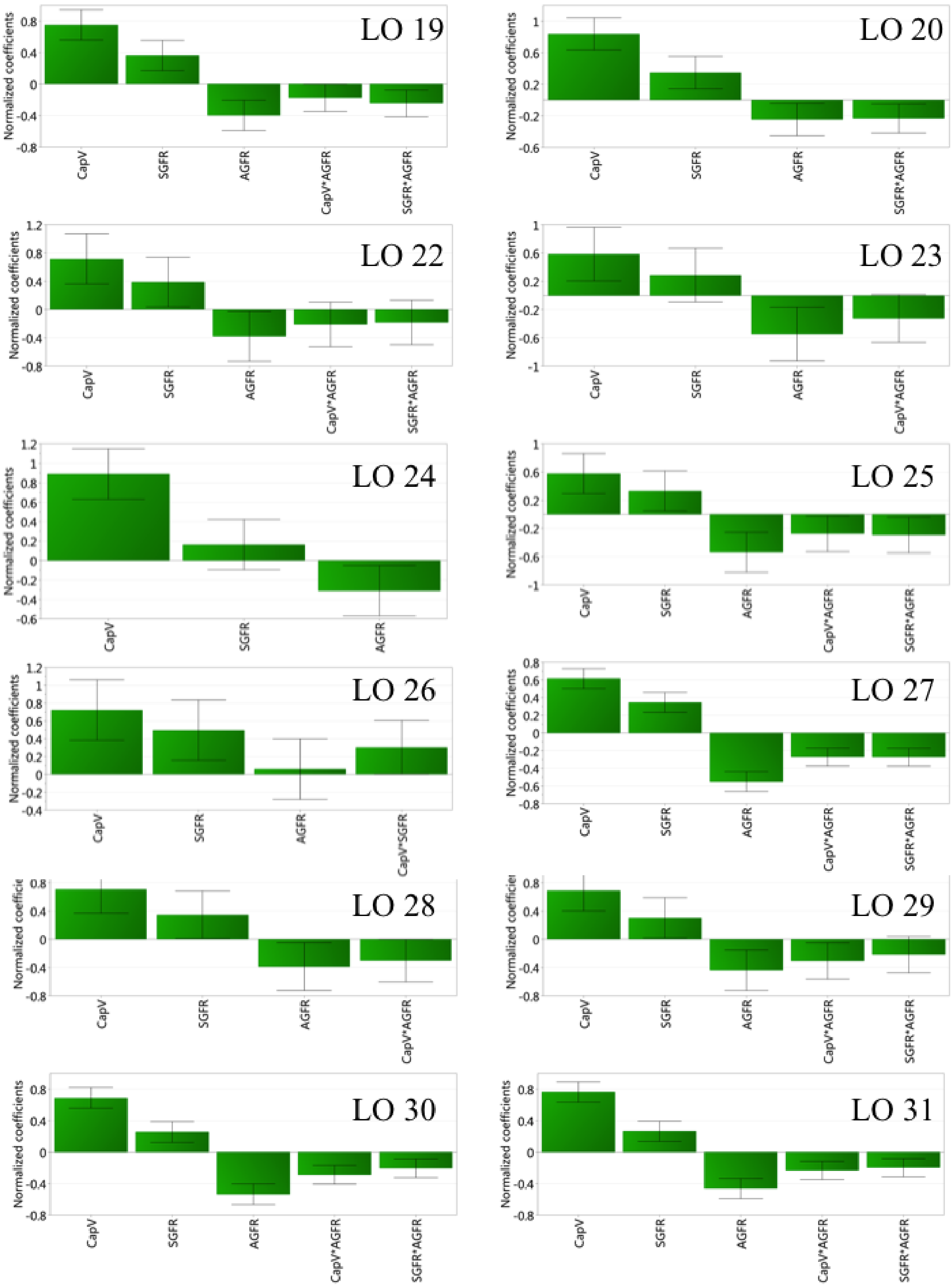

Fig. S5 (continued) Normalized coefficient plots of the identified LOs from the first design of experiment for the optimization of the MS ionization efficiency (Table S2). CapV: capillary voltage, SGFR: sheath gas flow rate, AGFR: auxiliary gas flow rate. (Continuation on the next page)

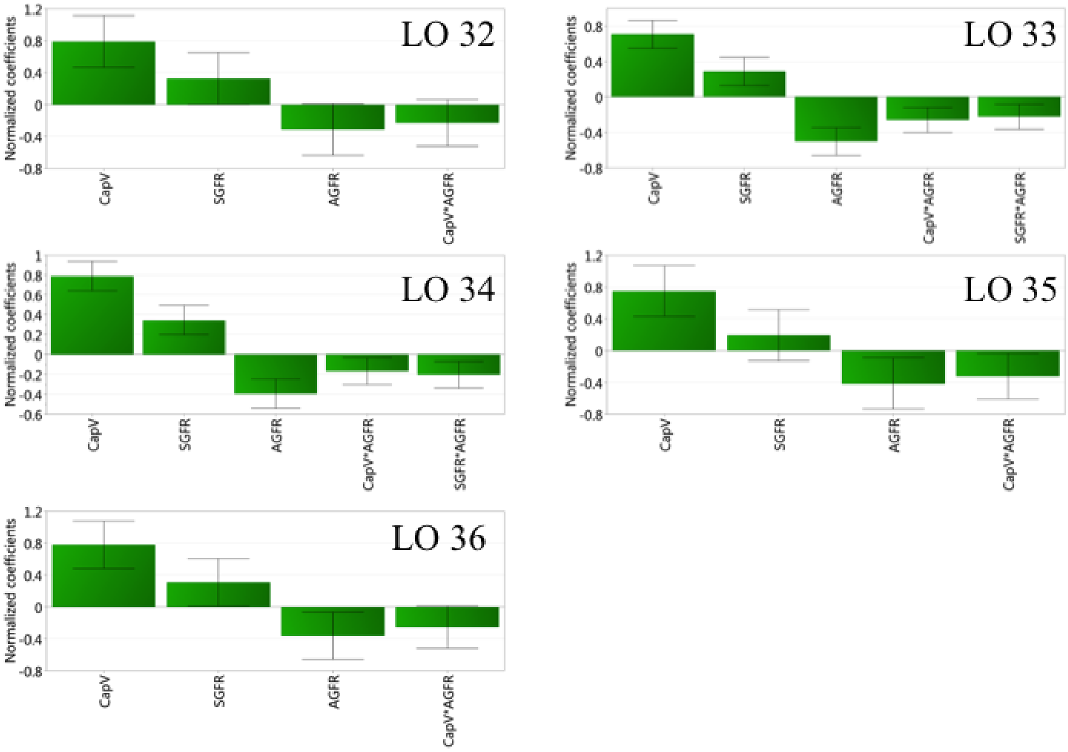

Fig. S5 (continued) Normalized coefficient plots of the identified LOs from the first design of experiment for the optimization of the MS ionization efficiency (Table S2). CapV: capillary voltage, SGFR: sheath gas flow rate, AGFR: auxiliary gas flow rate

Table S42 Explained variance, cross-validated predictability, model validity and reproducibility of the full factorial design (2^2+3) used to examine the impact of the sheath gas flow rate and the auxiliary gas flow rate on the base peak intensity of the identified lignin oligomers

Compound label	Measured m/z	Explained variance (R^2)	Cross-validated predictability (Q^2)	Model validity	Reproducibility
1	243.0664	0.97	0.56	0.60	0.97
2	245.0819	0.96	0.41	0.71	0.93
3	257.0819	0.97	0.47	0.88	0.91
4	259.0971	0.91	0.37	0.60	0.90
5	269.0816	0.95	0.43	0.68	0.93
6	271.0975	0.93	0.42	0.52	0.95
7	273.0768	0.98	0.48	0.74	0.96
8	273.1128	0.94	0.42	0.90	0.84
9	287.0926	0.92	0.34	0.49	0.94
10	299.0918	0.95	0.41	0.63	0.94
11	301.0717	0.96	0.43	0.49	0.97
12	313.1079	0.96	0.50	0.50	0.97
13	315.1236	0.96	0.51	0.67	0.96
14	321.1131	0.92	0.56	0.86	0.80
15	335.0922	0.96	0.55	0.09	0.99
16	349.1077	0.94	0.49	0.82	0.86
17	351.1235	0.97	0.58	0.78	0.94
18	353.1391	0.98	0.44	0.41	0.99
19	357.1339	0.93	0.50	0.68	0.90
20	361.1652	0.96	0.69	0.79	0.93
21	373.1288	0.96	0.59	0.72	0.94
22	377.1387	0.97	0.60	0.64	0.96
23	393.1332	0.95	0.49	0.77	0.90
24	395.1132	099	0.97	0.52	1.00
25	395.1494	0.89	0.19	-0.2	1.00
26	419.1495	0.97	0.80	0.69	0.96
27	421.1289	0.94	0.41	-0.2	1.00
28	451.1756	0.97	0.50	0.38	0.98
29	463.1756	0.88	0.48	0.92	0.65
30	479.1711	0.93	0.42	0.07	0.99
31	483.2010	0.93	0.38	0.54	0.94
32	491.1704	0.95	0.49	0.53	0.96
33	507.1649	0.96	0.44	0.66	0.94
34	509.2166	0.93	0.43	0.25	0.98
35	627.2230	0.95	0.56	0.77	0.91
36	631.2539	0.95	0.53	0.09	0.99

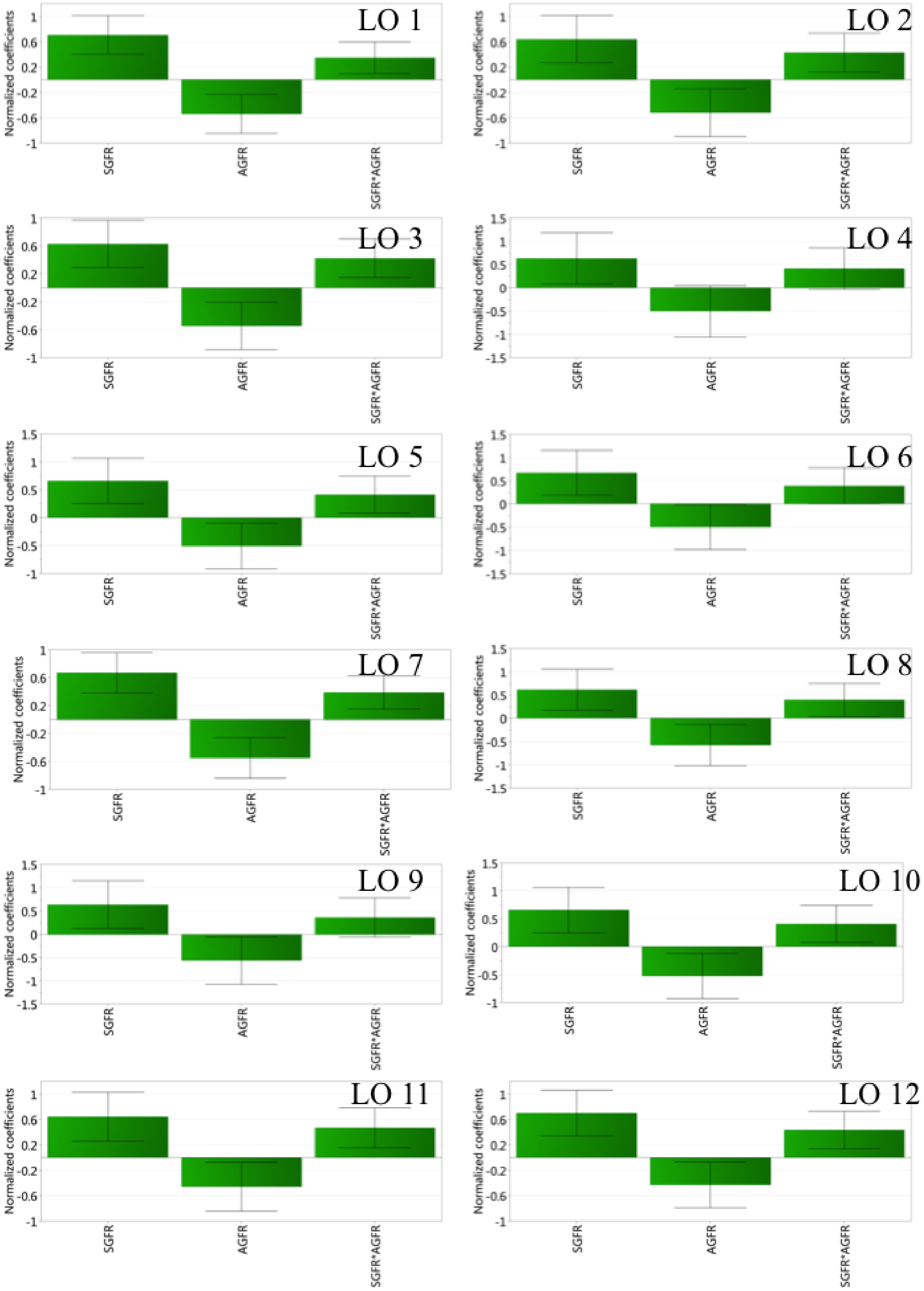

Fig. S6 Normalized coefficient plots of the identified LOs from the second design of experiment for the optimization of the MS ionization efficiency (Table S3). SGFR: sheath gas flow rate, AGFR: auxiliary gas flow rate. (Continuation on the next page)

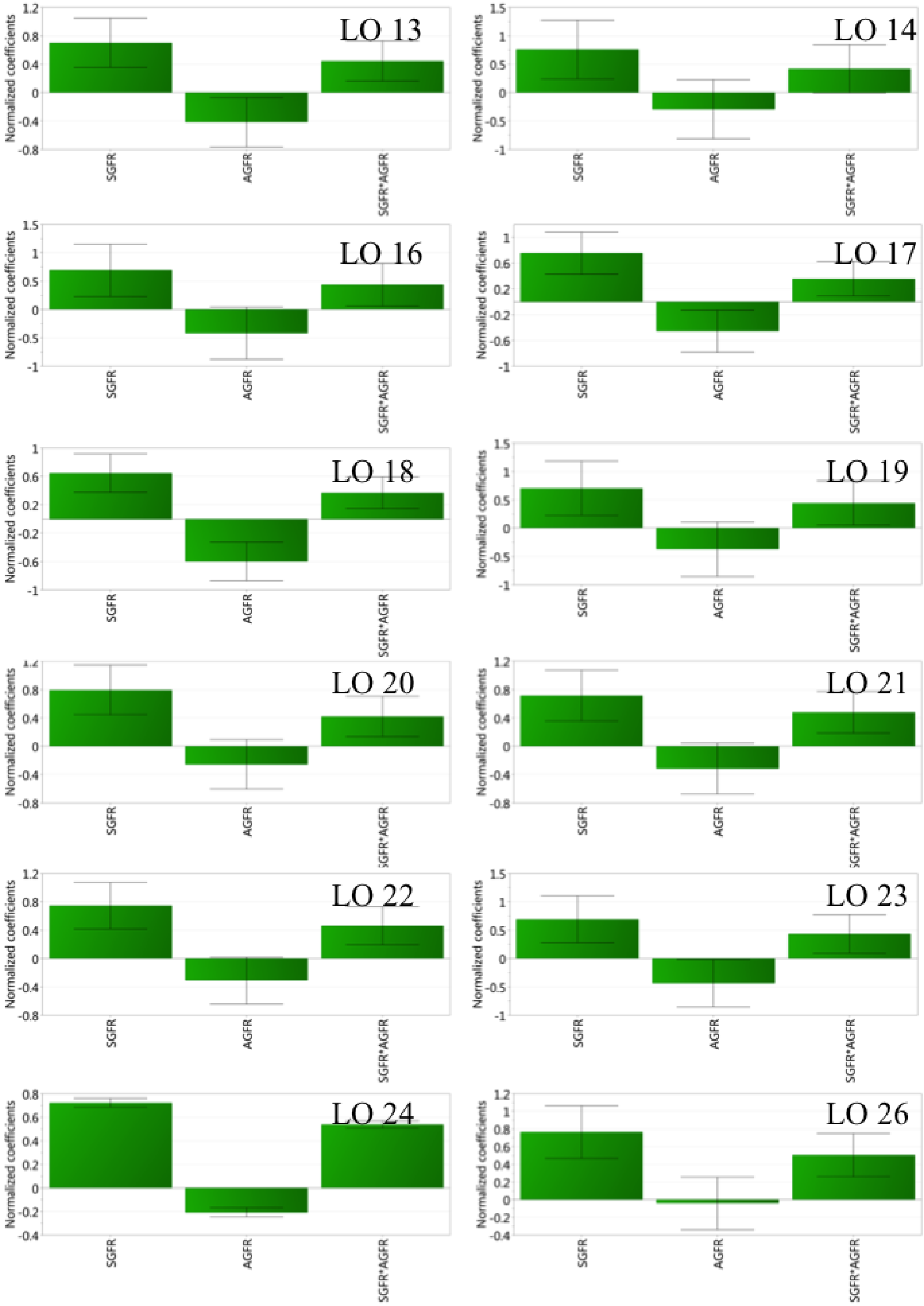

Fig. S6 (continued) Normalized coefficient plots of the identified LOs from the second design of experiment for the optimization of the MS ionization efficiency (Table S3). SGFR: sheath gas flow rate, AGFR: auxiliary gas flow rate. (Continuation on the next page)

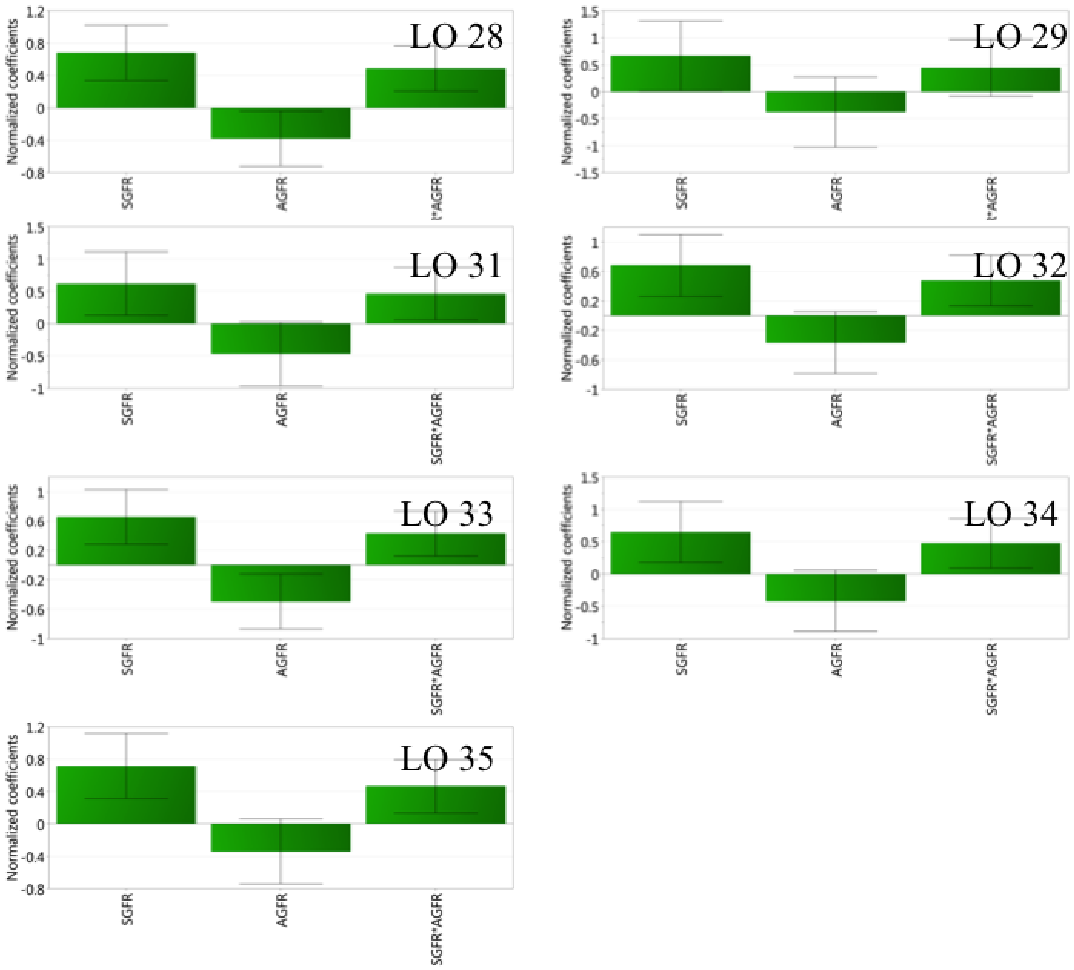

Fig. S6 (continued). Normalized coefficient plots of the identified LOs from the second design of experiment for the optimization of the MS ionization efficiency (Table S3). SGFR: sheath gas flow rate, AGFR: auxiliary gas flow rate

Table S43 Average base peak intensities and standard deviations of the identified LOs in the Kraft lignin samples using the preliminary LC/MS method and the optimised LC/MS method. Averages calculated from n=3. Data are compared using the heteroscedastic t-test

Compound label	Measured m/z	Average base peak intensity in preliminary LC/MS method	Standard deviation (preliminary LC/MS method)	Average base peak intensity in optimised LC/MS method	Standard deviation (optimised LC/MS method)	p-value
1	243.0664	383062	74053	425629	30211	0.433
2	245.0819	537858	57484	784835	25430	0.008
3	257.0819	204404	35651	323501	11603	0.020
4	259.0971	2262362	95226	3038870	119932	0.001
5	269.0816	414214	18622	631922	24512	0.000
6	271.0975	304125	25363	430797	23975	0.003
7	273.0768	1409000	45403	2253729	139939	0.005
8	273.1128	656012	69771	1003649	55647	0.003
9	287.0926	107988	9253	531045	25433	0.000
10	299.0918	2666826	196702	4293313	178961	0.000
11	301.0717	1070483	56821	2106749	204650	0.009
12	313.1079	599379	68204	833642	36772	0.013
13	315.1236	1031993	152551	1585917	60972	0.014
14	321.1131	266417	30016	277652	20694	0.625
15	335.0922	271790	27945	35319	5119	0.004
16	349.1077	97292	16020	81478	5566	0.223
17	351.1235	151809	25675	158438	13847	0.720
18	353.1391	98262	13941	172939	10376	0.002
19	357.1339	271733	31917	338571	9336	0.058
20	361.1652	4638726	255547	5063341	165137	0.084
21	373.1288	1503539	145948	1653639	75722	0.212
22	377.1387	144571	23556	137430	2802	0.653

23	393.1332	156261	21669	168793	11996	0.443
24	395.1132	820680	76116	1612952	151385	0.004
25	395.1494	59633	10435	75854	2964	0.105
26	419.1495	297347	33257	582529	23111	0.000
27	421.1289	1515857	126249	4112378	140228	0.000
28	451.1756	105316	2160	118685	4539	0.021
29	463.1756	158999	4332	166428	6424	0.182
30	479.1711	250852	8583	505365	15450	0.000
31	483.2010	249595	10277	320378	5174	0.002
32	491.1704	372727	1670	437720	25514	0.047
33	507.1649	507285	1127	775837	24858	0.003
34	509.2166	1339066	74524	1829520	130240	0.009
35	627.2230	172931	20281	233090	10072	0.020
36	631.2539	317705	11915	425593	13228	0.000

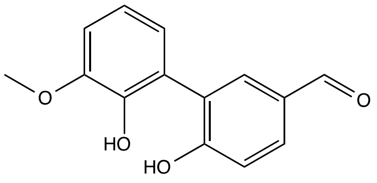

LO 1, m/z 244.0742,
$C_{14}H_{12}O_4$, RDB 9

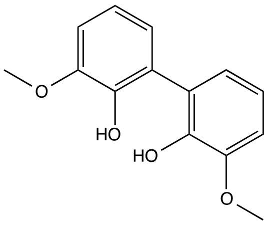

LO 2, m/z 246.0897,
$C_{14}H_{15}O_4$, RDB 8

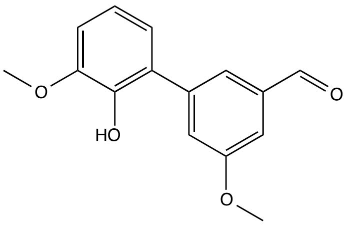

LO 3, m/z 258.0897,
$C_{15}H_{14}O_4$, RDB 9

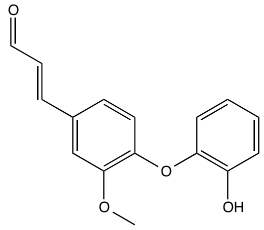

LO 5, m/z 270.0894,
$C_{16}H_{14}O_4$, RDB 10

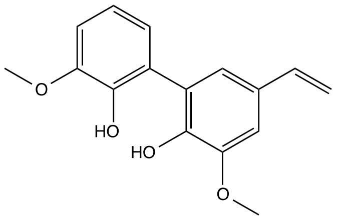

LO 6, m/z 272.1053,
$C_{16}H_{16}O_4$, RDB 9

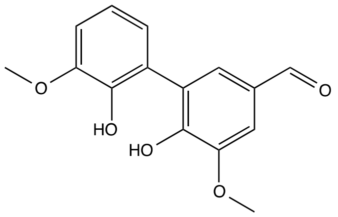

LO 7, m/z 274.0846,
$C_{15}H_{14}O_5$, RDB 9

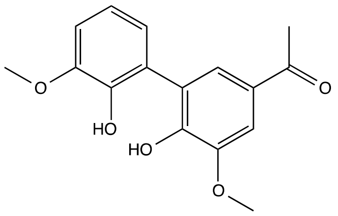

LO 9, m/z 288.1004,
$C_{16}H_{16}O_5$, RDB 9

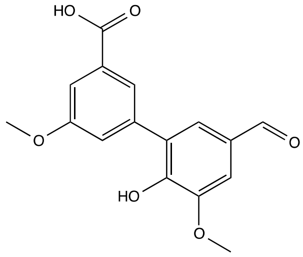

LO 11, m/z 302.0795,
$C_{16}H_{14}O_6$, RDB 10

Fig. S7 Tentative structures of identified lignin oligomers in the Kraft lignin sample. RDB: ring double bond equivalent. (Continuation on the next page)

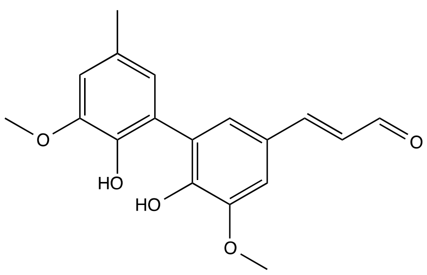

LO 12, m/z 314.1157, $C_{18}H_{18}O_5$, RDB 10

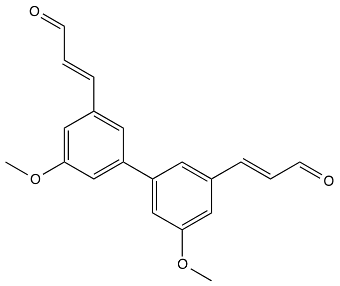

LO 14, m/z 322.1209, $C_{20}H_{18}O_4$, RDB 12

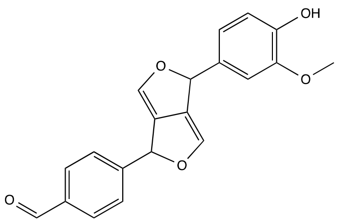

LO 15, m/z 336.1000, $C_{20}H_{16}O_5$, RDB 13

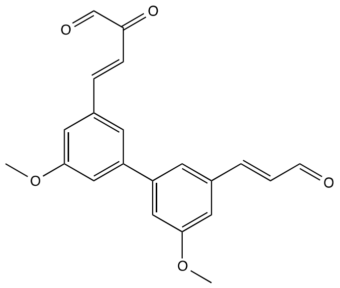

LO 16, m/z 350.1155, $C_{21}H_{18}O_5$, RDB 13

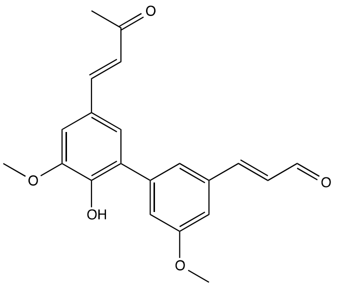

LO 17, m/z 351.1313, $C_{21}H_{20}O_5$, RDB 12

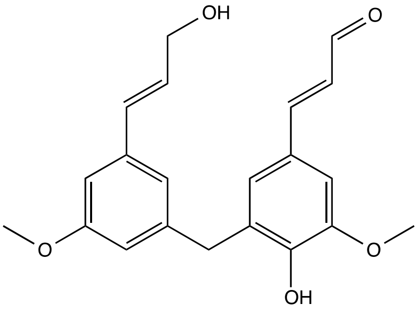

LO 18, m/z 354.1469, $C_{21}H_{22}O_5$, RDB 11

Fig. S7 (continued) Tentative structures of identified lignin oligomers in the Kraft lignin sample. RDB: ring double bond equivalent. (Continuation on the next page)

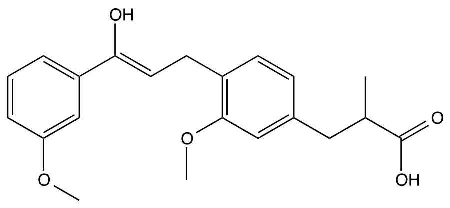

LO 19, m/z 358.1417, $C_{20}H_{26}O_6$, RDB 10

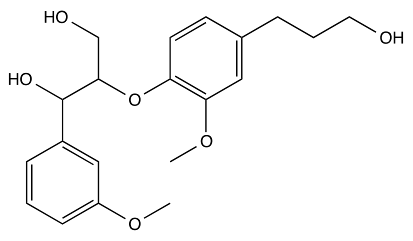

LO 20, m/z 362.1730, $C_{20}H_{26}O_6$, RDB 8

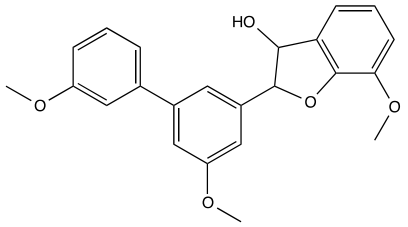

LO 22, m/z 378.1365, $C_{23}H_{22}O_5$, RDB 13

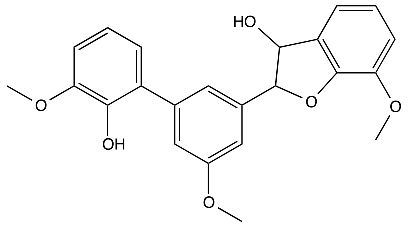

LO 23, m/z 394.1410, $C_{23}H_{22}O_6$, RDB 13

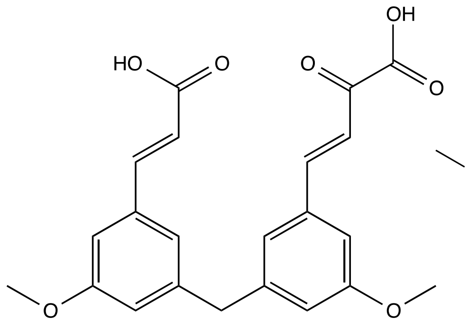

LO 24, m/z 396.1210, $C_{22}H_{19}O_7$, RDB 13

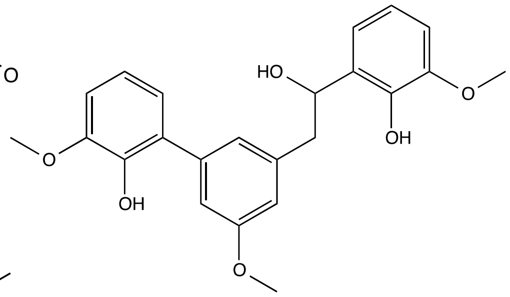

LO 25, m/z 396.1572, $C_{23}H_{24}O_6$, RDB 12

Fig. S7 (continued) Tentative structures of identified lignin oligomers in the Kraft lignin sample. RDB: ring double bond equivalent. (Continuation on the next page)

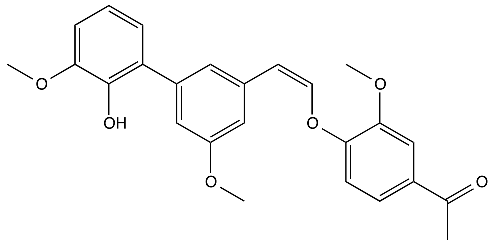

LO 26, m/z 420.1573, $C_{25}H_{24}O_6$, RDB 14

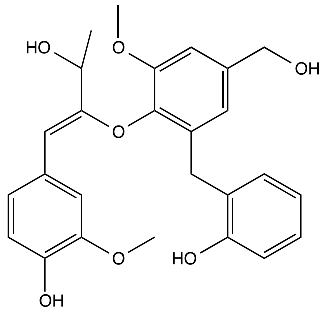

LO 28, m/z 452.1834, $C_{26}H_{28}O_7$, RDB 13

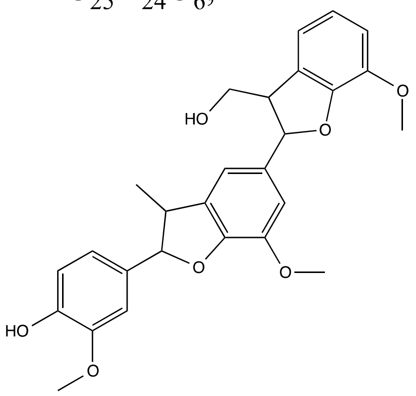

LO 29, m/z 464.1834, $C_{27}H_{28}O_7$, RDB 14

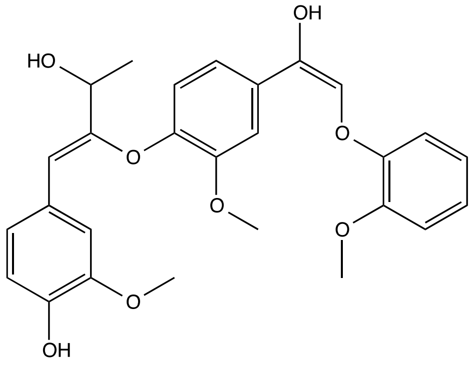

LO 30, m/z 480.1789, $C_{27}H_{28}O_8$, RDB 14

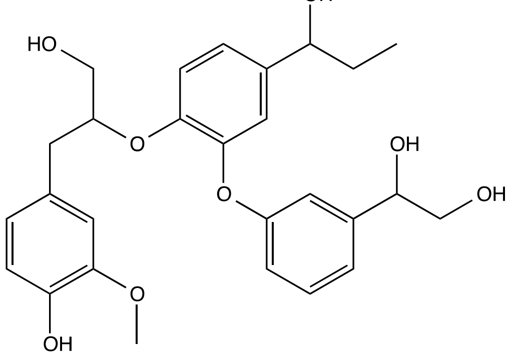

LO 31, m/z 484.2088, $C_{27}H_{32}O_8$, RDB 12

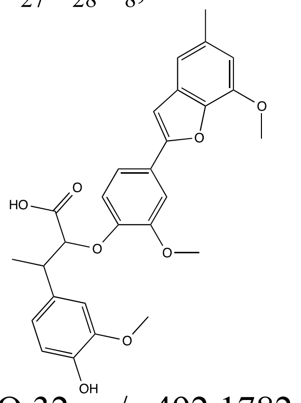

LO 32, m/z 492.1782, $C_{28}H_{28}O_8$, RDB 15

Fig. S7 (continued) Tentative structures of identified lignin oligomers in the Kraft lignin sample. RDB: ring double bond equivalent. (Continuation on the next page)

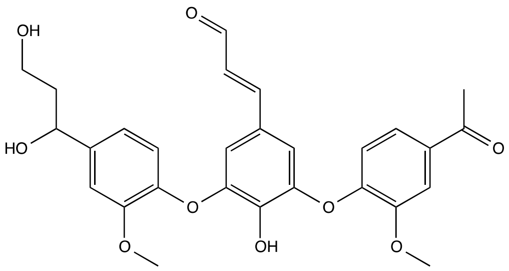

LO 33, m/z 508.1727,
$C_{28}H_{28}O_9$, RDB 15

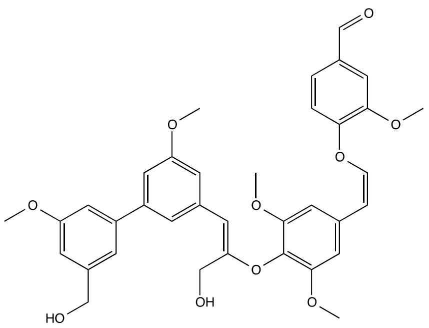

LO 35, m/z 628.2308,
$C_{36}H_{36}O_{10}$, RDB 19

Fig. S7 (continued) Tentative structures of identified lignin oligomers in the Kraft lignin sample. RDB: ring double bond equivalent
